# Genetic architecture of alcohol consumption identified by a genotype-stratified GWAS and impact on esophageal cancer risk in Japanese people

**DOI:** 10.1126/sciadv.ade2780

**Published:** 2024-01-26

**Authors:** Yuriko N. Koyanagi, Masahiro Nakatochi, Shinichi Namba, Isao Oze, Hadrien Charvat, Akira Narita, Takahisa Kawaguchi, Hiroaki Ikezaki, Asahi Hishida, Megumi Hara, Toshiro Takezaki, Teruhide Koyama, Yohko Nakamura, Sadao Suzuki, Sakurako Katsuura-Kamano, Kiyonori Kuriki, Yasuyuki Nakamura, Kenji Takeuchi, Atsushi Hozawa, Kengo Kinoshita, Yoichi Sutoh, Kozo Tanno, Atsushi Shimizu, Hidemi Ito, Yumiko Kasugai, Yukino Kawakatsu, Yukari Taniyama, Masahiro Tajika, Yasuhiro Shimizu, Etsuji Suzuki, Yasuyuki Hosono, Issei Imoto, Yasuharu Tabara, Meiko Takahashi, Kazuya Setoh, The BioBank Japan Project, Koichi Matsuda, Shiori Nakano, Atsushi Goto, Ryoko Katagiri, Taiki Yamaji, Norie Sawada, Shoichiro Tsugane, Kenji Wakai, Masayuki Yamamoto, Makoto Sasaki, Fumihiko Matsuda, Yukinori Okada, Motoki Iwasaki, Paul Brennan, Keitaro Matsuo

**Affiliations:** ^1^ Division of Cancer Epidemiology and Prevention, Department of Preventive Medicine, Aichi Cancer Center Research Institute, Nagoya, Japan.; ^2^ Public Health Informatics Unit, Department of Integrated Health Sciences, Nagoya University Graduate School of Medicine, Nagoya, Japan.; ^3^ Department of Statistical Genetics, Osaka University Graduate School of Medicine, Suita, Japan.; ^4^ Faculty of International Liberal Arts, Juntendo University, Tokyo, Japan.; ^5^ Division of International Health Policy Research, Institute for Cancer Control, National Cancer Center, Tokyo, Japan.; ^6^ Cancer Surveillance Branch, International Agency for Research on Cancer, Lyon, France.; ^7^ Department of Integrative Genomics, Tohoku Medical Megabank Organization, Tohoku University, Sendai, Japan.; ^8^ Center for Genomic Medicine, Kyoto University Graduate School of Medicine, Kyoto, Japan.; ^9^ Department of General Internal Medicine, Kyushu University Hospital, Fukuoka, Japan.; ^10^ Department of Comprehensive General Internal Medicine, Faculty of Medical Sciences, Kyushu University, Fukuoka, Japan.; ^11^ Department of Preventive Medicine, Nagoya University Graduate School of Medicine, Nagoya, Japan.; ^12^ Department of Preventive Medicine, Faculty of Medicine, Saga University, Saga, Japan.; ^13^ Department of International Island and Community Medicine, Kagoshima University Graduate School of Medical and Dental Sciences, Kagoshima, Japan.; ^14^ Department of Epidemiology for Community Health and Medicine, Kyoto Prefectural University of Medicine, Kyoto, Japan.; ^15^ Cancer Prevention Center, Chiba Cancer Center Research Institute, Chiba, Japan.; ^16^ Department of Public Health, Nagoya City University Graduate School of Medical Sciences, Nagoya, Japan.; ^17^ Department of Preventive Medicine, Tokushima University Graduate School of Biomedical Sciences, Tokushima, Japan.; ^18^ Laboratory of Public Health, Division of Nutritional Sciences, School of Food and Nutritional Sciences, University of Shizuoka, Shizuoka, Japan.; ^19^ Department of Public Health, Shiga University of Medical Science, Otsu, Japan.; ^20^ Department of International and Community Oral Health, Tohoku University Graduate School of Dentistry, Sendai, Japan.; ^21^ Division for Regional Community Development, Liaison Center for Innovative Dentistry, Tohoku University Graduate School of Dentistry, Sendai, Japan.; ^22^ Department of Preventive Medicine and Epidemiology, Tohoku Medical Megabank Organization, Tohoku University, Sendai, Japan.; ^23^ Division of Biomedical Information Analysis, Iwate Tohoku Medical Megabank Organization, Iwate Medical University, Iwate, Japan.; ^24^ Department of Hygiene and Preventive Medicine, School of Medicine, Iwate Medical University, Iwate, Japan.; ^25^ Division of Clinical Research and Epidemiology, Iwate Tohoku Medical Megabank Organization, Iwate Medical University, Iwate, Japan.; ^26^ Division of Biomedical Information Analysis, Institute for Biomedical Sciences, Iwate Medical University, Iwate, Japan.; ^27^ Division of Cancer Information and Control, Department of Preventive Medicine, Aichi Cancer Center Research Institute, Nagoya, Japan.; ^28^ Department of Descriptive Cancer Epidemiology, Nagoya University Graduate School of Medicine, Nagoya, Japan.; ^29^ Department of Cancer Epidemiology, Nagoya University Graduate School of Medicine, Nagoya, Japan.; ^30^ Department of Endoscopy, Aichi Cancer Center Hospital, Nagoya, Japan.; ^31^ Department of Gastroenterological Surgery, Aichi Cancer Center Hospital, Nagoya, Japan.; ^32^ Department of Epidemiology, Graduate School of Medicine, Dentistry and Pharmaceutical Sciences, Okayama University, Okayama, Japan.; ^33^ Department of Epidemiology, Harvard T.H. Chan School of Public Health, Boston, MA, USA.; ^34^ Department of Pharmacology, Graduate School of Medicine, Dentistry and Pharmaceutical Sciences, Okayama University, Okayama, Japan.; ^35^ Aichi Cancer Center Research Institute, Nagoya, Japan.; ^36^ Graduate School of Public Health, Shizuoka Graduate University of Public Health, Shizuoka, Japan.; ^37^ Institute of Medical Science, The University of Tokyo, Tokyo, Japan.; ^38^ Department of Computational Biology and Medical Sciences, Graduate School of Frontier Sciences, The University of Tokyo, Tokyo, Japan.; ^39^ Division of Epidemiology, National Cancer Center Institute for Cancer Control, Tokyo, Japan.; ^40^ Department of Health Data Science, Graduate School of Data Science, Yokohama City University, Yokohama, Japan.; ^41^ Division of Cohort Research, National Cancer Center Institute for Cancer Control, Tokyo, Japan.; ^42^ National Institute of Health and Nutrition, National Institutes of Biomedical Innovation, Health and Nutrition, Tokyo, Japan.; ^43^ Division of Ultrahigh Field MRI, Institute for Biomedical Sciences, Iwate Medical University, Iwate, Japan.; ^44^ Iwate Tohoku Medical Megabank Organization, Iwate Medical University, Iwate, Japan.; ^45^ Department of Genome Informatics, Graduate School of Medicine, The University of Tokyo, Tokyo, Japan.; ^46^ Laboratory for Systems Genetics, RIKEN Center for Integrative Medical Sciences, Yokohama, Japan.; ^47^ Laboratory of Statistical Immunology, Immunology Frontier Research Center (WPI-IFReC), Osaka University, Suita, Japan.; ^48^ Integrated Frontier Research for Medical Science Division, Institute for Open and Transdisciplinary Research Initiatives, Osaka University, Suita, Japan.; ^49^ Center for Infectious Disease Education and Research (CiDER), Osaka University, Suita, Japan.; ^50^ Genomic Epidemiology Branch, International Agency for Research on Cancer, Lyon, France.

## Abstract

An East Asian–specific variant on *aldehyde dehydrogenase 2* (*ALDH2* rs671, G>A) is the major genetic determinant of alcohol consumption. We performed an rs671 genotype-stratified genome-wide association study meta-analysis of alcohol consumption in 175,672 Japanese individuals to explore gene-gene interactions with rs671 behind drinking behavior. The analysis identified three genome-wide significant loci (*GCKR*, *KLB*, and *ADH1B*) in wild-type homozygotes and six (*GCKR*, *ADH1B*, *ALDH1B1*, *ALDH1A1*, *ALDH2*, and *GOT2*) in heterozygotes, with five showing genome-wide significant interaction with rs671. Genetic correlation analyses revealed ancestry-specific genetic architecture in heterozygotes. Of the discovered loci, four (*GCKR*, *ADH1B*, *ALDH1A1*, and *ALDH2*) were suggested to interact with rs671 in the risk of esophageal cancer, a representative alcohol-related disease. Our results identify the genotype-specific genetic architecture of alcohol consumption and reveal its potential impact on alcohol-related disease risk.

## Teaser

Genotype-stratified GWAS reveals alcohol consumption loci with varying effects by genotypes and their impact on esophageal cancer.

## INTRODUCTION

Alcohol consumption is a major contributor to mortality and influences risk for various human diseases and disorders (*1*). Even moderate consumption may have a substantial impact on mortality (*2*). The latest Global Burden of Disease study on alcohol use states that the level of consumption should be reduced to zero to minimize health risk (*1*). Alcohol consumption has been considered a heritable trait (*3*, *4*). The number of genetic studies on alcohol consumption is increasing (*5**–**14*), and the genetic variants that are consistently reproducible are those of genes encoding alcohol-metabolizing enzymes (*5*, *7*, *8*, *10**–**14*). Ingested alcohol is predominantly metabolized to acetaldehyde through alcohol dehydrogenase (ADH) enzymes, and aldehyde dehydrogenase (ALDH) enzymes further catalyze the oxidation of acetaldehyde to acetate (*15*). Notably, rs671 (NM_000690.4:c.1510G>A [p.Glu504Lys]), a functional single-nucleotide polymorphism (SNP) in the *ALDH2* gene, which is highly prevalent in East Asians (*16*), is a strong and well-known determinant of alcohol consumption. Every previous genome-wide association study (GWAS) in East Asians (*5*, *7*, *8*, *11*, *14*) identified the strongest signals in the rs671 variant [or variants in strong linkage disequilibrium (LD) with rs671], ranging from *P* < 1.0 × 10^−58^ (*n* = 2834) (*5*) to *P* < 1.0 × 10^–4740^ (*n* = 165,084) (*14*).

Among ALDH isoforms, ALDH2 has by far the highest affinity for acetaldehyde [*K*_m_ (Michaelis constant) < 1 μM] and is primarily responsible for its oxidation (*16*, *17*). Because the *ALDH2* rs671variant inactivates ALDH2 enzymatic activity, individuals who are heterozygous (GA) or homozygous (AA) for this variant experience a rapid accumulation of blood acetaldehyde after alcohol ingestion (*16*). This variant thereby increases exposure to the unpleasant effects of acetaldehyde (e.g., flushing, headache, palpitation, and nausea), which in consequence significantly reduces alcohol consumption and thereby confers a protective effect against alcohol-induced carcinogenesis (*18*). Conversely, heterozygotes (GA) who drink alcohol experience increased susceptibility to carcinogenesis, in particular for head and neck and esophageal cancers, due to higher concentrations of acetaldehyde, one of the most likely carcinogens in alcohol (*15*). With regard to variant homozygotes (AA), however, these have rarely evidenced an increased cancer risk associated with alcohol, because they are unable to oxidize acetaldehyde, a characteristic that is highly correlated with nondrinking (*19*, *20*). In contrast, heterozygotes having 16 to 18% of normal enzyme activity (*21*, *22*) show a broader range of alcohol consumption. Overall, the highest risk group for alcohol-related cancers are heterozygotes (*23*, *24*), and alcohol consumption among genotypes of rs671 shows distinct genetic heterogeneity.

Here, to further decipher the genetic architecture of alcohol consumption in consideration of the status of this unique variant of rs671, we conducted a meta-analysis of rs671 genotype-stratified GWASs comprising up to 175,672 Japanese individuals. Using rs671 genotype-stratified analyses, we tested the hypothesis that variants associated with alcohol consumption exhibit rs671 genotype-dependent associations, and sought previously undiscovered variants conferred by genetic interaction of the rs671 genotype. We considered that this approach might help uncover loci with differential influence on alcohol consumption among genotypes, and enable the detection of loci whose effects were indistinct in previous GWASs. In the present study, we also assessed SNP heritability of alcohol consumption and estimated genetic correlations between rs671 genotypes and between Japanese and European populations. Furthermore, to validate the impact of the discovered loci on alcohol-related disease, we performed a meta-analysis of two esophageal cancer case-control studies. Last, using a completely independent sample (*n* = 24,612), we constructed three types of polygenic risk score (PRS) for alcohol consumption by using the summary statistics of three GWAS meta-analyses: unstratified, rs671 wild-type homozygotes (GG) only, and rs671 heterozygotes (GA) only. We assessed the predictive performances of rs671 alone; PRS by unstratified GWAS; and a combined score of rs671, PRS by rs671 GG-only GWAS, and PRS by rs671 GA-only GWAS and investigated whether the inclusion of gene-gene interaction helps predict alcohol consumption.

## RESULTS

### Characteristics of study participants

A total of 175,672 individuals were included in this GWAS meta-analysis of six Japanese cohorts, namely, the Hospital-based Epidemiologic Research Program at Aichi Cancer Center (HERPACC) Study (*n* = 4958) (*25*), the Japan Multi-Institutional Collaborative Cohort (J-MICC) Study (*n* = 13,236) (*26*, *27*), the Japan Public Health Center-based Prospective (JPHC) Study (*n* = 10,037) (*28*), the Tohoku Medical Megabank Community-Based Cohort (TMM) Study (*n* = 7857) (*29*), the Nagahama Prospective Cohort for Comprehensive Human Bioscience (Nagahama) Study (*n* = 4591) (*30*), and the BioBank Japan (BBJ) Study (*n* = 134,993) (*31*, *32*) after imputation and quality control (QC) of individual subject genotype data (Supplementary Materials and tables S1 and S2). Median self-reported daily alcohol intake, mean age of participants, number of ever/never drinkers, and proportion of male participants were obtained from the studies and are shown in table S1. The number of participants included in each analysis for daily alcohol intake and drinking status was as follows: unstratified analysis, *n* = 154,570 and 175,672; rs671 wild-type homozygotes (GG)–only analysis, *n* = 87,980 and 102,033; heterozygotes (GA)–only analysis, *n* = 55,779 and 62,437; and interaction analysis, *n* = 143,759 and 164,470, respectively (table S3). As the number of variant homozygotes (AA) was small and included only 653 subjects in the ever drinking group (table S3), association analysis in variant homozygotes only was not conducted, and these subjects were excluded from the interaction analyses. The genomic inflation factor, λ, and the LD score regression (LDSC) (*33*) intercepts of the summary statistics for meta-analyses ranged from 1.02 to 1.10 and 1.00 to 1.02, respectively (figs. S1 and S2). These values revealed no evidence of genomic inflation for meta-analyses.

### Unstratified, *ALDH2* rs671 genotype-stratified, and interaction GWAS meta-analyses

**Fig. 1.**
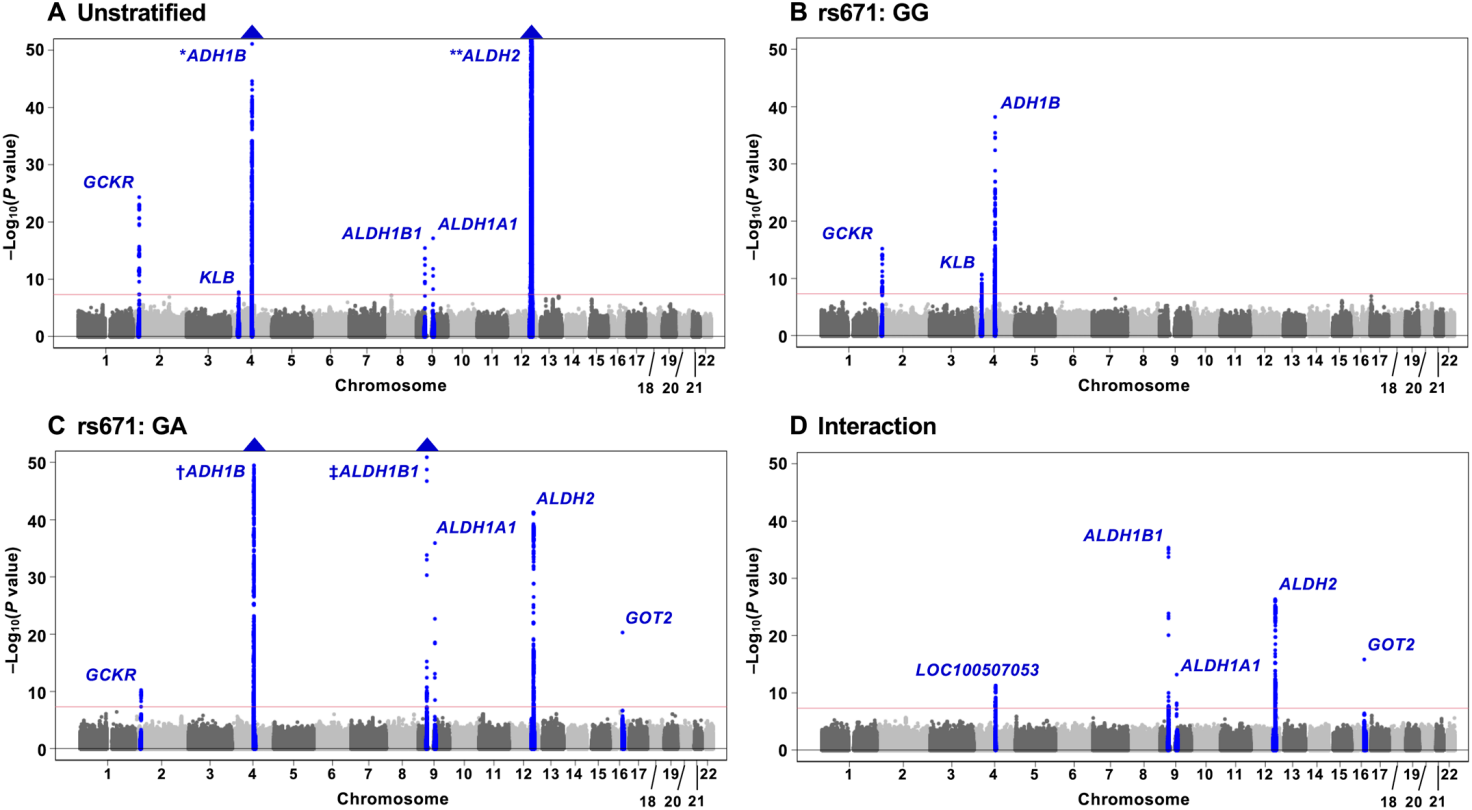
Manhattan plots of the GWAS of daily alcohol intake. The results for (**A**) unstratified, (**B**) rs671 wild-type homozygotes (GG), (**C**) rs671 heterozygotes (GA), and (**D**) interaction with rs671 are shown. The position on each chromosome (*x* axis) and the observed −log_10_(*P* value) (*y* axis) of all tested genetic variants are shown. The solid red line indicates genome-wide significance level. Blue triangles represent loci containing SNPs with *P* values of <1 × 10^−50^. *ADH1B P value (A): 5.89 × 10^−91^; **ALDH2 P value (A): 8.71 × 10^−6987^; ^†^*ADH1B P* value (C): 3.12 × 10^−101^; ^‡^*ALDH1B1 P* value (C): 2.00 × 10^−54^.

**Fig. 2.**
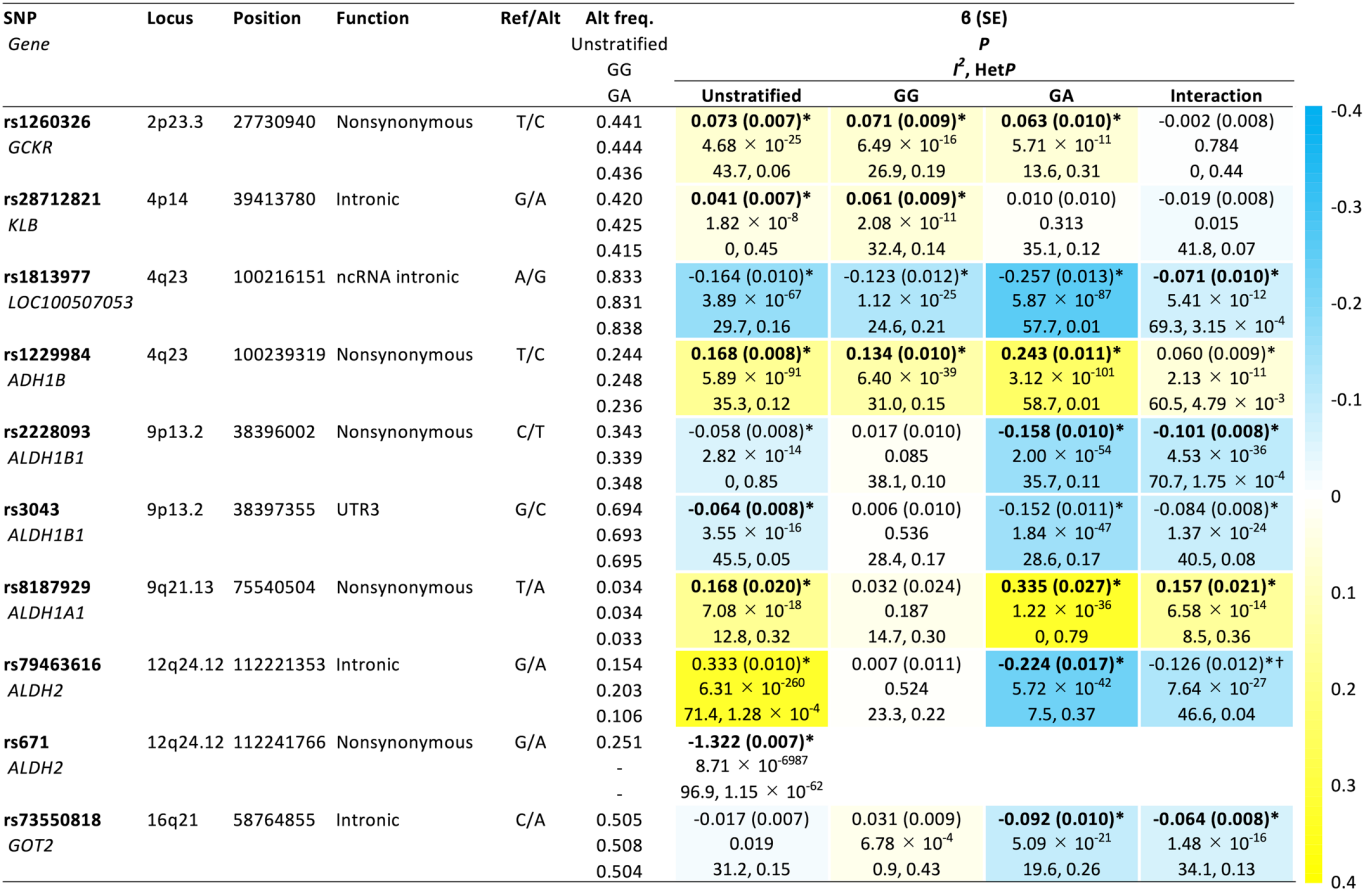
Genomic loci reaching genome-wide significance in either analysis for association with daily alcohol intake. Direction of effects of identified variants other than rs671 is presented as a heatmap. Estimates with a single asterisk show genome-wide significance (*P* < 5.0 × 10^−8^). Lead SNP in each locus is highlighted, with its estimates in bold. SNP, single-nucleotide polymorphism; Ref, reference allele; Alt, alternative allele; freq., frequency; SE, standard error; Het*P*, *P* value from test of heterogeneity. ^†^rs79463616 was not itself lead SNP in the interaction GWAS, but was in perfect LD with the lead SNP, rs4646777 [β (SE) = −0.127 (0.012), *P* = 4.45 × 10^−27^] (*r*^2^ = 1.00 in 1KGP-JPT).

**Fig. 3.**
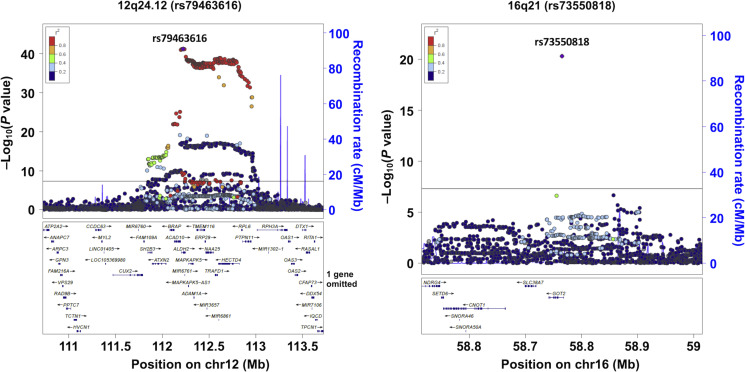
Regional association plots of the identified *ALDH2* and *GOT2* regions. Regional association plots for daily alcohol intake in rs671 heterozygotes are shown. The vertical axis indicates the –log_10_(*P* value) for the assessment of the association of each SNP with daily alcohol intake. Black line represents genome-wide significance threshold of 5.0 × 10^−8^. The colors indicate the LD (r^2^) between each lead SNP and neighboring SNPs based on the JPT population in the 1000 Genomes Project Phase 3.

Major results of the GWAS meta-analyses are summarized in Figs. 1 to 3 and figs. S3 to S6. For daily alcohol intake (Figs. 1 to 3 and fig. S5), the unstratified GWAS (*n* = 154,570) showed six hits (rs1260326 in *GCKR*, rs28712821 in *KLB*, rs1229984 in *ADH1B*, rs3043 in *ALDH1B1*, rs8187929 in *ALDH1A1*, and rs671 in *ALDH2*) (Figs. 1A and 2 and fig. S5A), all of which have been previously reported (*5*, *7*–*14*). In wild-type homozygotes (GG)–only analysis (Fig. 1B; *n* = 87,980), we replicated three known associations, namely, *GCKR* (*9*–*14*), *KLB* (*9*–*11*, *13*), and *ADH1B* (*10*–*14*). In heterozygotes (GA), on the other hand, genome-wide significant associations were obtained in six loci (Fig. 1C; *n* = 55,779). These included four loci that were previously implicated in alcohol consumption, namely, *GCKR* (*9**–**14*), *ADH1B* (*10*–*14*), *ALDH1B1* (*14*), and *ALDH1A1* (*14*). Of the two remaining loci, *GOT2* at 16q21 has not been reported in previous GWASs of alcohol consumption and *ALDH2* at 12q24.12 is the same locus as rs671. The lead SNPs for all identified regions in the genotype-stratified GWAS were rs1260326 (*GCKR*), rs28712821 (*KLB*), and rs1229984 (*ADH1B*) in wild-type homozygotes–only analysis and rs1260326 (*GCKR*), rs1229984 (*ADH1B*), rs2228093 (*ALDH1B1*), rs8187929 (*ALDH1A1*), rs79463616 (*ALDH2*), and rs73550818 (*GOT2*) in heterozygotes-only analysis (fig. S5, B and C, and Fig. 3). A further analysis evaluating variant-rs671 interaction (*n* = 143,759) detected five loci (chromosome 4q23 [LOC100507053], *ALDH1B1*, *ALDH1A1*, *ALDH2*, and *GOT2*) showing genome-wide significant interaction with rs671 (Figs. 1D and 2 and fig. S5D). The lead SNP on chromosome 4q23, rs1813977, is in strong LD with a functional SNP of *ADH1B* (rs1229984) [*r*^2^ and *D*′ values are 0.71 and 1.00 in the 1000 Genomes Project Phase 3–Japanese in Tokyo (1KGP-JPT), respectively]. This is the first identification of the interactive effects of these five loci on rs671, although that of rs1229984 in *ADH1B* at 4q23 on rs671 was suggested from a meta-analysis of studies using candidate gene-based approaches (*34*). Results for drinking status (unstratified, *n* = 175,672; rs671 GG only, *n* = 102,033; rs671 GA only, *n* = 62,437; interaction, *n* = 164,470) were similar to those for daily alcohol intake, except that *GCKR* in heterozygotes-only analysis and *KLB* did not reach genome-wide significance (figs. S3, S4, and S6). Regarding the multiple hits on *ALDH2* observed through these analyses, rs79463616, rs4646777, rs440, and rs4646778 were in perfect LD with one another (all *r*^2^ values are 1.00 in 1KGP-JPT). There was no apparent between-study heterogeneity in any estimates, except for that in rs671 showing profound heterogeneity of I^2^ > 75% (Fig. 2) (*35*); moreover, further application of a random effects model (*36*) did not change the results substantially.

Table S4 shows functional annotation results and allele frequencies across different ancestries for the lead SNPs. Five SNPs, namely, rs1260326 in *GCKR*, rs1229984 in *ADH1B*, rs2228093 in *ALDH1B1*, rs8187929 in *ALDH1A1*, and rs671 in *ALDH2*, are nonsynonymous. According to the 1KGP database, two SNPs (rs8187929 and rs671) are polymorphic, with a minor allele frequency (MAF) of 0.046 and 0.170 in the East Asian population, respectively. In contrast, they are monomorphic in the European population. The *ADH1B* rs1229884 C allele is major in the European population (AF = 0.970) but minor in the East Asian population (AF = 0.300). Table S5 shows allele frequencies for the lead SNPs across different East Asian ancestries. For most lead SNPs, we found similar allele frequencies among these populations.

### Joint meta-analysis

To validate the stratified approach, we applied a joint meta-analysis (JMA) (*37*), an alternative approach that can simultaneously test both the genetic main effect and potential interaction between each variant and rs671 in the meta-analysis context. After careful QC filtering (fig. S7), we identified seven loci to show genome-wide significant associations with daily alcohol intake (fig. S8). Figure S9 shows regional association plots of the identified loci. Instead of the lead SNP rs8187929 in 9q21.13 (Fig. 2), which was not included in the JMA because of its MAF of <5%, we identified rs117986192 (fig. S9 and table S6) within the same locus as rs8187929 (<64 kb distant, r^2^ = 0.45 and *D*′ = 1.00 in 1KGP-JPT). The other lead SNPs were either identical to or in perfect LD with the lead SNPs in the stratified GWAS (Fig. 2), indicating consistent results between the JMA and rs671-stratified GWAS. The JMA results for drinking status (figs. S10 and S11 and table S6) were also consistent with the results of the stratified GWAS for drinking status (figs. S3 and S4).

### Effect of a lead SNP within the same locus as rs671

Figure 2 and fig. S4 show the direction of effects of the identified variants other than rs671 under each analysis. Notably, with regard to the lead SNP on chromosome 12q24.12 (rs79463616 in *ALDH2*, G>A), the A allele of rs79463616, which was associated with decreasing daily alcohol intake in the rs671 heterozygotes (β = −0.224), showed the opposite direction of effect in the unstratified analysis (β = 0.333) (Fig. 2). This apparently conflicting result was due to strong LD between rs79463616 and rs671. The 1KGP-JPT (*n* = 104) and our own direct genotyped data from subjects in the HERPACC Study (*n* = 96) by Sanger sequencing indicated that there were only three rs79463616-rs671 haplotypes, namely, G-G, A-G, and G-A (table S7). The respective LD coefficients of r^2^ and *D*′ were <0.1 and 1.0 (table S7). Similarly, within 4623 subjects in the HERPACC Study, TaqMan-based genotyping demonstrated that rs56884502, which is in perfect LD with rs79463616 (r^2^ = 1 in 1KGP-JPT), was in complete LD with rs671 (r^2^ = 0.073; *D* = 1) (table S8). We genotyped rs56884502 here as a proxy locus of rs79463616 since rs79463616 was unsuitable for polymerase chain reaction (PCR)–based genotyping due to its presence within repetitive regions.

Results from LD analysis based on the 1KGP-JPT data (*n* = 104) of rs671 and the 70 SNPs at 12q24.12, which exhibited a *P* value of <1 × 10^−30^ for daily alcohol intake among the heterozygotes (table S9), are shown in fig. S12. The pairwise *D′* figure showed that all 70 SNPs were in near-complete LD in terms of *D′* (fig. S12). Furthermore, the three indicated haplotypes of rs671 and these SNPs could explain >98% (fig. S13). In addition, within subjects in the HERPACC Study (*n* = 982), our own direct genotyping of rs671 and the six rs79463616 proxy SNPs of rs56884502, rs4648328, rs440, rs441, rs4646777, and rs4646778 using Fluidigm SNPtype assays showed that three haplotypes could explain 99.8% (table S8). Therefore, when evaluated without stratification, rs79463616 A allele, which formed a haplotype with rs671 G allele only, was associated with increasing drinking intensity, by reflecting the effect of rs671 G allele. However, when stratified, rs79463616 A allele turned out to have the opposite direction of effect—decreasing drinking intensity. The other lead SNP on chromosome 12q24.12 for drinking status (rs440) was in perfect LD with rs79463616 and accordingly showed the same phenomenon (fig. S4).

### Conditional analyses

Conditional analyses for the genome-wide significant loci excluding 12q24 for daily alcohol intake in rs671 heterozygotes identified two additional hits on chromosome 4q23 (rs13125415) and 9p13.2 (rs3043) (table S10). Haplotype estimation indicated that rs13125415 and rs3043 show moderate (*D′* = 0.62) and complete (*D′* = 1.00) LD with the respective lead SNP of each region (i.e., rs1229984 and rs2228093) (table S11). Specifically, additional LD analysis of rs2228093 and the seven SNPs at 9p13.2, which showed a *P* value of <1 × 10^−30^ for daily alcohol intake in the heterozygotes, demonstrated substantial LD among these SNPs, and three haplotypes accounted for >89% of the seven observed haplotypes (fig. S14). This may indicate that these loci, especially chromosome 9p13.2, exhibit a similar phenomenon seen at 12q24.12 and should be further evaluated as a haplotype-phenotype association.

### Heritability and cross-group (rs671 GG versus GA) genetic correlation

Using LDSC (*33*), we assessed SNP heritability of alcohol consumption and estimated a genetic correlation to investigate whether alcohol consumption genetically differed across rs671 genotypes. We identified an SNP heritability of daily alcohol intake (drinking status) of 5.23% (4.41%) and 7.70% (6.98%) for the rs671 GG and GA analyses, respectively (table S12). Subsequent analyses of cross-group (rs671 GG versus GA) genetic correlation (*r*_g_) suggested that alcohol consumption genetically differed across rs671 GG and GA individuals [daily alcohol intake: *r*_g_ = 0.716, *P* (*r*_g_ = 1) = 0.033; drinking status: *r*_g_ = 0.713, *P* (*r*_g_ = 1) = 0.018] (table S12).

### Associations of previously reported loci

The results of the associations of the 82 previously reported loci in the European population (*9*, *11*, *12*, *38*, *39*) are reported in table S13. Among these, we successfully replicated the genome-wide significance of three loci (*GCKR*, *KLB*, and *ADH1B*) in the unstratified and wild-type homozygotes-only analyses and two loci (*GCKR* and *ADH1B*) in the heterozygotes-only analysis. In addition, we found nominal evidence of association (*P* < 0.05) for 13 other loci in the unstratified analysis. Upon stratification, however, we observed nominal associations at 14 other loci in wild-type homozygotes, whereas only at 5 in heterozygotes. Of the remaining loci, the 13 SNPs were either monomorphic or extremely rare (MAF < 0.002) in the Japanese population, resulting in no observed association in our GWAS.

### Lookups of our identified loci in the GSCAN data

We performed lookups of our identified lead SNPs in the GWAS and Sequencing Consortium of Alcohol and Nicotine Use (GSCAN) data (*12*), comprising 941,280 individuals of European ancestry. The results are reported in table S14. While all three loci (*GCKR*, *KLB*, and *ADH1B*) identified in the rs671 GG-only analysis showed a genome-wide significant association, we did not observe any association in the other loci identified in the rs671 GA-only analysis, including two loci (*ALDH2* and *GOT2*), which have not been reported in the context of alcohol consumption.

### Cross-ethnic genetic correlation

We computed cross-ethnic genetic-impact correlation (ρ_gi_) between Japanese and European (*12*) populations using Popcorn, where ρ_gi_ represents the correlation coefficient of the population-specific allele variance–normalized SNP effect sizes (*40*). The genetic-impact correlation was defined as the correlation of effect sizes after genotypes were normalized to have mean 0 and variance 1. While Japanese individuals with the rs671 GG genotype may share a similar genetic architecture for alcohol consumption with Europeans [ρ_gi_ = 0.975, *P* (ρ_gi_ = 1) = 0.905], results indicated that Japanese individuals with the rs671 GA genotype may genetically differ from Europeans in terms of alcohol use [ρ_gi_ = 0.287, *P* (ρ_gi_ = 1) = 0.0003] (table S15).

### eQTL analysis of lead SNPs in *ALDH2* and *GOT2* loci

Of the detected variants, rs79463616, rs4646777, rs440, and rs4646778 in *ALDH2* and rs73550818 in *GOT2* were found to be expression quantitative trait locus (eQTL) using the Genotype-Tissue Expression (GTEx) database (table S16). rs79463616 A allele, rs4646777 A allele, rs440 C allele, and rs4646778 A allele are associated with decreased expression of *ALDH2* in multiple tissues. rs73550818 A allele is associated with increased expression of *GOT2* in liver (*P* = 1.0 × 10^−8^).

### Esophageal cancer case-control study

Given that esophageal cancer, especially squamous cell carcinoma, which is a predominant subtype in East Asians, is strongly associated with alcohol consumption and shows sufficient evidence for a gene-environment interaction between *ALDH2* and alcohol drinking (*41*), esophageal cancer represents a suitable target to assess the impact of the seven identified variants in the rs671 genotype-stratified GWAS (rs1260326 in *GCKR*, rs28712821 in *KLB*, rs1229984 in *ADH1B*, rs2228093 in *ALDH1B1*, rs8187929 in *ALDH1A1*, rs79463616 in *ALDH2*, and rs73550818 in *GOT2*) on alcohol-related disease risk. We therefore performed a meta-analysis of two independent esophageal cancer case-control studies conducted within the HERPACC Study (692 cases and 995 controls) and the BBJ Study (416 cases and 86,515 controls). Because the lead SNP on chromosome 12q24.12, rs79463616, present within highly repetitive regions, was not suitable for PCR-based genotyping, we analyzed rs4648328, which showed the second-lowest *P* value (*P* = 5.8 × 10^−42^; table S9) and is in perfect LD with rs79463616 (r^2^ = 1.00 in 1KGP-JPT) as a proxy locus of rs79463616. Table S17 shows the distribution of age, sex, and rs671 genotype among cases and controls in each study. Table S18 shows study-specific information on the alternative allele frequency and imputation quality score of each SNP.

**Fig. 4.**
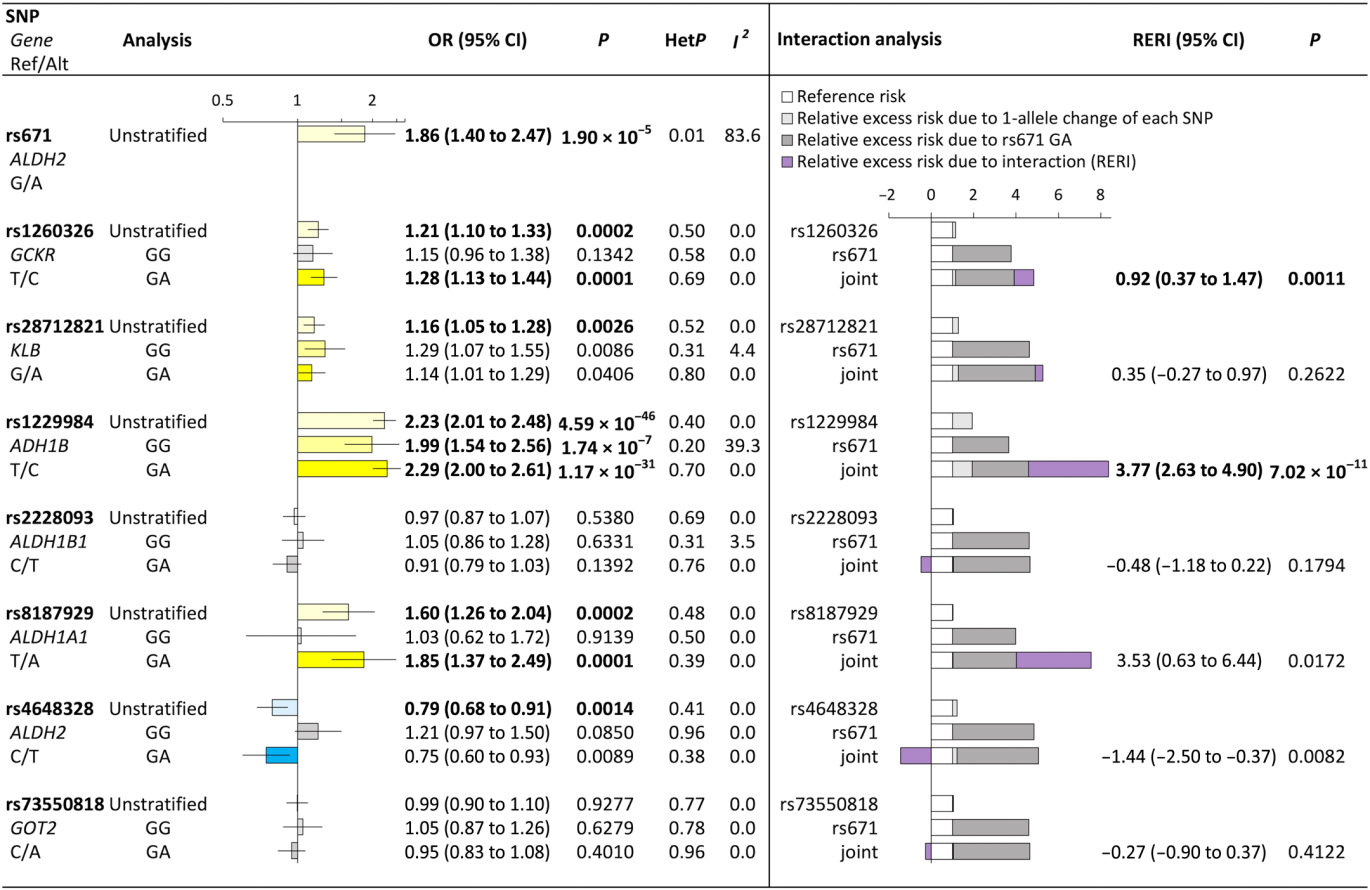
Impact of identified variants on esophageal cancer risk and assessment of additive interaction of each SNP (1-allele change) with rs671 (GA versus GG). ORs for esophageal cancer per 1-allele change in eight SNPs on three different subject groups [entire population (unstratified), subjects with the rs671 GG genotype only (GG), and subjects with the rs671 GA genotype only (GA)] were calculated by a random effects model by pooling study-specific ORs adjusted for sex, age, the first 10 principal components (for the BBJ Study), and study version (for the HERPACC Study). Stacked bar charts on assessment of additive interaction are presented as ORs partitioned into relative excess risks due to 1-allele change in each SNP other than rs671 (light gray), rs671 (GA versus GG) (gray), and their interaction (RERI) (purple). Statistical significance was set at the Bonferroni corrected threshold of *P* < 0.05/8 (=0.00625), and suggestive significance was set at *P* < 0.05. RERI was considered to achieve suggestive significance (*P* < 0.05) when its confidence interval did not include 0. Estimates in bold show statistical significance with Bonferroni correction (*P* < 0.00625). The HERPACC Study included 692 cases and 995 controls; the BBJ Study included 416 cases and 86,515 controls. OR, odds ratio; CI, confidence interval; BBJ, BioBank Japan; HERPACC, Hospital-based Epidemiologic Research Program at Aichi Cancer Center.

Figure 4 shows summary odds ratios (ORs) per 1-allele change in each SNP in three different subject groups [entire population (unstratified), subjects with the rs671 GG genotype only (GG), and subjects with the rs671 GA genotype only (GA)], along with interaction on an additive scale [relative excess risk due to interaction (RERI)] between each SNP and rs671. Given the additive scale’s biological and public health importance (*42*), this study primarily focused on interaction on the additive scale. Table S19 shows summary estimates of the ORs for each SNP (1-allele change), ORs for rs671 (GA versus GG), and joint ORs for each SNP and rs671, which were used for the estimation of RERI between each SNP and rs671. Table S19 also includes the results of multiplicative interaction tests. In the unstratified analysis, all variants except *ALDH1B1* rs2228093 and *GOT2* rs73550818 showed significant associations (*P* < 0.05/8 = 0.00625) with esophageal cancer risk. Upon stratification, *KLB* rs28712821 and *ADH1B* rs1229984 showed suggestive significant (*P* < 0.05) and significant associations, respectively, in wild-type homozygotes (GG)–only analysis, whereas in heterozygotes (GA)–only analysis, we observed significant ORs in *GCKR* rs1260326, *ADH1B* rs1229984, and *ALDH1A1* rs8187929 and suggestive significant ORs in *KLB* rs28712821 and *ALDH2* rs4648328. The direction of effects on esophageal cancer risk was consistent with the direction of effects on daily alcohol intake (Fig. 2) for all SNPs in both analyses of the wild-type homozygotes and heterozygotes. Moreover, although multiplicative interaction was significant only in rs4648328 (*ALDH2*) (table S19), the additive scale identified significant interaction with rs671 in *GCKR* rs1260326 and *ADH1B* rs1229984 and suggestive significant interaction in *ALDH1A1* rs8187929 and *ALDH2* rs4648328 (Fig. 4). For example, a RERI of 3.77 [95% confidence interval, 2.63 to 4.90] in rs1229984 means that the relative risk of esophageal cancer in the subjects with rs671 GA genotype is 3.77 more per 1-allele change in rs1229984 than the sum of the individual effects of these SNPs. Further analysis restricted to cases with squamous cell carcinoma showed consistent results (fig. S15). Because we evaluated common variants (table S18), concern regarding elevated type 1 error rates due to unbalanced case-control ratios (*43*) within the BBJ Study is likely to be small. To ascertain this, we performed two sensitivity analyses. First, we used a Firth logistic regression model, an alternative model that is robust to type 1 error rate inflation under unbalanced case-control ratios (*44*), in the BBJ Study. Second, we subsampled controls and changed the case-control ratio to 1:10 in the BBJ Study. Although there were slight changes in some estimates, we observed consistent associations among the three types of analyses (table S20).

### Polygenic risk scoring

The scheme of the PRS analysis is presented in fig. S16. We performed the PRS analysis among 24,612 newly genotyped individuals in the J-MICC Study, all with direct genotype data of rs671, who randomly assigned to either the target or validation datasets in a 2:1 ratio (tables S3 and S21). On the basis of the respective target dataset consisting of all 16,408 individuals with either rs671 GG, GA, or AA genotypes, 9124 wild-type homozygotes, and 6134 heterozygotes, we constructed three types of PRS for daily alcohol intake using the summary statistics of the unstratified (PRS_unstratified_), rs671 GG-only (PRS_GG_), and rs671 GA-only (PRS_GA_) GWAS meta-analyses. We then assessed how well PRS_unstratified_, PRS_GG_, and PRS_GA_ predicted phenotype [i.e., log_2_ (daily alcohol intake + 1)] as a predictive power of R^2^ (%) on the validation dataset within 8204 individuals with either rs671 GG, GA, or AA genotypes, 4562 wild-type homozygotes, and 3067 heterozygotes, respectively. As shown in table S21, PRS_unstratified_, PRS_GG_, and PRS_GA_ exhibited significant predictive power, accounting for 15.85, 0.55, and 1.76% of the variance in alcohol consumption in their respective validation dataset. Of note, in all 8204 individuals in the validation dataset, the R^2^ of rs671 alone was 19.22% (table S22), indicating that rs671 alone had better predictive power than PRS_unstratified_ (fig. S17). To investigate whether the inclusion of gene-gene interaction helps predict alcohol consumption, we further constructed a combined score of rs671, PRS_GG_, and PRS_GA_ by linear regression analysis using the target dataset consisting of 15,258 individuals with either the rs671 GG or GA genotypes (fig. S16). We then assessed the predictive performances of rs671 alone, PRS_unstratified_, and the combined score in 7629 individuals with either rs671 GG or GA genotypes in the validation dataset. As shown in table S23 and fig. S17, the respective R^2^ values of rs671 alone, PRS_unstratified_, and the combined score were 12.16, 9.18, and 13.01%, suggesting that predictive power could be improved over that of rs671 alone or PRS_unstratified_ when gene-gene interactions are taken into account.

## DISCUSSION

We report here the results of an rs671 (G>A) genotype-stratified GWAS meta-analysis of alcohol consumption with a total of 175,672 participants from six Japanese cohorts. An overview summarizing this study is shown in fig. S18. We identified three loci (*GCKR*, *KLB*, and *ADH1B*) in wild-type homozygotes (GG) and six loci (*GCKR*, *ADH1B*, *ALDH1B1*, *ALDH1A1*, *ALDH2*, and *GOT2*) in heterozygotes (GA). Of these, two loci (*ALDH2* at 12q24.12 and *GOT2* at 16q21) were revealed for the first time to affect alcohol consumption after consideration of rs671 genotype. The interaction GWAS with rs671 identified five genome-wide significant loci (*ADH1B*, *ALDH1B1*, *ALDH1A1*, *ALDH2*, and *GOT2*). Subsequent genetic correlation analyses revealed ancestry-specific genetic architecture in heterozygotes. The esophageal cancer case-control study further demonstrated that the effects of most identified loci were strong enough to influence not only drinking behavior but also esophageal cancer risk. Of these, four (*GCKR*, *ADH1B*, *ALDH1A1*, and *ALDH2*) were found to potentially interact with rs671 on the risk of esophageal cancer.

The rs671 genotype-stratified GWAS successfully uncovered loci with differential influence on alcohol consumption between genotypes. The wild-type homozygotes-only analysis revealed the association of *GCKR*, *KLB*, and *ADH1B* with alcohol consumption, mirroring the consistent observation of these loci in previous GWASs among non-Asian populations (*9**–**13*), where rs671 is often monomorphic. The lookups in the GSCAN data (table S14) showed genome-wide significant association in all loci identified in our wild-type homozygotes–only analysis versus no association in any loci identified in the heterozygotes-only analysis. These results are in good agreement with the fact that the GSCAN consortium included individuals of European ancestries. The heterozygotes-only analysis detected the strongest signal for daily alcohol intake in *ADH1B* (*P* = 3.1 × 10^−101^), followed by *ALDH1B1* (*P* = 2.0 × 10^−54^), *ALDH2* (*P* = 5.7 × 10^−42^), and then *ALDH1A1* (*P* = 1.2 × 10^−36^), all of which are associated with the concentration of acetaldehyde. ADH1B is the predominant isoform involved in alcohol oxidation, whereas ALDH1B1 and ALDH1A1 are ALDH isoforms involved in acetaldehyde oxidation, with the second (*K*_m_ of 30 μM) and third (*K*_m_ of 50 to 180 μM) highest affinities for acetaldehyde, respectively (*17*). This finding genetically suggests that, at least in this population, acetaldehyde plays a substantial role in determining alcohol consumption levels in rs671 heterozygotes. The nonsynonymous lead SNP of rs1229984 (NM_000668.6:c.143A>G [p.His48Arg]) found in the *ADH1B* coding region is associated with slow alcohol metabolism, leading to the slow accumulation of acetaldehyde and consequently greater alcohol consumption (*19*, *20*). Although rs2228093 (NM_000692.5:c.257C>T [p.Ala86Val]) in *ALDH1B1* and rs8187929 (NM_000689.5:c.529A>T [p.Ile177Phe]) in *ALDH1A1* were first shown to be associated with drinking status in a previous Japanese GWAS (*14*), their effects on enzyme activity are not fully understood. However, rs2228093 in *ALDH1B1* was also shown to possibly influence alcohol consumption in European populations using a candidate gene approach (*45*, *46*). In addition, the protective effect of rs2228093 T allele against alcohol consumption observed in this study is consistent with the results of previous studies using bioinformatic analyses, which predicted disruption of the structural flexibility of the protein product (*47*) and catalytic inactivity (*48*) of ALDH1B1 in the presence of the rs2228093 T allele. Elucidating the functional contributions of rs2228093 in *ALDH1B1* and rs8187929 in *ALDH1A1* to alcohol/aldehyde metabolism requires further investigation.

Using a stratified method based on rs671 genotype, we were able to uncover the effect of a lead SNP at the same locus as rs671. ALDH2 works as a tetramer, which is regarded as a dimer of dimers. The rs671 A allele is predicted to disrupt the structure of not only its own subunit but also its dimer partner, reducing the stability of the tetramic structure of ALDH2 and resulting in a dramatic reduction in enzyme activity (*49*). This East Asian–specific SNP is considered to be a relatively young variant (*50*) and to have been under strong recent natural selection pressure in the Japanese population (*51*). On the other hand, the lead SNP at 12q24.12 of rs79463616 is a globally common SNP located ≈20 kb distant to rs671 (table S4). Given this evidence and the two LD measures of r^2^ < 0.1 and *D′* = 1.0 for rs79463616 and rs671, we speculate that rs79463616 arose before rs671 and that rs671 then arose on a different branch from rs79463616 in the rs79463616-rs671 G-G haplotype without subsequent historic recombination, finally resulting in the three haplotypes of G-G, A-G, and G-A. The protective effect of the rs79463616 A allele against alcohol consumption observed in rs671 heterozygotes can therefore be regarded as a protective effect of the rs79463616-rs671 G-A/A-G diplotype. rs79463616 is located in the intron of *ALDH2* (table S4), and the rs79463616 A allele was found to be associated with decreased expression of *ALDH2* (table S16). Furthermore, none of the other 69 SNPs at 12q24.12 that were in LD with rs79463616 and showed *P* < 1 × 10^−30^ for daily alcohol intake is located within the coding region (table S9), suggesting that while these SNPs may be potentially associated with expression, they may have no direct effect on the protein structure or tetramer formation of ALDH2 by interacting with the rs671 A allele on the opposite haplotype. Although this potential effect on *ALDH2* expression is inconsistent with the lack of protective effect of the rs79463616 A allele in a population with rs671 wild-type homozygosity, one can explain that this protective effect may be too small for detection in wild-type homozygotes. Further elucidation of this rs671-dependent protective effect of rs79463616 will require deep whole-genome sequencing–based analysis and/or experimental studies.

The remaining identified locus associated with alcohol consumption in this study is *GOT2*. The lead SNP (rs73550818, C>A) is located in the intron of *GOT2* and showed a protective effect against alcohol consumption in heterozygotes; this may be a suitable target for future study, given that the rs73550818 A allele was shown to be significantly associated with increased levels of aspartate aminotransferase (AST), a biochemical marker for liver injury, in a previous GWAS of 134,154 Japanese individuals (*52*). *GOT2* encodes the mitochondrial AST (mAST); this plays an important role in many processes, including amino acid metabolism, long-chain fatty acid uptake, and the urea and tricarboxylic acid cycles (*53*). An in vivo study suggested that increased mAST among alcoholics is a consequence of the pharmacologic up-regulation of *GOT2* gene expression by ethanol, which further mediates fatty acid uptake, resulting in alcoholic fatty liver (*53*). Considering that the rs73550818 A allele is associated with increased expression of *GOT2* in the liver (table S16), this allele might be associated with ethanol-induced liver injury. On the other hand, previous studies of rs671 showed significantly lower AST in heterozygotes than in wild-type homozygotes among drinkers (*54*), even after adjustment for alcohol intake (*7*). An observational study of patients with alcoholic liver injury (*55*) and a study of *Aldh2* knockout mice (*56*) suggested a protective effect of the rs671 A allele on ethanol-induced liver injury. These findings suggest the opposite effects of the rs73550818 A and rs671 A alleles on ethanol-induced liver injury. The mechanism of the suggested interaction between rs73550818 and rs671 observed in our present study therefore warrants further investigation.

We found that only a few of the alcohol use–associated loci reported in Europeans (*12*) were nominally significant in this study (table S13). This may contradict the latest multi-ancestry GWAS meta-analysis of alcohol use, which demonstrated that most associated genetic variants showed homogeneous effect size estimates across diverse ancestries (*57*). To explore this, we performed cross-ethnic genetic correlation analysis and revealed that Japanese individuals with the rs671 GG genotype share a similar genetic architecture for alcohol consumption with Europeans, while those with the rs671 GA genotype exhibited genetic differences (table S15). These findings may be interpreted to mean that we have identified an ancestry-specific genetic architecture by accounting for the presence of rs671 polymorphism, specifically rs671 GA. The reason why we failed to replicate most of the GSCAN results in our rs671 GG-only analysis despite a similar genetic correlation with Europeans can be mainly explained by a lack of power due to the limited number of individuals with the GG genotype (daily alcohol intake; *n* = 87,980) compared with the GSCAN (*n* = 941,280). Further investigations using LDSC analysis revealed that SNPs other than rs671 made substantial contributions to determining drinking behavior among the Japanese population, with SNP heritability estimates of ~5.23% and ~7.70% for the rs671 GG- and GA-only analyses, respectively (table S12). The cross-group (rs671 GG versus GA) genetic correlation indicated that there were genetic differences related to alcohol consumption across rs671 GG and GA individuals (table S12). Together, this study uncovered an rs671 genotype-specific genetic architecture of alcohol consumption among the Japanese population.

Of the seven identified variants in the rs671 genotype-stratified GWAS, five variants (rs1260326 in *GCKR*, rs28712821 in *KLB*, rs1229984 in *ADH1B*, rs8187929 in *ALDH1A1*, and rs79463616 in *ALDH2*) were shown to be associated with esophageal cancer risk, exhibiting the same direction of association as the genotype-stratified GWAS. Although the impact of *ADH1B* on esophageal cancer risk is well documented (*58*, *59*), this is the first identification of the genetic contribution of the other four (*GCKR*, *KLB*, *ALDH1A1*, and *ALDH2*) to esophageal cancer risk. The rs671 genotype stratification revealed that contributions to esophageal cancer risk from *GCKR*, *ALDH1A1*, and *ALDH2* might be observable exclusively among heterozygotes. The interaction analysis further highlighted the potential combined effect of *GCKR*, *ADH1B*, *ALDH1A1*, and *ALDH2* with rs671 GA toward increased (*GCKR*, *ADH1B*, and *ALDH1A1*) or decreased (*ALDH2*) risk of esophageal cancer and thereby carries important implications for personalized prevention.

As alcohol consumption is associated with various diseases and disorders, we also performed a phenome-wide association study (PheWAS) to assess the associations of the discovered loci with diverse phenotypes other than esophageal cancer (“PheWAS” section of the Supplementary Methods, tables S24 and S25, and fig. S19). The PheWAS results indicated that drinking behavior shares genetic architecture with multiple phenotypes and unveiled associations in the risk of certain phenotypes, with specific variants showing interaction with rs671. These results are worth evaluation in future studies and may contribute to the construction of further evidence for personalized prevention based on gene-gene interaction with rs671. Further studies are needed, including validation in other populations and mechanistic investigation.

In our PRS analysis, we observed that rs671 alone had better predictive power than PRS_unstratified_. Because of interactions with rs671, there are genetic variants that have the opposite direction of effect depending on the genotype combination, as observed at the *ALDH2* locus, as well as variants that differ in effect size on alcohol consumption among rs671 genotypes (Fig. 2). We therefore speculate that PRS_unstratified_, which takes account of the main effects only, could not accurately predict alcohol consumption; rather, it resulted in reduced predictive power. Further comparison of the predictive performances of rs671 alone, PRS_unstratified_, and the combined score of rs671, PRS_GG_, and PRS_GA_ suggested that the inclusion of gene-gene interaction is beneficial for predicting phenotype, compared to a PRS based solely on main effects. From these results, it can be inferred that the suboptimal predictive power of PRS in general may in part be due to the complex interplay of gene-gene interactions, as we observed with rs671, particularly those that can exert an effect in the opposite direction depending on the gene-gene combination. We postulate that these insights provide substantial implications for the future construction of PRS.

This study has several strengths. First, this study included the largest number of Japanese individuals, derived from major large-scale cohorts in Japan. This allowed us to conduct GWASs stratified by genotypes. Second, the study involved a single ethnic group with a similar religious and cultural background, making it unlikely that these factors would bias the phenotype of alcohol consumption (*60*). Several potential limitations also warrant mention. An important limitation is that data on alcohol consumption were self-reported. Nevertheless, these data were collected at baseline survey using validated questionnaires or their variants in all studies. Any misclassification bias is therefore likely to be nondifferential, in which case the validity of our observed associations is likely to hold. Second, we focused on Japanese individuals living in Japan, although this is also one of the strengths of this study, as mentioned. Although we can assume that different East Asian ancestries share a similar genetic background for most lead SNPs (table S5), drinking behavior is known to be influenced by sociocultural factors, aging, and sex (*61*–*63*) in addition to religious and cultural background (*60*). These factors enhance/attenuate drinking behavior, possibly leading to enhanced/attenuated power to detect the association between the lead SNPs and drinking behavior even within a similar genetic background. The only way to investigate this point is to analyze actual data across several countries and populations, and this should be a goal of future international collaborative studies. In addition, it would be interesting to explore whether the observed effect of rs671 is applicable to East Asians living abroad, where lifestyle and culture are far different. However, even in the UK Biobank, one of the largest population-based recourses, there is only a limited number of East Asian samples (approximately 2500) (*64*). A simulation study has shown that GWAS with such a small sample size would be significantly underpowered, limiting the ability to detect variants with moderate to low effect sizes (*65*). Therefore, whether the observed effect of rs671 is specific to Japanese is an open-ended question, and further large-scale international collaborative investigation is warranted.

Last, we would like to note a merit of this particular type of genotype-stratified GWAS. If there is a phenotype of interest and a variant that has a strong influence on that phenotype—in this case, alcohol consumption is the phenotype on which *ALDH2* rs671 has a decisive effect—this method is highly effective. The fact that many of the variants revealed in this study are related to alcohol metabolism may strongly support this notion. Although GWAS was originally conceived as hypothesis-free, the hypothesis-driven approach we used here worked effectively, indicating its potential in the search for previously undiscovered targetable loci. A phenomenon observed in this study is generally termed gene-gene interaction or SNP-SNP interaction, and its existence has been identified using the candidate approach (*66*). GWASs examining interactions using a statistical interaction term have not necessarily been successful: In this study, the interaction term approach worked because of the use of a strong partner, rs671. However, it seemed less efficient compared with the genotype-stratifying approach. In addition, although the JMA approach identified loci, which were consistent with the stratified GWAS approach, the stratified approach more intuitively depicts the phenomenon of interaction than JMA, as shown in Fig. 1. Moreover, applying the JMA in this context requires greater skill and experience in tuning the settings to control false positives (fig. S7), and in this regard also, the stratified GWAS appears advantageous compared to JMA. Accordingly, we propose that hypothesis-based genotype-stratified GWAS represents a promising alternative approach to discovery.

In conclusion, we performed an *ALDH2* rs671 genotype-stratified GWAS and successfully identified several loci that were associated with alcohol consumption in an rs671-dependent manner. This study further reveals ancestry-specific genetic architecture of alcohol consumption and its impact on esophageal cancer, a representative alcohol-related diseases, which should in turn deepen our knowledge of the pathogenesis of alcohol-related diseases and disorders.

## MATERIALS AND METHODS

### Study subjects and genotyping

We performed a genome-wide meta-analysis based on the Japanese Consortium of Genetic Epidemiology (J-CGE) studies (*67*), the Nagahama Study (*30*), and the BBJ Study (*31*, *32*). The J-CGE consisted of the following Japanese population-based and hospital-based studies: the HERPACC Study (*25*), the J-MICC Study (*26*, *27*), the JPHC Study (*28*), and the TMM Study (*29*). Individual study descriptions and an overview of the characteristics of the study populations are provided in the Supplementary Materials and table S1. Data and sample collection for the participating cohorts were approved by the respective research ethics committees. All participating studies obtained informed consent from all participants by following the protocols approved by their institutional ethical committees.

### Phenotype

Information on alcohol consumption was collected by questionnaire in each study. Because the questionnaires were not homogeneous across the studies, we harmonized the two alcohol consumption phenotypes of drinking status (never versus ever drinker) and daily alcohol intake (grams/day) in accordance with each study’s criterion. Details are provided in the Supplementary Materials.

### QC and genotype imputation

QC for samples and SNPs was performed based on study-specific criteria (table S2). Genotype data in each study were imputed separately based on the 1000 Genomes Project reference panel (phase 3, all ethnicities) (*68*). Phasing was performed with the use of SHAPEIT (v2) (*69*) and Eagle (*70*), and imputation was performed using minimac3 (*71*), minimac4, or IMPUTE (v2) (*72*). Information on the study-specific genotyping, imputation, QC, and analysis tools is provided in table S2. After genotype imputation, further QC was applied to each study. SNPs with an imputation quality of *r*^2^ < 0.3 for minimac3 or minimac4, info < 0.4 for IMPUTE2, or a MAF of <0.01 were excluded.

### Association analysis of SNPs with daily alcohol intake and drinking status

Association analysis of SNPs with daily alcohol intake and drinking status was performed on three different subject groups: the entire population, subjects with the rs671 GG genotype only, and subjects with the rs671 GA genotype only. Because the number of ever drinkers with the rs671 AA genotype was too small (table S3), association analysis in subjects with the rs671 AA genotype only was not conducted. Daily alcohol intake was base-2 log-transformed [log_2_ (grams/day + 1)]. The association of daily alcohol intake with SNP allele dose for each study was assessed by linear regression analysis with adjustment for age, age^2^, sex, and the first 10 principal components. For the BBJ Study, the affection status of 47 diseases was further added as covariates. The association of drinking status with SNP allele dose for each study was assessed by logistic regression analysis with adjustment for age, age^2^, sex, the first 10 principal components, and disease affection status of 47 diseases (for the BBJ Study). The effect sizes and SEs estimated in the association analysis were used in the subsequent meta-analysis. The association analysis was conducted using EPACTS (http://genome.sph.umich.edu/wiki/EPACTS), SNPTEST (*73*), or PLINK2 (*74*). Association analysis, including interaction terms, was performed to evaluate the differential effects of each SNP on daily alcohol intake and drinking status between the GG and GA genotypes of rs671. In the interaction analysis for daily alcohol intake, the linear regression models were fit as

$$\begin{array}{c} {\text{log}}_{2}\left(y+1\right)=\beta_{0}+\beta_{\text{rs}671}x_{\text{rs}671}+\\ \beta_{\text{SNP}}x_{\text{SNP}}+\beta_{\text{interaction}}x_{\text{rs}671}x_{\text{SNP}}+\sum_{k}\beta_{k}c_{k}+\varepsilon \end{array}$$

where *y* is daily alcohol intake (grams/day). *x*_rs671_ is the genotype of rs671. The GG genotype is coded as 0, and the GA genotype is coded as 1. Carriers of the AA genotype were excluded from the analysis. *x*_SNP_ is the imputed genotype coded as [0,2] for each SNP. *c_k_* is a covariate composed of age, age^2^, sex, the first 10 principal components, and 47 disease affection statuses (for the BBJ Study). ε is the error term. The effect sizes of the interaction term, β_interaction_, and its SEs estimated in the association analysis were used in the subsequent meta-analysis. In the interaction analysis for drinking status, the logistic regression model was fit as

$$\begin{array}{c} \text{ln}\left(\frac{p_{\text{ever}}}{1-p_{\text{ever}}}\right)=\beta_{0}+\beta_{\text{rs}671}x_{\text{rs}671}+\beta_{\text{SNP}}x_{\text{SNP}}+\\ \beta_{\text{interaction}}x_{\text{rs}671}x_{\text{SNP}}+\sum_{k}\beta_{k}c_{k} \end{array}$$

where *p*_ever_ is the probability that the subject is an ever drinker. Other variables and procedures are as above. The association analysis, including the interaction term, was conducted using PLINK2 (*74*).

In this study, we used rs671 genotypes directly extracted from SNP genotyping data, and no imputed data were used. With respect to concerns regarding genotype error, we further genotyped rs671 using TaqMan assays with the 7500 Real-Time PCR System (Applied Biosystems, Foster City, CA, USA) in all HERPACC samples in this study (*n* = 4958). Results confirmed a 99.96% (*n* = 4956) match of rs671 genotypes between the SNP microarray- and TaqMan-based data. The BBJ Study, the biggest data source in this study, also guaranteed a 100% concordance of rs671 genotyping between the SNP microarray and their in-house whole-genome sequencing data (*n* = 2798) in their previous study (*14*). All the other cohorts, accounting for 20% of the data in this study, also used the Illumina genotyping platform (table S2), indicating that we can be assured of the accuracy of rs671 genotypes in these studies.

To identify studies with inflated GWAS significance, which can result from population stratification, we computed the intercept from LDSC (*33*). Before the meta-analysis, all study-specific results in the association analysis were corrected by multiplying the SE of the effect size by the value of intercept from LDSC if the intercept of that study was greater than 1.

### Meta-analysis

The meta-analysis was performed with all Japanese subjects in the six cohorts (table S1). The results of association analyses for each SNP across the studies were combined with METAL software (*75*) by the fixed-effects inverse variance–weighted method. Heterogeneity of effect sizes was assessed by *I*^2^ and Cochran’s *Q* statistic. The meta-analysis included SNPs for which genotype data were available from at least three studies with a total sample size of at least 20,000 individuals for unstratified GWAS or interaction GWAS or 10,000 individuals for rs671-stratified GWAS. The genome-wide significance level α was set to a *P* value of <5 × 10^−8^. *P* values with <1.0 × 10^−300^ were calculated with Rmpfr of the R package. To assess the inflation of the test statistics for the meta-analysis, we computed the genomic inflation factor, λ, and intercept from LDSC (*76*).

### Joint meta-analysis

We used the JMA approach (*37*, *77*). The JMA jointly tests both SNP main effects β_SNP_ and SNP × rs671 interaction effects β_interaction_ for spherical equivalent with a fixed-effects model, using β_SNP_ and β_interaction_ and a β’s covariance matrix from each study. To perform the JMA, the same model as the interaction analysis for each study described above was analyzed using GEM v1.4 (*78*), which is capable of obtaining robust covariance matrices for β_SNP_ and β_interaction_. To control false positives, only SNPs with MAF ≥ 0.05 were analyzed by the GEM for each study.

The JMA was conducted with the fixed-effects method using METAL software (version 2010-02-08) (*75*) and patch source code provided by Manning *et al.* (*37*). A Wald’s statistic, following a χ^2^ distribution with two degrees of freedom (df), was used to test the joint significance of β_SNP_ and β_interaction_. A Cochran’s *Q* test was used to assess the heterogeneity of the β coefficients across studies for β_SNP_ and β_interaction_. The *cor* value was calculated by *cor* = IntCov/(StdErr × IntStdErr). IntCov is the covariance between β_SNP_ and β_interaction_ estimated by the JMA. StdErr and IntStdErr are SEs of β_SNP_ and β_interaction_ estimated by the JMA, respectively. The JMA included SNPs for which genotype data were available from at least three studies with a total sample size of at least 20,000 individuals for interaction GWAS. To control false positives, SNPs with evidence of between-study heterogeneity (Het*P* < 0.001) and *cor* < 0.7 were excluded (fig. S7). Genomic control correction was applied by calculating λ as the ratio of the observed and expected (2 df) median χ^2^ statistics and dividing the observed χ^2^ statistics by λ. The genome-wide significance level α for the JMA test was set to a *P* value of <5 × 10^−8^.

### Detection of independent association signals using conditional analysis

To detect further independent association signals at each associated locus for daily alcohol intake in rs671 heterozygotes (GA), we performed approximate conditional analyses using GCTA-COJO (*79*) with the summary statistics of the GWAS meta-analysis and LD estimated from imputed genotype data from 14,088 samples from the J-MICC Study. The region of 12q24 was excluded from the conditional analysis. Independent signals at a conditional threshold of *P* < 5 × 10^−8^ were detected.

### Definition of significant locus, signal (within the same locus), and signal particularly at 12q23-24

We defined the significant locus as that satisfying a >1-Mb distance from the lead SNP and demonstrating a genome-wide significant association on GCTA-COJO analysis. Furthermore, we defined a significant signal as that satisfying ≤1-Mb distance from the lead SNP and demonstrating a genome-wide significant association in GCTA-COJO analysis, except 12q23-24 because of the very long LD in this region. For 12q23-24, we used the definition of an SNP showing a genome-wide significant association in stratified GWAS.

### Functional annotations

To investigate the function of the lead SNP identified in this study, we adopted a series of bioinformatic approaches to collate functional annotations. We first used ANNOVAR (*80*) to obtain an aggregate set of functional annotations—including gene locations and impacts of amino acid substitutions based on prediction tools, such as SIFT, PolyPhen-2, and CADD—for SNPs with *P* values of <5 × 10^−8^. We also explored eQTLs using the GTEx v8 database (*81*) with regard to the loci identified in this study. The significant criteria for eQTL were based on the GTEx project: Variants with a nominal *P* value below the gene-level threshold were regarded as significant. This threshold was determined by permutation tests in the GTEx project to keep the false discovery rate below 5%.

### Genotyping of rs79463616 and comparison with imputed genotype

*ALDH2* rs79463616 was further genotyped by Sanger sequencing in the selected 96 HERPACC samples, which were also genotyped by Illumina HumanCoreExome. We confirmed a 100% match between the imputed and direct genotype data within these samples.

### Haplotype estimation of SNPs at 12q24.12, 4q23, and 9p13.2

We estimated haplotypes from genotypes of rs79463616 and rs671 at 12q24.12, rs1229984 and rs13125415 at 4q23, and rs2228093 and rs3043 for 1KGP-JPT (*68*) (*n* = 104) samples. Haplotypes of rs79463616 and rs671 were also estimated using the HERPACC (*n* = 96) samples. The genotype of rs79463616 for the HERPACC samples was determined by the method described above, while that of rs671 for these samples was determined by the Illumina HumanCoreExome SNP array. Using 4623 noncancer samples in the HERPACC Study, we genotyped rs671 and rs56884502 by TaqMan Assays using the 7500 Real-Time PCR System (Applied Biosystems, Foster City, CA, USA) and estimated their haplotypes. Furthermore, we estimated haplotypes from genotypes of rs671 and 70 SNPs at 12q24.12 and rs2228093 and seven SNPs at 9p13.2, which showed a *P* value of <1 × 10^−30^ for daily alcohol intake in the rs671 heterozygotes (GA) for the 1KGP-JPT samples (*n* = 104). In addition, within 982 noncancer samples in the HERPACC Study, we estimated haplotypes of rs671 and the six rs79463616 proxy SNPs of rs56884502, rs4648328, rs440, rs441, rs4646777, and rs4646778, which were genotyped using SNPtype assays with JUNO and EP1 System (Fluidigm, San Francisco, CA, USA). Haplotype estimation was performed using the Haploview software (*82*).

### Estimation of heritability and genetic correlation for the Japanese population

We estimated the SNP-based heritability of daily alcohol intake and drinking status for our Japanese meta-analysis with the use of LDSC (*33*). The heritability estimate was calculated from the summary statistics of high-quality common SNPs present in the HapMap 3 reference panel for the rs671 GG and GA analyses using East Asian LD scores. Genetic correlations among rs671 = GG and GA analyses were computed using bivariate LDSC and tested under the null hypothesis of *r*_g_ = 1.

### Estimation of cross-ethnic genetic correlation

We computed the cross-ethnic genetic correlation between Japanese and European populations. Cross-ethnic genetic correlations of both genetic effect and genetic impact were calculated using Popcorn (*40*). The GSCAN consortium’s summary statistics for drinks per week (*12*) was used for the European population. For the Japanese population, log_2_ (grams/day + 1) summary statistics were used for the rs671 = GG and rs671 = GA populations, respectively.

### Esophageal cancer case-control study

#### Study sample

In the HERPACC Study, we included 692 cases and 995 age- and sex-matched controls who were selected from participants in HERPACC-2 (2001–2005) (*83*) and HERPACC-3 (2005–2013) (*24*). Cases were first-visit outpatients at Aichi Cancer Center Hospital who were diagnosed with esophageal cancer within −3 to +12 months of the first visit. Controls were first-visit outpatients who were confirmed to have no cancer or history of neoplasm. The BBJ Study included 416 cases and 86,515 controls after excluding (i) outliers from the Japanese cluster, as estimated by principal components analysis with samples of the 1000 Genomes project (*68*), and (ii) closely related individuals estimated by King (*84*) (specifically, King kinship coefficients > 0.09375). Cases were diagnosed with esophageal cancer within −3 to +12 months from the date of consent. Controls were those confirmed to have no cancer or history of neoplasm. In the HERPACC Study, esophageal cancer cases were identified using the International Classification of Diseases for Oncology, Third Edition (ICD-O-3) (*85*) topography code C15. As a sensitivity analysis, we performed an additional analysis restricted to cases with squamous cell carcinoma identified using the ICD-O-3 morphology codes of 8050–8078 and 8083–8084, resulting in 636 cases. In the BBJ Study, all participants had been diagnosed with at least one of 47 target diseases, including esophageal cancer, by physicians at the cooperating hospitals. Esophageal cancer histology was determined from excised tissue specimens, and missing histological data were complemented by cytological specimens, resulting in 348 cases of squamous cell carcinoma.

#### Genotyping and imputation procedure

In the HERPACC Study, genomic DNA was extracted from peripheral blood using a DNA Blood mini kit (Qiagen, Tokyo, Japan), and eight SNPs were genotyped using TaqMan Assays with the 7500 Real-Time PCR System (Applied Biosystems, Foster City, CA, USA) or SNPtype assays with JUNO and EP1 System (Fluidigm, San Francisco, CA, USA). We confirmed a 100% match between rs4648328 and rs79463616 genotypes in the 96 selected HERPACC samples using Sanger sequencing. The genotyping and imputation procedure in the BBJ Study is described in the “Details of studies” section of the Supplementary Materials.

#### Statistical analysis

ORs for esophageal cancer per 1-allele change in eight SNPs were estimated on three different subject groups (entire population, subjects with the rs671 GG genotype only, and subjects with the rs671 GA genotype only) using a logistic regression model adjusted for sex, age, the first 10 principal components (for the BBJ Study), and the study version (for the HERPACC Study). Study-specific ORs were then pooled using a random-effects model (*86*). We evaluated the extent of between-study heterogeneity by the Cochran *Q* statistic and I^2^ statistic (*87*).

The interaction between rs671 and each of the variants under study was evaluated on both additive and multiplicative scales. Carriers of the rs671 AA genotype were excluded from this analysis. First, we estimated the study-specific coefficients of each SNP (per 1-allele change), rs671 (GA versus GG), and the product term of each SNP and rs671 using the following logistic regression model

$$\begin{array}{c} \text{ln}\left(\frac{p}{1-p}\right)=\beta_{0}+\beta_{\text{SNP}}x_{\text{SNP}}+\beta_{\text{rs}671}x_{\text{rs}671}+\\ \beta_{\text{Int}}x_{\text{SNP}}x_{\text{rs}671}+\sum_{k}\beta_{k}c_{k} \end{array}$$

where *p* is the probability that the subject is a case of esophageal cancer, *x*_SNP_ is the SNP genotype, *x*_rs671_ is the genotype of rs671, and *c_k_* is each of the adjusting variables. For the HERPACC Study, *x*_SNP_ was coded as follows: The Ref/Ref genotype was coded as 0, the Ref/Alt genotype as 1, and the Alt/Alt genotype as 2. For the BBJ Study, *x*_SNP_ was the imputed genotype coded as [0,2] for each SNP. For the rs671 phenotype, the GG genotype was coded as 0, and the GA genotype was coded as 1. We then obtained pooled estimates $\bar{\beta_{\mathrm{SNP}}}$ , $\bar{\beta_{\text{rs}671}}$ , and $\bar{\beta_{\text{Int}}}$ of the model coefficients corresponding respectively to the effect of the SNP, rs671, and their interaction term (the product term for each SNP and rs671) using multivariate meta-analysis (*88*) to account for the fact that coefficients estimated from the same study are correlated. Specifically, we conducted random-effects multivariate analyses based on likelihood maximization using the study-specific β_SNP_, β_rs671_, and β_Int_ coefficients as well as their covariance matrix. Multiplicative interaction was measured by the summary OR associated with the interaction term, i.e., $e^{\bar{\beta_{\text{Int}}}}$ with its corresponding 95% confidence interval. As the measure of additive interaction, we estimated the relative excess risk due to interaction (RERI) (*89*) by the following formula

$$\text{RERI}=e^{\bar{\beta_{\text{SNP}}}+\bar{\beta_{\text{rs}671}}+\bar{\beta_{\text{Int}}}}-e^{\bar{\beta_{\text{SNP}}}}-e^{\bar{\beta_{\text{rs}671}}}+1$$

where $e^{\bar{\beta_{\text{SNP}}}+\bar{\beta_{\text{rs}671}}+\bar{\beta_{\text{Int}}}}$ , $e^{\bar{\beta_{\text{SNP}}}}$ , and $e^{\bar{\beta_{\text{rs}671}}}$ are summary estimates of the joint OR for each SNP and rs671, OR for each SNP, and OR for rs671, respectively. Confidence intervals for RERI were estimated by the Delta method (*90*), and *P* values were based on the Wald test. Statistical significance was set at the Bonferroni-corrected threshold of *P* < 0.05/8 (=0.00625), and suggestive significance was set at *P* < 0.05. RERI was considered to achieve suggestive significance (*P* < 0.05) when its confidence interval did not include 0. Analyses were performed with R version 4.1.2 (The R Foundation for Statistical Computing) or STATA version 17.0 (Stata Corporation, College Station, TX, USA).

### PRS analyses

#### Study subjects for PRS

The PRS subjects were from the J-MICC Study, as described in the “Details of studies” section in the Supplementary Materials. We selected 28,658 subjects for this genotyping out of 58,925 eligible participants who were not previously genotyped and had been confirmed to be noncancerous up until 2017, considering the area distribution of respondents. Therefore, these PRS subjects do not overlap with those included in the GWAS meta-analysis. A total of 28,643 DNA samples were successfully genotyped by Japonica Array NEO (*91*). These samples were divided into six batches for genotyping, QC, and imputation. Details of those processes have been described in previous studies (*92*). The number of SNPs used for imputation in each batch is listed in table S2. After imputation, these batches were merged. After the post-imputation QC of samples, 52 non-Japanese samples based on principal components analysis of samples with the 1000 Genomes Project phase 3 and 1720 related samples based on PLINK --genome option (PI-HAT ≥ 0.1875) were excluded. Accordingly, 26,871 participants remained for further processing. Participants with a history of cancer, no information on alcohol consumption, or missing rs671 genotype data were excluded. Last, 24,612 participants remained for further analysis (tables S1 and S3). Among 24,612 participants, 13,686 rs671 wild-type homozygotes (GG), 9201 heterozygotes (GA) and 1725 variant homozygotes (AA) were directly determined by Japonica Array. As a post-imputation QC of SNPs, variants with info < 0.9 or MAF < 0.01 were excluded, resulting in 8,819,485 SNPs for further analysis. The imputed genotype data were converted to best-guess genotypes.

#### Construction and validation of PRS for daily alcohol intake

We constructed PRSs using each of the unstratified, rs671 wild-type homozygotes (GG)–only, and rs671 heterozygotes (GA)–only GWAS summary statistics based on imputed data of the J-MICC Study as measured by the Japonica Array described above. First, a linear association analysis was conducted on the 24,612 individuals with log_2_ (daily alcohol intake + 1) as the dependent variable and age, age^2^, sex, and top 10 principal components as independent variables. The residuals obtained were adopted as the phenotype for PRS. We applied the split-sample approach to construct and validate the PRS. The total eligible 24,612 individuals, all with direct genotype data of rs671, were randomly assigned to either the target or validation datasets in a 2:1 ratio (tables S3 and S21). Consequently, 16,408 individuals were allocated to the target dataset and the remaining 8204 to the validation dataset. When we divided the groups according to rs671 genotype, the target dataset included 9124 wild-type homozygotes and 6134 heterozygotes, and the validation dataset included 4562 wild-type homozygotes and 3067 heterozygotes.

The scheme of this PRS analysis is shown in fig. S16. We started by developing three types of PRS, each made by combining different summary statistics and corresponding target datasets. The first PRS, termed PRS_unstratified_, was created using the summary statistics of the unstratified GWAS and was applied to a target dataset of 16,408 individuals. Next, we created PRS_GG_, which used the summary statistics of the rs671 GG-only GWAS. This was applied to a corresponding target dataset consisting of 9124 wild-type homozygotes. Last, we created PRS_GA_, using the summary statistics of the rs671 GA-only GWAS, and applied it to a target dataset consisting of 6134 heterozygotes. In the summary statistics, *P* values of <1.0 × 10^−300^ were converted to *P* = 1.0 × 10^−300^. The residuals of log_2_ (daily alcohol intake + 1), calculated as described in the previous section, were adopted as phenotypes. Genotype data for PRS calculations were converted from the imputed genotypes to best-guess genotypes. For the PRS calculations, PRSice-2 software (*93*) was used. The PRSice-2 constructs PRS by the clumping and thresholding (C + T) method. The default settings were applied for the PRS construction. Briefly, LD clumping was performed with the options “--clump-kb 250kb --clump-p 1.000000 --clump-r2 0.100000.” *P* value thresholding was then performed with the options “--lower 5e-08 --upper 0.5 --interval 5e-05.” The ambiguous SNPs were not excluded with the option “--keep-ambig.” That is, an additive model was used that sums the number of effect alleles an individual has (either 0, 1, or 2). In the PRS construction, the PRS with the best performance in the target dataset was constructed based on the summary statistics of each GWAS meta-analysis. Predictive performance was assessed by R^2^. This value represents the proportion of variance of the phenotype, residuals of log_2_ (daily alcohol intake + 1), explained by the PRS. The PRS for each participant was calculated as a standardized PRS. For example, the PRS_GG,*i*_ for participant *i* was calculated as shown in the following equation

$${\text{PRS}}_{\mathrm{GG},i}=\frac{\sum_{j}\beta_{j}G_{\mathrm{ij}}-m_{\mathrm{GG}}}{s_{\mathrm{GG}}}$$

where β*_j_* is the effect size for SNP *j* and *G_ij_* is the number of effect alleles for the genotype for SNP *j* in participant *i*, *m*_GG_ is a mean value of $\sum_{j}\beta_{j}G_{\mathrm{ij}}$ across the target dataset composed of wild-type homozygotes, and *s*_GG_ is an SD value of $\sum_{j}\beta_{j}G_{\mathrm{ij}}$ across the same target dataset. These mean and SD values for each PRS are shown in table S21. To assess how well PRS_unstratified_, PRS_GG_, and PRS_GA_ predict residuals of log_2_ (daily alcohol intake + 1) on the validation dataset, we calculated the PRS for each of 8204 individuals, 4562 wild-type homozygotes, and 3067 heterozygotes, respectively, in the validation dataset.

#### Comparison of rs671 and PRS_unstratified_ in individuals with either the rs671 GG, GA, or AA genotypes

We compared the predictive performances of rs671 alone and PRS_unstratified_. To assess their predictive performance as R^2^, 8204 individuals with either the rs671 GG, GA, or AA genotypes were used as the validation dataset (table S3). To calculate each R^2^ value, a linear association analysis was conducted with the residual of log_2_ (daily alcohol intake + 1) as the dependent variable and rs671 genotype (GG = 0, GA = 1, AA = 2) or PRS_unstratified_ as the independent variable.

#### Comparison of rs671, PRS_unstratified_, and combined score in individuals with either the rs671 GG or GA genotypes

We compared the predictive performances of rs671 alone; PRS_unstratified_; and a combined score of rs671, PRS_GG_, and PRS_GA_. To assess their predictive performance as R^2^, 7629 individuals with either the rs671 GG or GA genotypes were used as the validation dataset (table S3). To calculate each R^2^ value, a linear association analysis was conducted with the residual of log_2_ (daily alcohol intake + 1) as the dependent variable and rs671 genotype (GG = 0, GA = 1), PRS_unstratified_, or the combined score as the independent variable. The combined score for participant *i* follows the formula shown in the following equation

$$\begin{array}{c} {\text{Combined score}}_{i}=w_{0}+w_{1}\times G_{i,\text{rs}671}+w_{2}\times \left(1-G_{i,\text{rs}671}\right)\times {\text{PRS}}_{\text{GG},i}+\\ w_{3}\times G_{i,\text{rs}671}\times {\text{PRS}}_{\text{GA},i} \end{array}$$

where *G*_*i*,rs671_ is the genotype of rs671 for participant *i* (GG = 0, GA = 1). *w*_0_, *w*_1_, *w*_2_, and *w*_3_ are weights, which were obtained by linear regression analysis using the target dataset of individuals with GG or GA genotypes

$$\begin{array}{c} y_{\text{res},i}=w_{0}+w_{1}\times G_{i,\text{rs}671}+w_{2}\times \left(1-G_{i,\text{rs}671}\right)\times {\mathrm{PRS}}_{\mathrm{GG},i}+\\ w_{3}\times G_{i,\text{rs}671}\times {\mathrm{PRS}}_{\mathrm{GA},i}+\varepsilon_{\text{res},i}, \end{array}$$

where *y*_res,*i*_ is the residual of log_2_ (daily alcohol intake + 1) for participant *i*. *w*_0_, *w*_1_, *w*_2_, and *w*_3_ are regression coefficients. ε_res,*i*_ is the error term. The weights obtained are shown in table S23.

### Ethics statement

All studies were approved by their respective institutional review boards.

## Supplementary Materials

### This PDF file includes:

Supplementary Methods Figs. S1 to S19 Legends for tables S1 to S25 References
