# Supplementary material for: Genetic architecture of alcohol consumption identified by a genotype-stratified GWAS and impact on esophageal cancer risk in Japanese people
Source: Sci Adv. 2024 Jan 26;10(4):eade2780. doi: 10.1126/sciadv.ade2780 (PMC10816704; doi:10.1126/sciadv.ade2780)
Supplement: Supplementary file 1 — Supplementary Methods Figs. S1 to S19 Legends for tables S1 to S25 References [file sciadv.ade2780_sm.pdf]

Science Advances
AAAS

Supplementary Materials for

Genetic architecture of alcohol consumption identified by a genotype-stratified GWAS and impact on esophageal cancer risk in Japanese people

Yuriko N. Koyanagi *et al.*

Corresponding author: Keitaro Matsuo, kmatsuo@aichi-cc.jp; Yuriko N. Koyanagi, ykoyanagi@aichi-cc.jp; Masahiro Nakatochi, mnakatochi@met.nagoya-u.ac.jp

Sci. Adv. **10**, eade2780 (2024)
DOI: 10.1126/sciadv.ade2780

The PDF file includes:

Supplementary Methods
Figs. S1 to S19
Legends for tables S1 to S25
References

Other Supplementary Material for this manuscript includes the following:

Tables S1 to S25

Details of studies

Six studies with a total of 175,672 Japanese individuals with no history of cancer were included in the analysis of the present study. The individual study descriptions are as follows:

Hospital-based Epidemiologic Research Program at Aichi Cancer Center (HERPACC) Study

The HERPACC-2 Study was conducted between January 2001 and November 2005 at Aichi Cancer Center. Details of the HERPACC Study are described elsewhere (*25*). Briefly, every first-visit outpatient (*n* = 29,736) was asked to fill out a self-administered questionnaire regarding their lifestyle, including information on alcohol consumption, and provide a blood sample. 96.7% of eligible subjects (*n* = 28,776) participated in the study with written informed consent and completed the questionnaire. 48.0% of participants (*n* = 13,824) further provided 7mL of blood. Of these, 7,053 subjects were confirmed to have no detectable cancer and no history of neoplasia within a one-year window period from participation. For the present study, 4,958 randomly selected non-cancer subjects (genotyped using Illumina Human610-Quad [*n* = 1,147] or Illumina HumanCoreExome [*n* = 3,811]) were included in the final analysis, after quality control and exclusion of those who did not answer questions about alcohol consumption.

The HERPACC Study was conducted in accordance with the Declaration of Helsinki and approved by the Institutional Ethics Committee of Aichi Cancer Center, Nagoya, Japan.

Japan Multi-Institutional Collaborative Cohort (J-MICC) Study

The baseline survey of the J-MICC Study was conducted between 2004 and 2014 across 13 study sites in Japan. Details of the J-MICC Study are described elsewhere (*26, 27*). We selected 14,555 eligible participants for GWAS, considering the area distribution of respondents and the minimum sample size (*n* = 500) for one study site. A total of 14,536 DNA samples were successfully genotyped for GWAS. After quality control, 14,091 participants remained for the imputation process. Participants who had a history of cancer or did not answer questions about alcohol drinking were excluded. Finally, 13,236 participants remained for the association analysis.

The J-MICC Study was approved by the ethics committee of Nagoya University Graduate School of Medicine (Approval No.: 2010-0939), Nagoya, Japan, and written informed consent was obtained from all the participants in the present study.

Japan Public Health Center-based Prospective (JPHC) Study

The JPHC Study was commenced in 1990 (cohort I) and 1993 (cohort II). Participants were recruited from 11 public health center areas in Japan. At the time of the baseline survey, the subjects were aged between 40-59 years in 1990 (cohort I) and 40-69 years in 1993 (cohort II). Details of the JPHC Study are described elsewhere (*28*). From participants who answered the baseline survey questionnaire and donated 10mL blood in 9 public health center areas (*n* = 33,736), we randomly selected 13,024 participants for genotyping from respective strata divided by sex, 5-year age category and public health center area. Subsequently, we performed the standard quality control for GWAS, leaving 10,295 participants for this analysis. After excluding participants who had a past history of cancer or did not completely answer questions about alcohol drinking, 10,037 participants (3,462 men and 6,575 women) were included in the analysis of

drinking frequency and 9,142 participants (3,096 men and 6,046 women) were included in the analysis of drinking quantity.

Before the performance of genetic research on the JPHC samples, the study protocol was approved by the institutional review board of the National Cancer Centre (Approval No.:2011-044), Tokyo, Japan. Participants had the option to refuse participation in the research.

The Tohoku Medical Megabank Community-Based Cohort (TMM CommCohort) Study

The TMMCC Study (*29*) is a population-based cohort study which is part of the TMM Project (*94*), launched for the purpose of reconstruction from the Great East Japan Earthquake and establishment of personalized healthcare and medicine. The study recruited more than 80,000 residents aged 20 years or older living in Iwate and Miyagi Prefectures, located on the Pacific side of the Tohoku (northeastern) region of Honshu (the main island of Japan), from May 2013 to March 2016.

As a pre-analysis quality control, we filtered a data set of 9,965 participants (3,481 males and 6,484 females) who participated in the TMM CommCohort Study at health check-up venues in 2013, based on the following criteria: 1) call rate < 0.98, 2) inconsistent self-reported and genotype-based sex information, 3) close relatives (PI_HAT > 0.1875 = 3/16), 4) no available records for drinking-related items, height, or weight, 5) BMI (kg/m^2) < 14 or > 45, and 6) history of cancer. After filtering, 7,857 participants (2,691 males and 5,166 females) were retained for the current analysis.

The TMM CommCohort Study was approved by the Institutional Review Boards of Iwate Medical University and Tohoku University, and all the participants gave written informed consent. The study was conducted in accordance with the Declaration of Helsinki.

The Nagahama Prospective Cohort for Comprehensive Human Bioscience (Nagahama) Study

The Nagahama Study is a community-based cohort study conducted in Shiga, Japan (*30*). Participants with no physical impairment or dysfunction were recruited from the general population aged 30 – 74 years in Nagahama City from 2008 to 2010. Of the 9,757 eligible participants, 5,730 randomly selected subjects who participated mainly during 2008 and 2009 were genotyped using illumine SNP array. We then obtained 4,591 subjects (Illumina Human610-Quad [*n* =1,170], Illumina Omni2.5 [*n* = 777], Illumina Omni5M [*n* = 472], Illumina Infinium HumanCoreExome [*n* =967] or Illumina Infinium Asian Screening Array [*n* =1,205]) by filtering with the following criteria: 1) call rate <0.99, 2) close relatives (PI_HAT > 0.38), and 3) history of cancer according to self-administrated questionnaire.

The Nagahama Study was approved by the ethics committee of Kyoto University Graduate School of Medicine and by Nagahama Municipal Review Board. All participants gave written informed consent.

The BioBank Japan (BBJ) Study

The BBJ Study is a hospital-based biobank that collected DNA, serum, and clinical information of approximately 200,000 participants from 12 medical institutions in Japan (*31, 32*). All participants had been diagnosed with at least one of 47 target diseases by physicians at the cooperating hospitals. Written informed consent was obtained from all participants, as approved by the ethics committee of the Institute of Medical Science, the University of Tokyo.

We genotyped participants with the Illumina HumanOmniExpress Exome-8 platform or a combination of the Illumina HumanOmniExpress-12v1 and HumanExome-12 platforms. Quality control of participants, genotyping, and imputation were performed as described elsewhere (*95*). In this project, we excluded participants meeting the following criteria: (1) outliers from the Japanese cluster as estimated by principal component analysis with samples of the 1000 Genomes project (*68*), (2) closely related individuals as estimated by King (*84*) (specifically, King kinship coefficients > 0.09375), (3) individuals with any cancer diagnosis, and (4) individuals whose drinking phenotype was missing. We also excluded former drinkers from the GWAS of daily alcohol intake. Finally, we included 134,993 and 115,493 participants in the GWAS of drinking status and daily alcohol intake, respectively.

Phenotype

Information on alcohol consumption was collected via a questionnaire in each study. Because the questionnaires were not homogeneous across the studies, we harmonized the two alcohol consumption phenotypes of drinking status (never versus ever drinker) and daily alcohol intake (g/day) in accordance with each study's criterion, as follows:

The HERPACC Study

In the questionnaire related to alcohol consumption in the HERPACC-2 Study, the participants were first asked about drinking status, as "never" (including "almost never"), "former", or "current". Participants who selected "former" or "current" were defined as ever drinkers. Former and current drinkers were then asked about the frequency of alcohol drinking (<1 day/week, 1–2 days/week, 3–4 days/week, or ≥5 days/week), along with type of beverage (Japanese sake, beer, shochu, whiskey, and wine) and average consumption during each drinking session based on their average drinking behavior. To calculate daily alcohol intake (g/day), each category of drinking frequency was assigned a score as follows: 0 for never drinkers, 0.5 for <1 day/week, 1.5 for 1–2 days/week, 3.5 for 3–4 days/week, and 6 for ≥5 days/week. Alcohol intake per drinking session was estimated based on the concentration of ethanol in each beverage. In the HERPACC Study, one unit of drink was assumed to contain 23 g of ethanol for 180 ml (one "go") of Japanese sake, 633 ml (one large bottle) of beer, 90 ml (a half "go") of shochu (distilled spirit), 60 ml (double shot) of whiskey, and 200 ml (two and a half glasses) of wine. We therefore estimated alcohol intake per drinking session by multiplying the summed units of each type of beverage by 23. Finally, daily alcohol intake was estimated by multiplying alcohol intake per drinking session by the frequency score/7.

The J-MICC Study

All of the study sites used a common, standard self-administered questionnaire. The participants were first asked about drinking status (almost none, former, or current). Those who selected "almost none" were defined as never drinkers and the others were defined as ever drinkers. Current drinkers were further required to report the frequency (almost none, 1 to 3 days/month, 1 to 2 days/week, 3 to 4 days/week, 5 to 6 days/week, or everyday) and amount of alcohol consumption for six alcoholic beverages (Japanese sake, shochu, shochu-based highball, beer, whisky, and wine). Daily alcohol intake was estimated for current

drinkers based on the frequencies and amount of each type of alcoholic beverage consumed over the past year.

The JPHC Study

Information on alcohol intake was obtained regarding frequency and quantity using a validated questionnaire at baseline survey. The average frequency of alcohol intake was reported according to six categories for cohort I: “almost never”, “1–3 days per month”, “1–2 days per week”, “3–4 days per week”, “5–6 days per week”, and “every day”. Participants who selected “almost never” were defined as never drinkers and others were defined as ever drinkers. Type of alcohol and the average amount were also ascertained. Participants in cohort II were asked to indicate their alcohol drinking status as “never”, “past”, or “current drinker”. Past and current drinkers provided information on the average frequency of intake, the type of alcohol consumed, and the average quantity consumed per day. To calculate the total alcohol consumption per day, we added the quantity of ethanol of each alcoholic beverage. The amount of ethanol was assigned as follows: 180 ml of sake (rice wine) was regarded as 23 g of ethanol, 180 ml of shochu or awamori (white spirits) as 36 g, 633 ml of beer as 23 g, 30 ml of whiskey or brandy as 10 g, and 60 ml of wine as 6 g. This calculation was validated in the previous paper (*96*).

The TMMCC Study

Drinking status for each participant was assigned based on a question on drinking habit in a self-administered questionnaire used in the cohort. The question has four options: “current drinker”, “former drinker”, “(almost) non-drinker”, and “alcohol intolerance”. Both “(almost) non-drinker” and “alcohol intolerance” were classified as “never drinker”; the others were classified as “ever drinker”. The participants were also asked to answer a pair of questions, one on drinking frequency and the other on amount per event, for each type of alcoholic beverage. Alcohol intake was calculated for 7,627 participants (3,634 current drinkers and 3,993 never-drinkers), with 230 former drinkers excluded. The question on drinking frequency had six options: “almost none”, “1-3 days/month”, “1-2 days/week”, “3-4 days/week”, “5-6 days/week”, and “every day.” The answers were converted into the number of events per day, i.e., 0, 2/30, 1.5/7, 1/2, 5.5/7, and 1, respectively. The amount per event was converted into the ethanol content (in grams) as follows: 180 ml of sake (rice wine) as 23 g, 180 ml of shochu (distilled spirits) as 36 g, 180 ml of chuhai (shochu-based beverage) as 12.96 g, 633 ml of beer as 23 g, 30 ml of whisky as 10 g, and 100 ml of wine as 12 g. The alcohol intake (g/day) for each participant was then calculated as the sum of the product of the ethanol content and the number of events per day for all types of alcoholic beverage. For never drinkers, alcohol intake was considered as zero.

The Nagahama Study

Information on the amount and frequency of alcohol consumption was collected through questionnaires. Participants were first asked to report their drinking status (never, almost never, former, sometimes, or every day). The participants who selected “never” or “almost never” were defined as never drinkers and the others were defined as ever drinkers. Those who selected “sometimes” or “everyday” were regarded as “current” drinkers, and were further required to report the frequency and amount of alcohol consumption for

six alcoholic beverages (Japanese sake, shochu, shochu-based highball, beer, whisky, and wine). Daily alcohol intake was estimated for current drinkers based on the consumption frequency and amount of each type of alcoholic beverage over the past year.

The BBJ Study

Drinking phenotypes were obtained by interviewing the participants using a standardized questionnaire. Participants were first asked about their drinking status (never or ever). Ever drinkers were then asked about their current drinking status (former or current). Finally, current drinkers were asked about the type, volume (mL), and frequency (per week) of consumption of alcoholic drinks. The daily alcohol intake (g/day) was calculated by multiplying the percentage of alcohol by the volume, frequency, and alcohol density (0.8 g/ml).

PheWAS

Considering that alcohol consumption is associated with various diseases and disorders, we performed PheWAS to assess the associations of the seven variants with diverse phenotypes other than esophageal cancer. This PheWAS was carried out within the BBJ Study targeting 122 phenotypes, including 46 diseases, 16 dietary and activity habit phenotypes, and 60 biomarkers (table S24). All diseases were diagnosed by physicians at the cooperating hospitals. The dietary and activity phenotypes were derived from a standardized self-reported questionnaire. The response choices in the questionnaire were converted to numeric values on the same scale as that employed in Yamamoto K *et al. (97).* Specifically, for dietary habits, we assigned values to the frequency of consumption, with almost every day = 7, 3–4 days per week = 3.5, 1–2 days per week = 1.5, and rarely = 0. For activity habits, participants were asked for the frequency and length of time per week of the activities on a seven-point scale, which we used to calculate the total minutes of activity time per week. The phenotype values of height and weight were also retrieved from the self-reported questionnaire. The other biomarkers were obtained from medical records of routine laboratory examinations, for which we performed quality control as described in Kanai *et al (52).* Briefly, we normalized phenotype values using Z-score or rank-based inverse normal transformation after correcting with biomarker-specific covariates. Analyses were conducted on three distinct groups of subjects, namely the entire population (unstratified), rs671 wild-type homozygotes (GG), and heterozygotes (GA); the interaction of each variant with rs671 on each phenotype was also evaluated. The association analysis, conducted using PLINK2, included the same covariates as those utilized in the examination of daily alcohol intake and drinking status. We set a *P*-value of $0.05/122 = 4.10\times10^{-3}$ as the significance threshold.

The results of this PheWAS are reported in fig. S19 and table S25. Six of the seven variants, *GCKR* rs1260326, *KLB* rs28712821, *ADH1B* rs1229984, *ALDH1A1* rs8187929, *ALDH2* rs79463616, and *GOT2* rs73550818, satisfied the significance threshold ($P < 0.05/122 = 4.10\times10^{-3}$) in the association with any of the phenotypes in any analysis. Among these, *GCKR* rs1260326, *KLB* rs28712821, *ADH1B* rs1229984, and *ALDH2* rs79463616 showed significant association with any of the phenotypes in the analysis of either rs671 GG- or GA-only, but not both, thereby implying that they interact with the rs671 genotype. Of these, *ADH1B* rs1229984 showed significant interaction with rs671 in the risk of myocardial infarction and

dyslipidemia. The association of *ADH1B* rs1229984 with myocardial infarction is of particular interest. In the unstratified analysis, rs1229984 was not associated with myocardial infarction, but after considering gene-gene interaction, rs1229984 had a preventive effect on myocardial infarction under the condition of being the rs671 GA genotype. This means that further risk stratification can be done by rs1229984 among the population with the rs671 A allele, a possible risk factor for myocardial infarction (*98, 99*). Given that moderate alcohol use may be associated with a reduction in the risk of myocardial infarction (*100*) and that rs1229984 was associated with an increased level of alcohol consumption in the rs671 heterozygotes, who are assumed to not drink heavily, we consider that this association may be reasonable. Furthermore, this association is likely supported or suggested by a similar trend in relation to dyslipidemia and total cholesterol (TC) and LDL cholesterol (LDL-C) levels (table S25). Taken together, although certain associations identified in the PheWAS posed difficulties in the identification of clear and logical connections, we successfully unveiled associations in the risk of certain traits with specific SNPs showing interaction with rs671 through the PheWAS upon stratification.

Fig. S1. Q-Q plots for rs671 genotype-stratified GWAS meta-analysis of daily alcohol intake

The results for (A) unstratified, (B) rs671 wild-type homozygotes (GG), (C) rs671 heterozygotes (GA), and (D) interaction with rs671 are shown. The vertical and horizontal axes indicate the observed and expected –$\log_{10}$(*P* value) for tests of association between SNPs and daily alcohol intake, respectively.

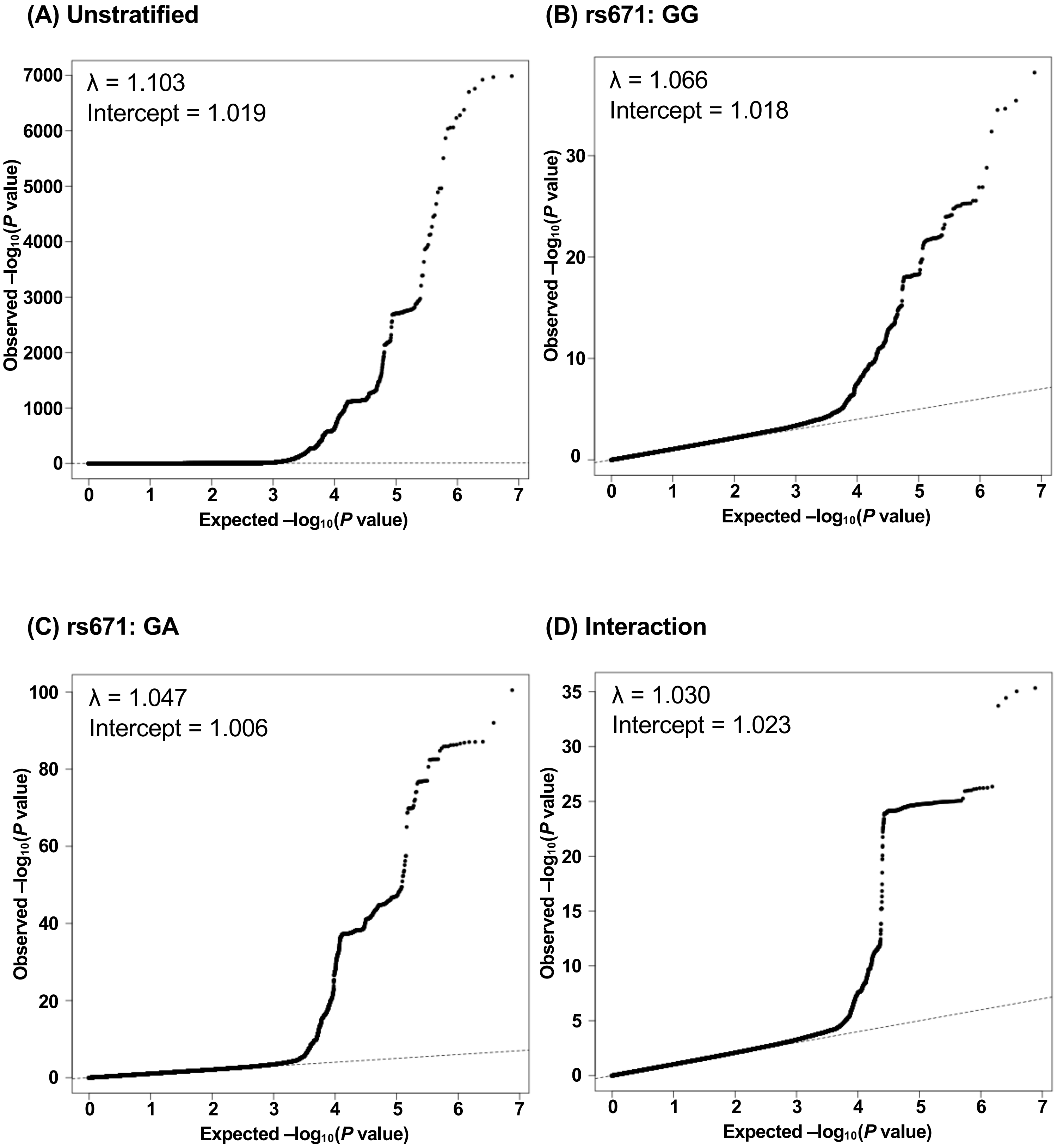

Fig. S2. Q-Q plots for rs671 genotype-stratified GWAS meta-analysis of drinking status

The results for (A) unstratified, (B) rs671 wild-type homozygotes (GG), (C) rs671 heterozygotes (GA), and (D) interaction with rs671 are shown. The vertical and horizontal axes indicate the observed and expected $-\log_{10}$(P value) for tests of association between SNPs and drinking status, respectively.

(A) Unstratified

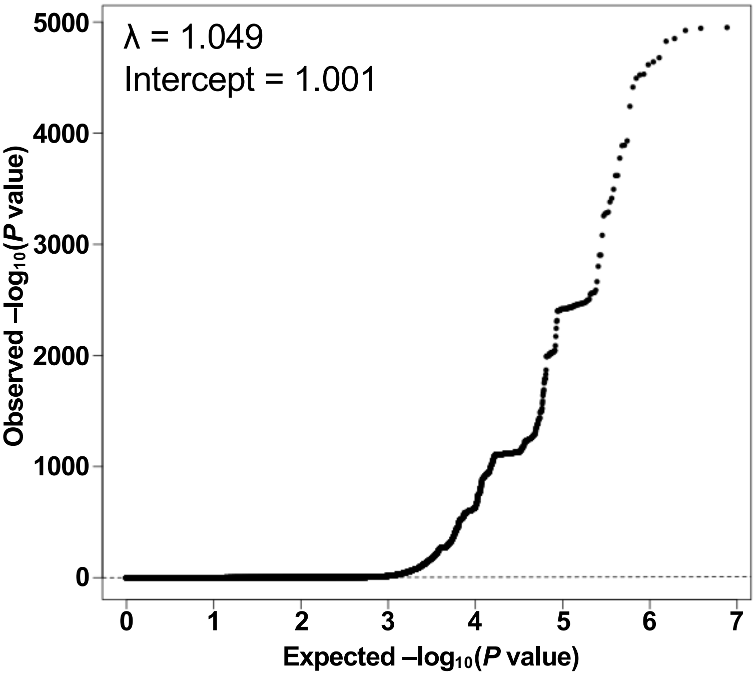

(B) rs671: GG

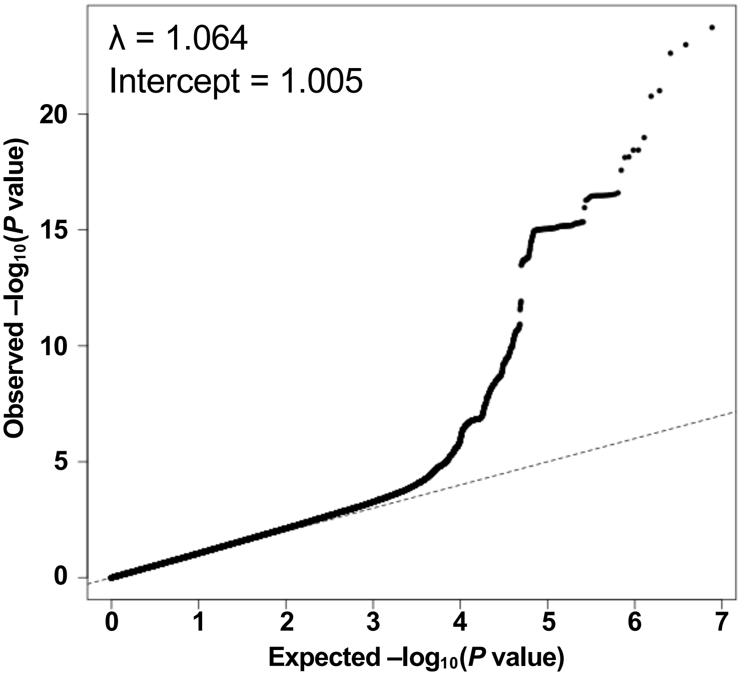

(C) rs671: GA

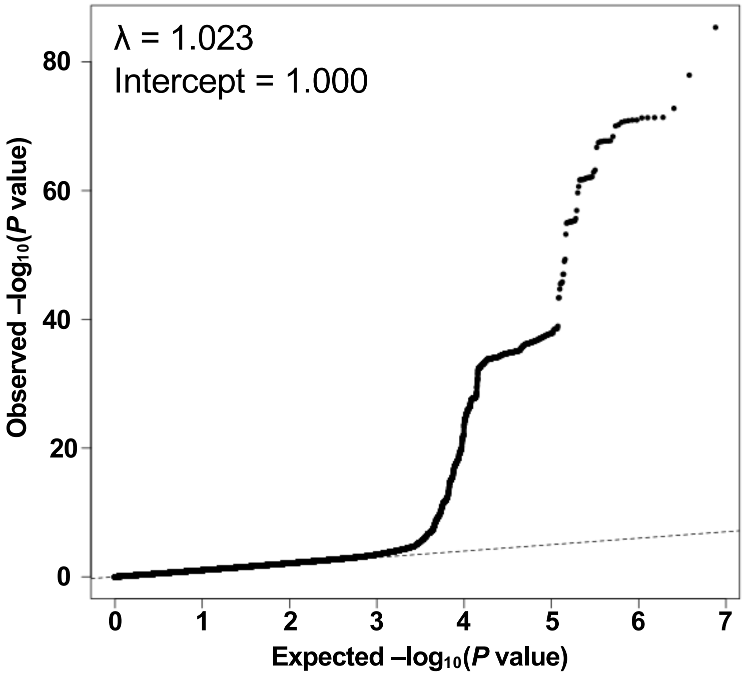

(D) Interaction

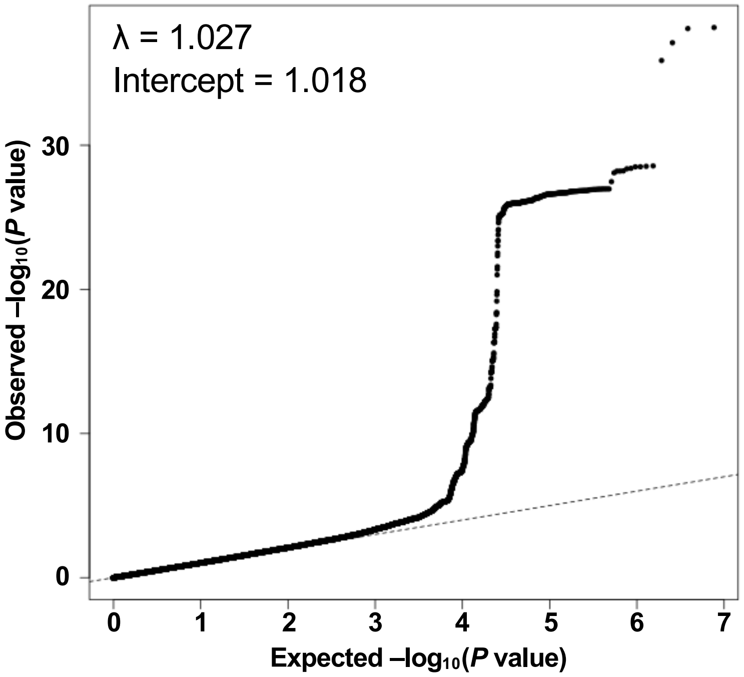

Fig. S3. Manhattan plots of the GWAS of drinking status

The results for (A) unstratified, (B) rs671 wild-type homozygotes (GG), (C) rs671 heterozygotes (GA), and (D) interaction with rs671 are shown. The position on each chromosome (x-axis) and the observed $-\log_{10}(P \text{ value})$ (y-axis) of all tested genetic variants are shown. The solid red line indicates genome-wide significance level. Blue triangles represent loci containing SNPs with *P* values of $<1 \times 10^{-50}$. **ADH1B P* value (C): 1.59×10^{-73}; ***ALDH2 P* value (C): 3.39×10^{-4952}; †*ADH1B P* value (B): 4.36×10^{-86}.

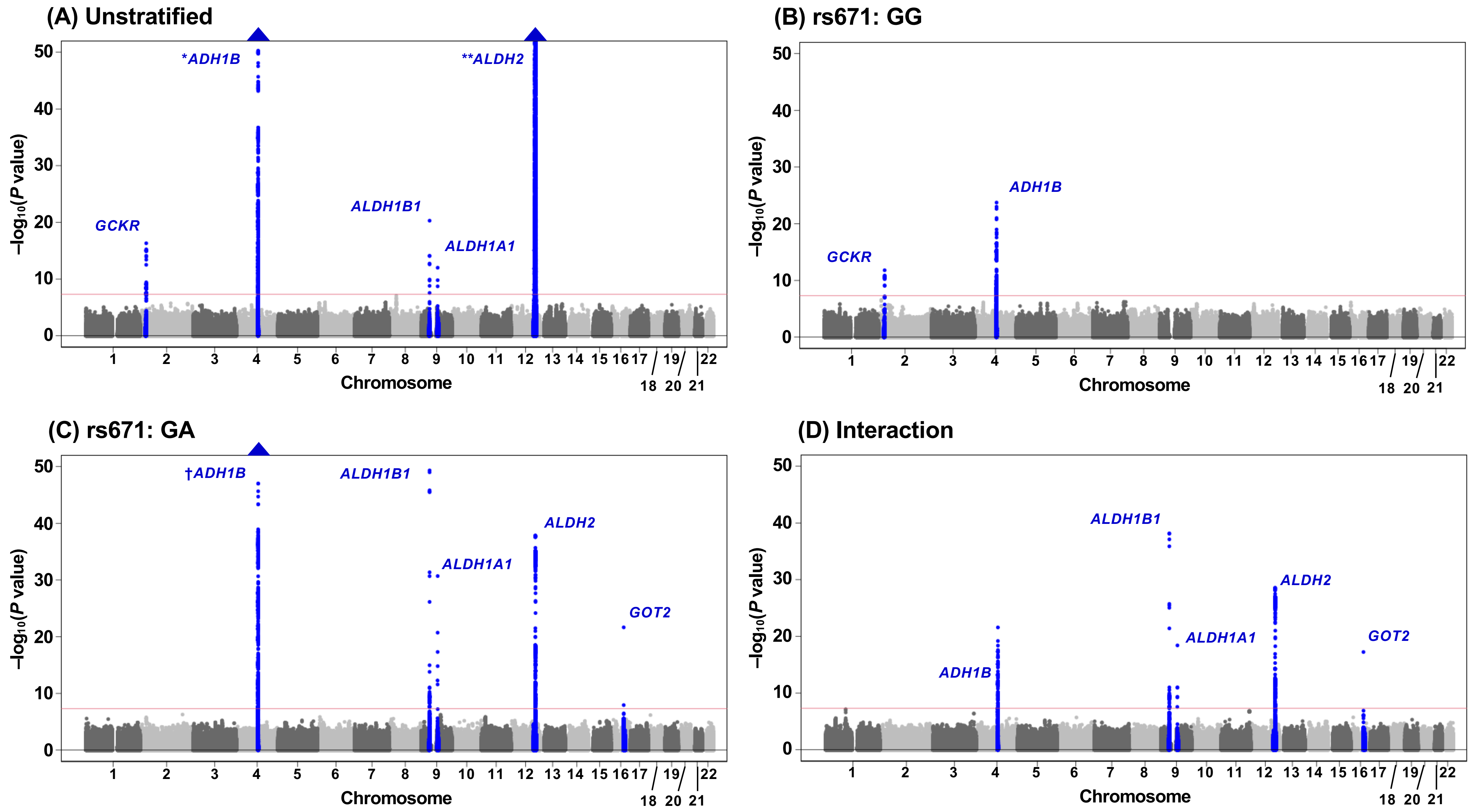

Fig. S4. Genomic loci reaching genome-wide significance in either analysis for association with drinking status

Direction of effects of identified variants other than rs671 is presented as a heatmap (with colors indicating associated normalized ORs). Estimates with a single asterisk show genome-wide significance ($P < 5.0 \times 10^{-8}$). Lead SNP in each locus is highlighted with its estimates in bold. SNP, single nucleotide polymorphism; Ref, reference allele; Alt, alternative allele; freq., frequency; OR, odds ratio; 95% CI, 95% confidence interval; Het*P*, *P* value from test of heterogeneity.

SNP<br>*Gene*	Locus	Position	Function	Ref/Alt	Alt freq.<br>Unstratified<br>GG<br>GA	OR (95% CI)<br>*P*<br>I^2, Het*P*			
						Unstratified	GG	GA	Interaction
**rs1260326**<br>*GCKR*	2p23.3	27730940	nonsynonymous	T/C	0.440<br>0.444<br>0.437	**1.07 (1.05, 1.08)***<br>4.90×10^{-17}<br>47.0, 0.04	**1.08 (1.06, 1.11)***<br>1.60×10^{-12}<br>54.6, 0.02	1.07 (1.04, 1.10)<br>1.68×10^{-6}<br>0, 0.58	1.00 (0.98, 1.02)<br>0.776<br>0, 0.45
**rs1229984**<br>*ADH1B*	4q23	100239319	nonsynonymous	T/C	0.243<br>0.248<br>0.236	**1.18 (1.16, 1.20)***<br>1.59×10^{-73}<br>0, 0.88	**1.14 (1.11, 1.17)***<br>1.86×10^{-24}<br>0, 0.66	**1.37 (1.33, 1.41)***<br>4.36×10^{-86}<br>0, 0.59	**1.12 (1.09, 1.14)***<br>2.67×10^{-22}<br>50.8, 0.03
**rs2228093**<br>*ALDH1B1*	9p13.2	38396002	nonsynonymous	C/T	0.342<br>0.339<br>0.346	0.94 (0.92, 0.95)*<br>7.76×10^{-15}<br>0, 1	1.03 (1.00, 1.05)<br>0.029<br>28.5, 0.17	**0.81 (0.78, 0.83)***<br>4.72×10^{-50}<br>53.3, 0.02	**0.87 (0.86, 0.89)***<br>6.78×10^{-39}<br>74.8, 1.91×10^{-5}
**rs3043**<br>*ALDH1B1*	9p13.2	38397355	UTR3	G/C	0.693<br>0.692<br>0.693	**0.92 (0.91, 0.94)***<br>5.01×10^{-21}<br>25.2, 0.20	1.00 (0.97, 1.02)<br>0.880<br>30.6, 0.16	0.81 (0.79, 0.84)*<br>3.24×10^{-46}<br>23.7, 0.22	0.89 (0.88, 0.91)*<br>1.88×10^{-26}<br>25.4, 0.20
**rs8187929**<br>*ALDH1A1*	9q21.13	75540504	nonsynonymous	T/A	0.034<br>0.034<br>0.034	**1.16 (1.12, 1.21)***<br>1.00×10^{-12}<br>0, 0.59	0.97 (0.92, 1.03)<br>0.392<br>15.0, 0.30	**1.53 (1.42, 1.64)***<br>2.02×10^{-31}<br>0, 0.51	**1.27 (1.20, 1.34)***<br>4.00×10^{-19}<br>47.4, 0.04
**rs440**<br>*ALDH2*	12q24.12	112228714	intronic	T/C	0.155<br>0.202<br>0.106	1.45 (1.42, 1.48)*<br>2.19×10^{-255}<br>2.7, 0.42	1.00 (0.98, 1.03)<br>0.797<br>9.4, 0.35	**0.73 (0.70, 0.77)***<br>1.46×10^{-38}<br>27.9, 0.18	0.84 (0.82, 0.87)*<br>3.12×10^{-29}<br>37.6, 0.10
**rs4646778**<br>*ALDH2*	12q24.12	112235783	intronic	C/A	0.155<br>0.202<br>0.106	1.45 (1.42, 1.48)*<br>9.12×10^{-256}<br>1.2, 0.43	1.00 (0.98, 1.03)<br>0.777<br>10.3, 0.35	0.73 (0.70, 0.77)*<br>1.52×10^{-38}<br>27.1, 0.19	**0.84 (0.82, 0.87)***<br>2.72×10^{-29}<br>38.0, 0.10
**rs671**<br>*ALDH2*	12q24.12	112241766	nonsynonymous	G/A	0.244<br>-<br>-	**0.16 (0.16, 0.16)***<br>3.39×10^{-4952}<br>47.0, 0.04			
**rs73550818**<br>*GOT2*	16q21	58764855	intronic	C/A	0.505<br>0.507<br>0.503	0.98 (0.96, 0.99)<br>0.002<br>0, 0.65	1.04 (1.02, 1.06)<br>0.001<br>0, 0.76	**0.87 (0.85, 0.90)***<br>2.26×10^{-22}<br>3.2, 0.41	**0.92 (0.90, 0.94)***<br>5.81×10^{-18}<br>48.1, 0.04

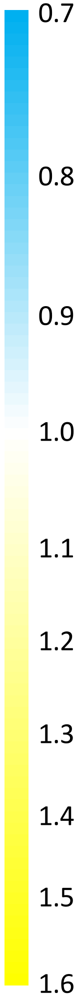

Fig. S5. Regional association plots of the identified regions for daily alcohol intake other than those shown in Figure 3

Regional association plots for (A) unstratified, (B) rs671 wild-type homozygotes (GG), (C) rs671 heterozygotes (GA), and (D) interaction with rs671 are shown. The vertical axis indicates the $-\log_{10}(P$ value) for the assessment of the association of each SNP with daily alcohol intake. Black line represents genome-wide significance threshold of 5.0×10^{-8}. The colors indicate the LD (r^2) between each lead SNP and neighboring SNPs based on the JPT population in the 1000 Genomes Project Phase 3.

(A) Unstratified

2p23.3 (rs1260326)

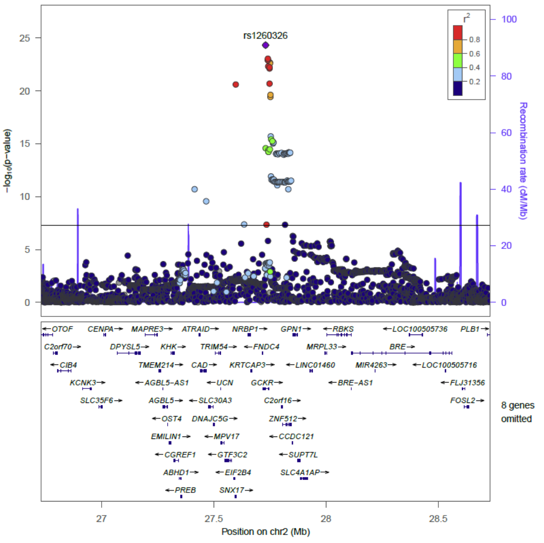

4p14 (rs28712821)

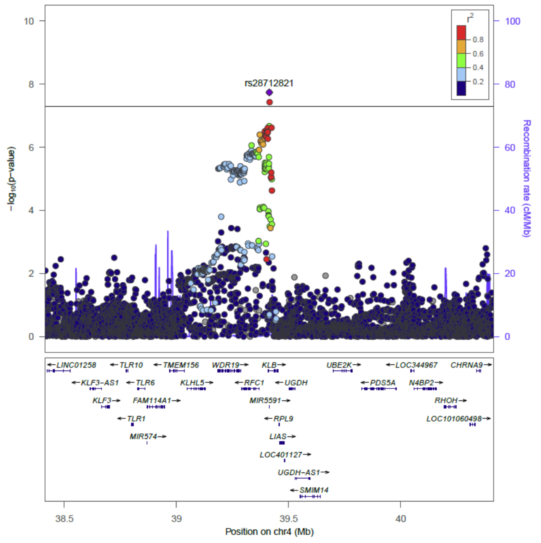

4q23 (rs1229984)

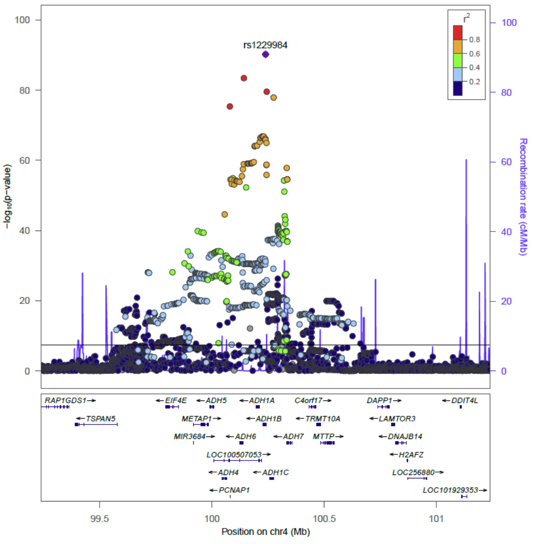

9p13.2(rs3043)

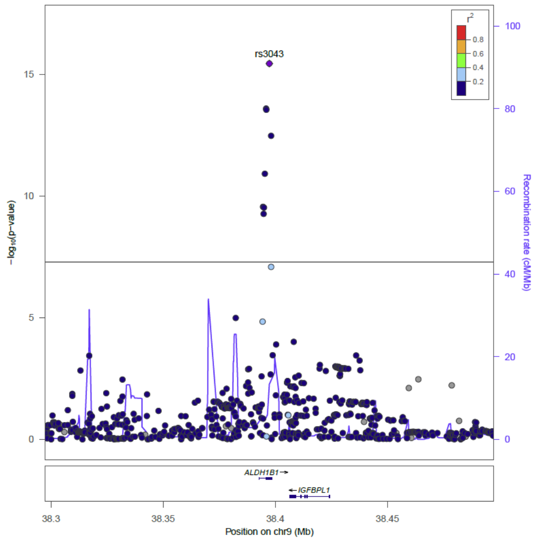

9q21.13 (rs8187929)

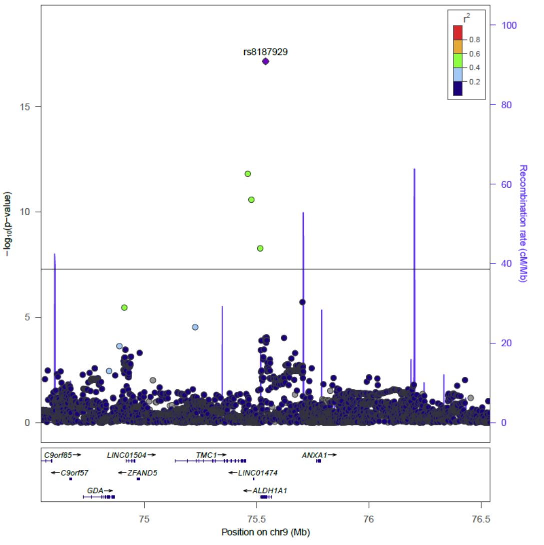

12q24.12 (rs671)

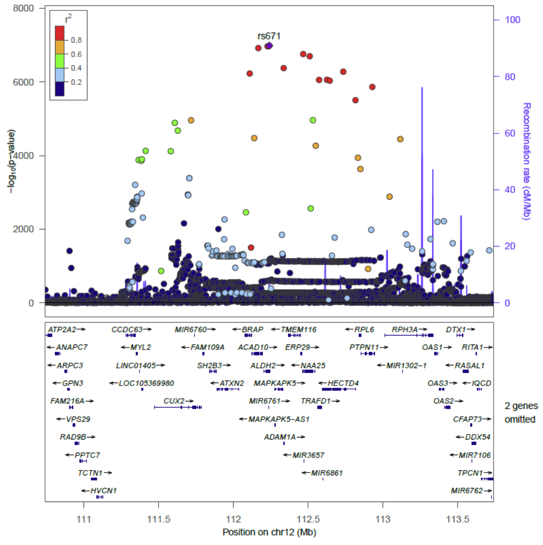

Fig. S5. Regional association plots of the identified regions for daily alcohol intake other than those shown in Figure 3

Regional association plots for (A) unstratified, (B) rs671 wild-type homozygotes (GG), (C) rs671 heterozygotes (GA), and (D) interaction with rs671 are shown. The vertical axis indicates the –log$_{10}$(P value) for the assessment of the association of each SNP with daily alcohol intake. Black line represents genome-wide significance threshold of 5.0×10^{-8}. The colors indicate the LD (r^2) between each lead SNP and neighboring SNPs based on the JPT population in the 1000 Genomes Project Phase 3.

(B) rs671:GG

2p23.3 (rs1260326)

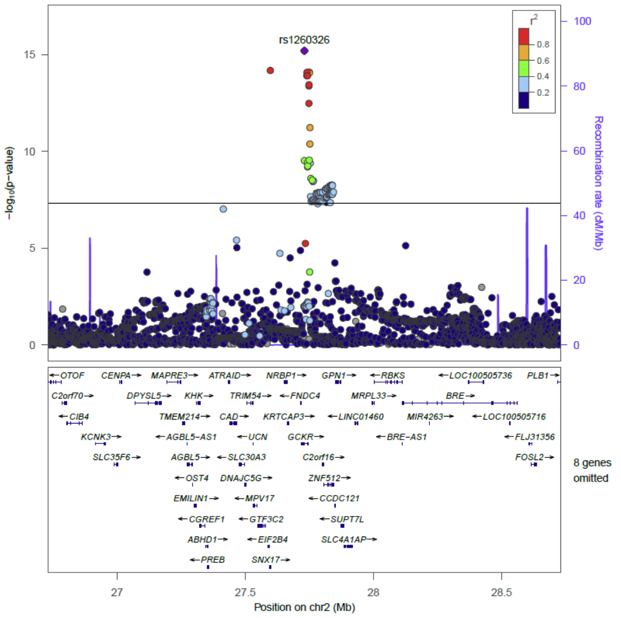

4p14 (rs28712821)

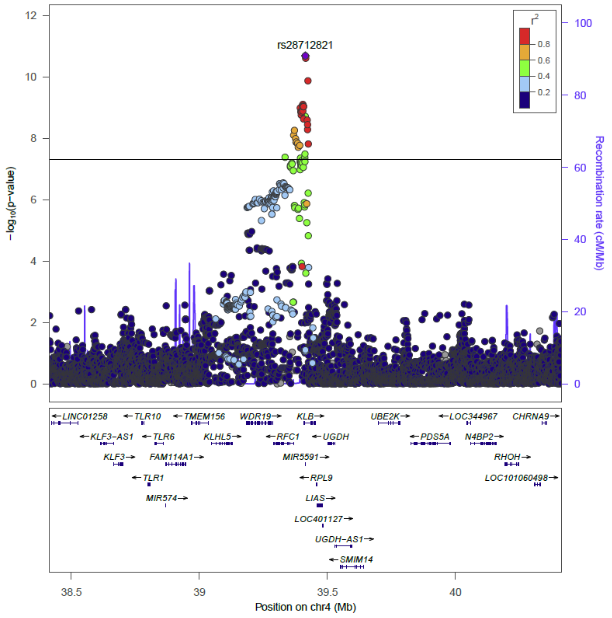

4q23 (rs1229984)

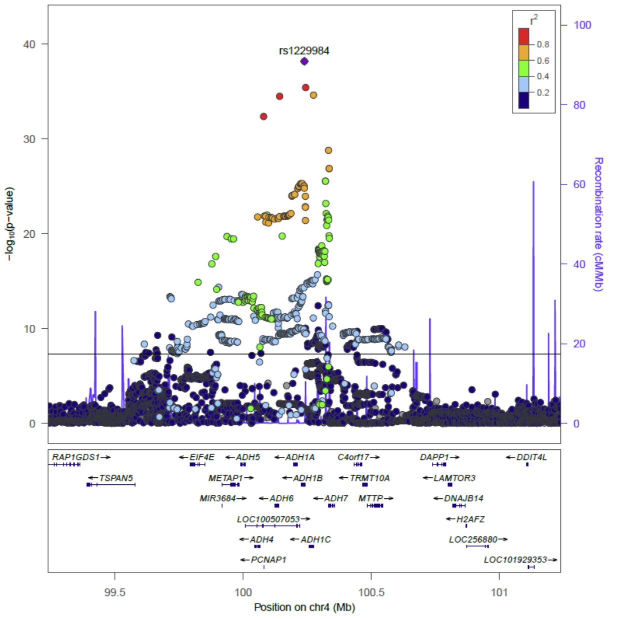

Fig. S5. Regional association plots of the identified regions for daily alcohol intake other than those shown in Figure 3

Regional association plots for (A) unstratified, (B) rs671 wild-type homozygotes (GG), (C) rs671 heterozygotes (GA), and (D) interaction with rs671 are shown. The vertical axis indicates the $-\log_{10}(P \text{ value})$ for the assessment of the association of each SNP with daily alcohol intake. Black line represents genome-wide significance threshold of 5.0×10^{-8}. The colors indicate the LD (r^2) between each lead SNP and neighboring SNPs based on the JPT population in the 1000 Genomes Project Phase 3.

(C) rs671:GA

2p23.3 (rs1260326)

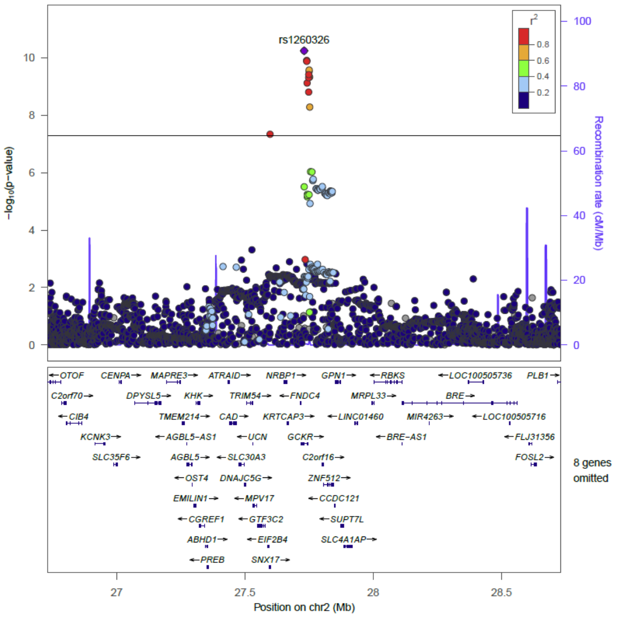

4q23 (rs1229984)

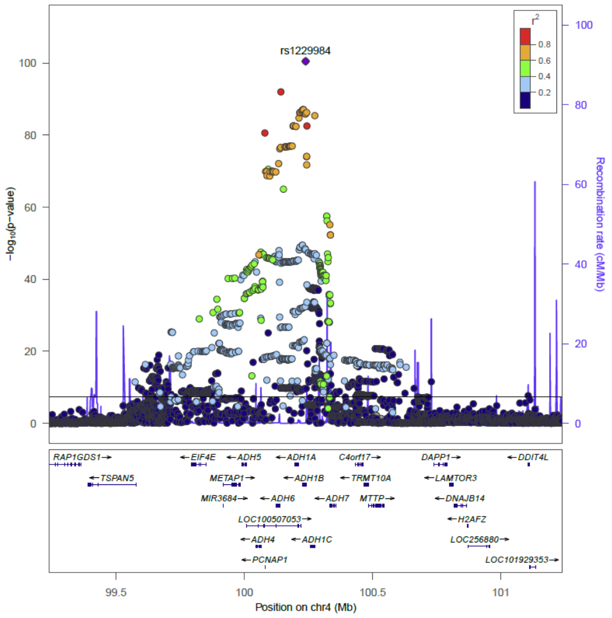

9p13.2 (rs2228093)

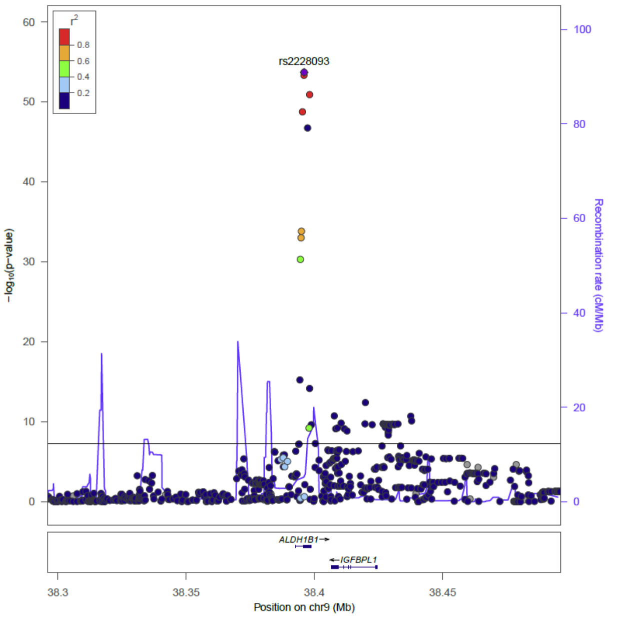

9q21.13 (rs8187929)

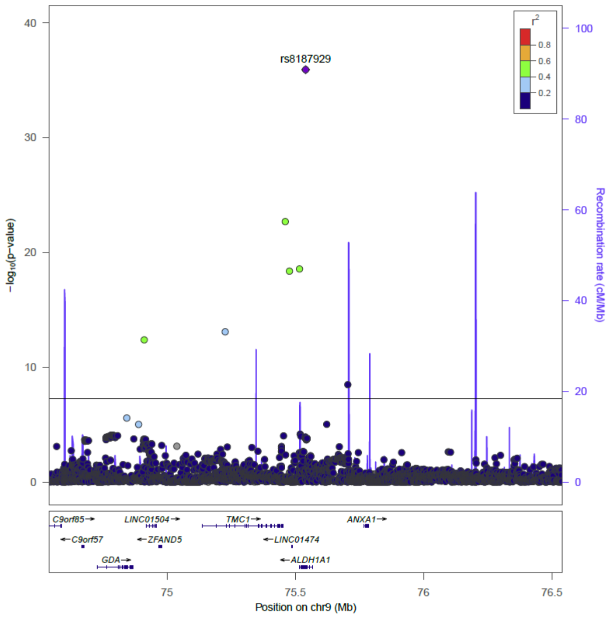

Fig. S5. Regional association plots of the identified regions for daily alcohol intake other than those shown in Figure 3

Regional association plots for (A) unstratified, (B) rs671 wild-type homozygotes (GG), (C) rs671 heterozygotes (GA), and (D) interaction with rs671 are shown. The vertical axis indicates the $-\log_{10}(P \text{ value})$ for the assessment of the association of each SNP with daily alcohol intake. Black line represents genome-wide significance threshold of 5.0×10^{-8}. The colors indicate the LD (r^2) between each lead SNP and neighboring SNPs based on the JPT population in the 1000 Genomes Project Phase 3.

(D) Interaction

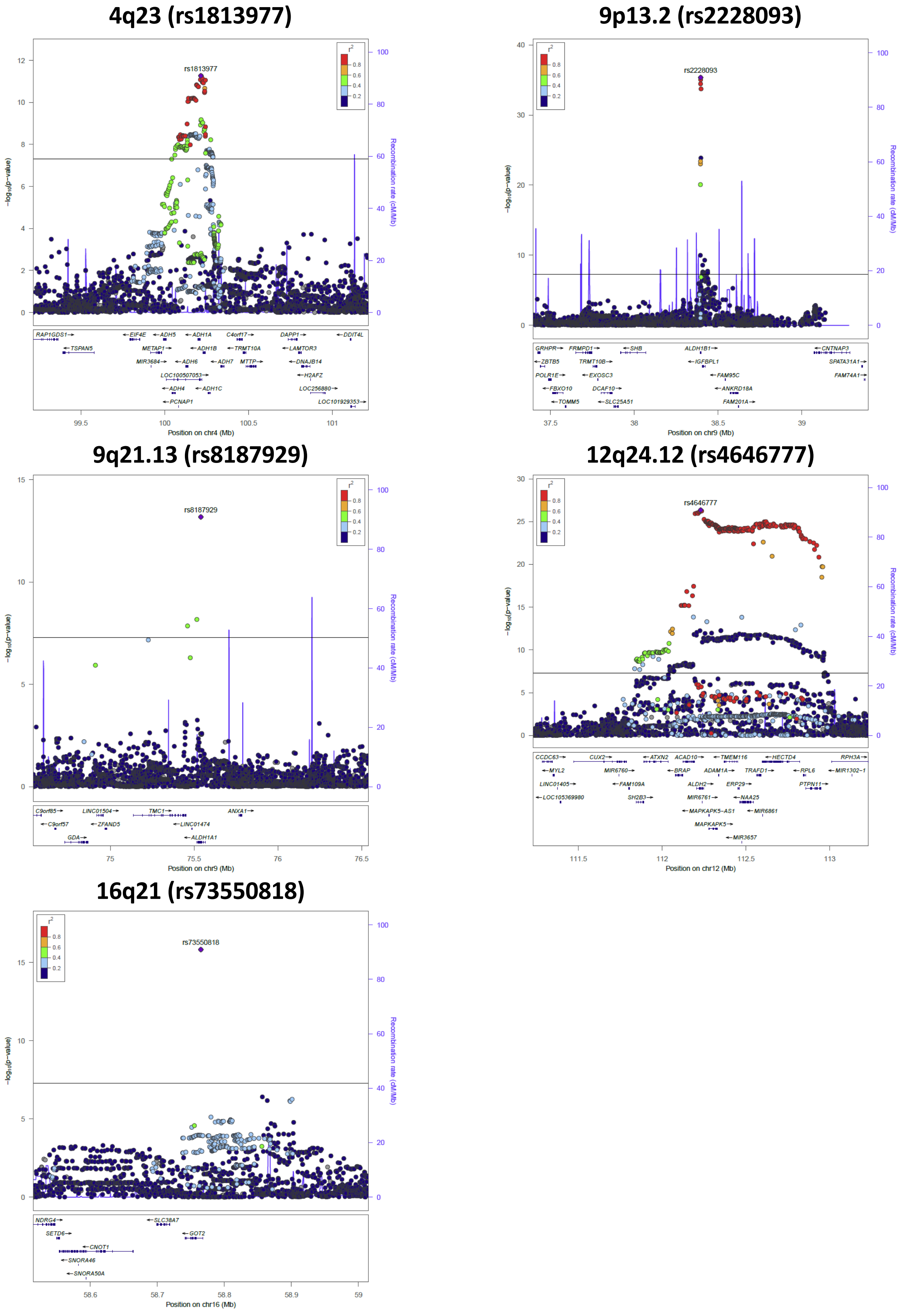

Fig. S6. Regional association plots of the identified regions for drinking status

Regional association plots for (A) unstratified, (B) rs671 wild-type homozygotes (GG), (C) rs671 heterozygotes (GA), and (D) interaction with rs671 are shown. The vertical axis indicates the $-\log_{10}$(P value) for the assessment of the association of each SNP with drinking status. Black line represents genome-wide significance threshold of 5.0×10^{-8}. The colors indicate the LD (r^2) between each lead SNP and neighboring SNPs based on the JPT population in the 1000 Genomes Project Phase 3.

(A) Unstratified

2p23.3 (rs1260326)

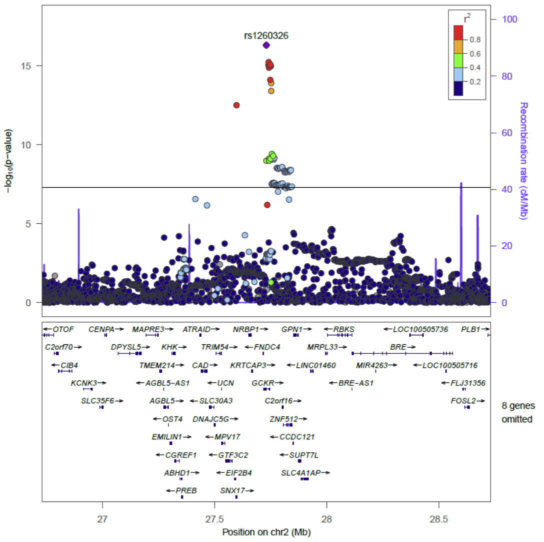

4q23 (rs1229984)

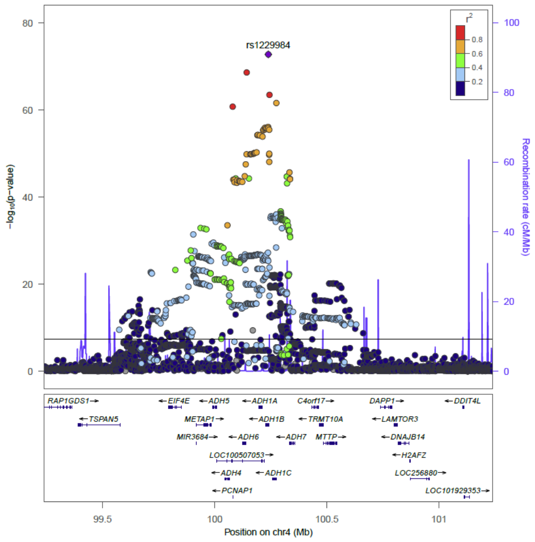

9p13.2 (rs3043)

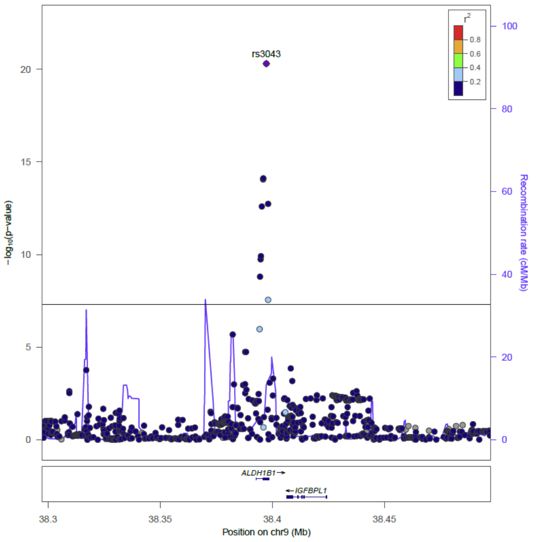

9q21.13 (rs8187929)

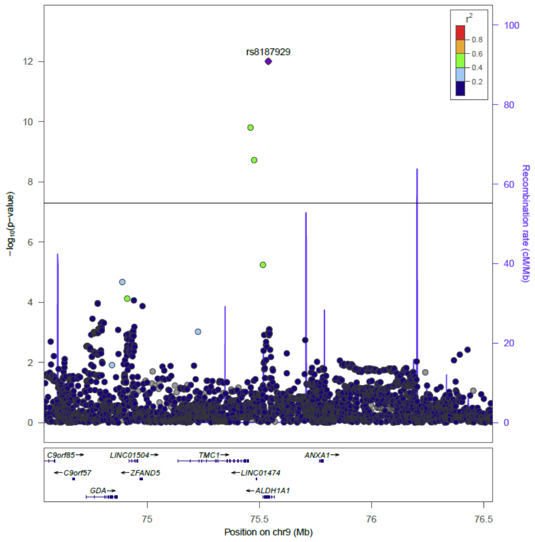

12q24.12 (rs671)

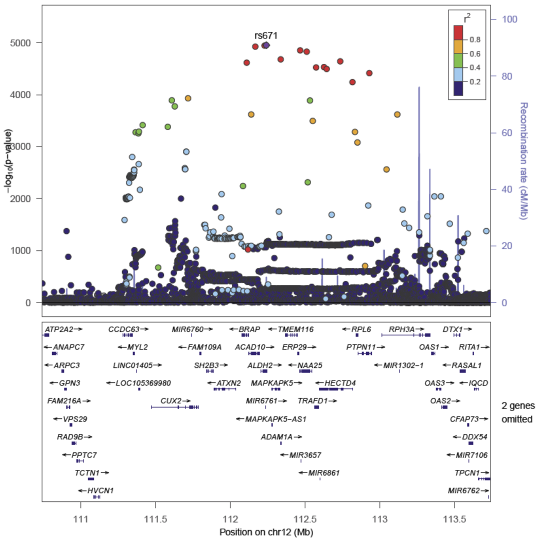

Fig. S6. Regional association plots of the identified regions for drinking status

Regional association plots for (A) unstratified, (B) rs671 wild-type homozygotes (GG), (C) rs671 heterozygotes (GA), and (D) interaction with rs671 are shown. The vertical axis indicates the $-\log_{10}(P\text{ value})$ for the assessment of the association of each SNP with drinking status. Black line represents genome-wide significance threshold of 5.0×10^{-8}. The colors indicate the LD (r^2) between each lead SNP and neighboring SNPs based on the JPT population in the 1000 Genomes Project Phase 3.

(B) rs671:GG

2p23.3 (rs1260326)

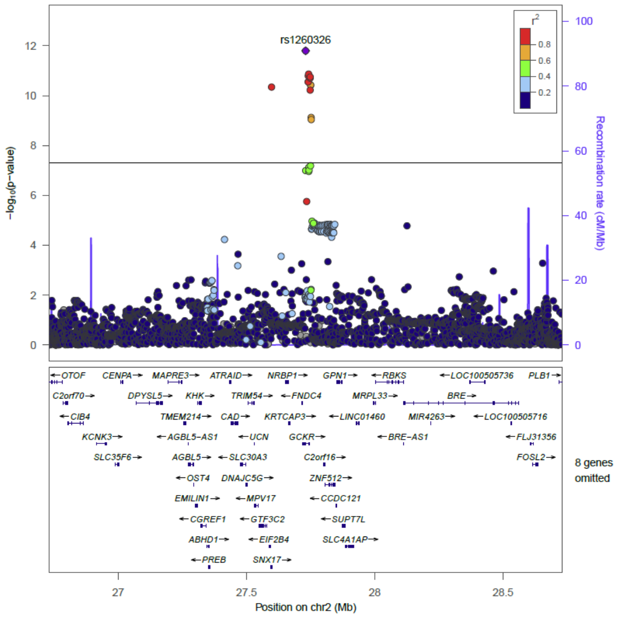

4q23 (rs1229984)

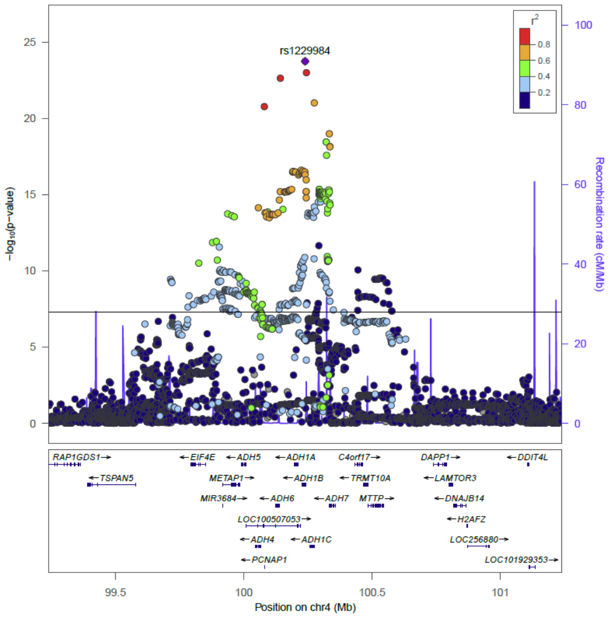

Fig. S6. Regional association plots of the identified regions for drinking status
Regional association plots for (A) unstratified, (B) rs671 wild-type homozygotes (GG), (C) rs671 heterozygotes (GA), and (D) interaction with rs671 are shown. The vertical axis indicates the $-\log_{10}(P \text{ value})$ for the assessment of the association of each SNP with drinking status. Black line represents genome-wide significance threshold of 5.0×10^{-8}. The colors indicate the LD (r^2) between each lead SNP and neighboring SNPs based on the JPT population in the 1000 Genomes Project Phase 3.

(C) rs671:GA

4q23 (rs1229984)

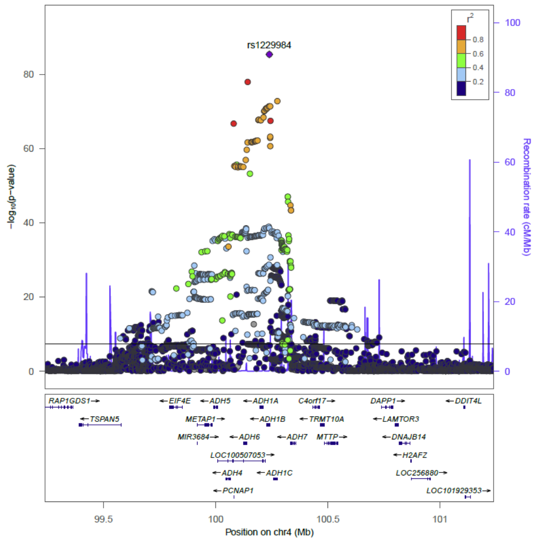

9p13.2 (rs2228093)

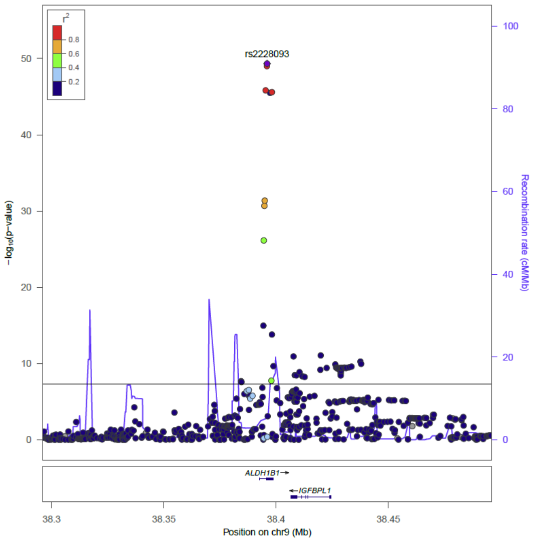

9q21.13 (rs8187929)

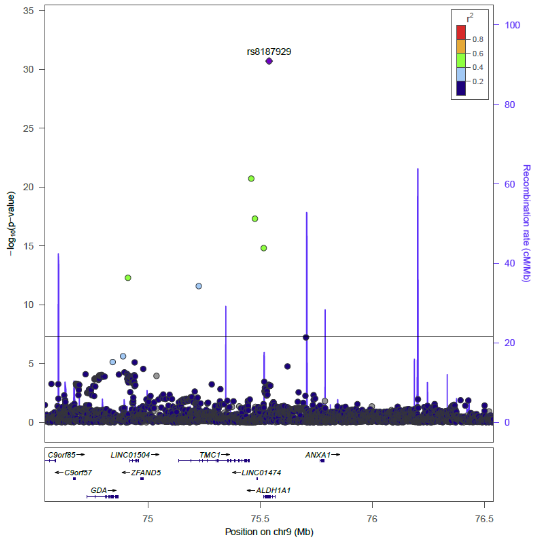

12q24.12 (rs440)

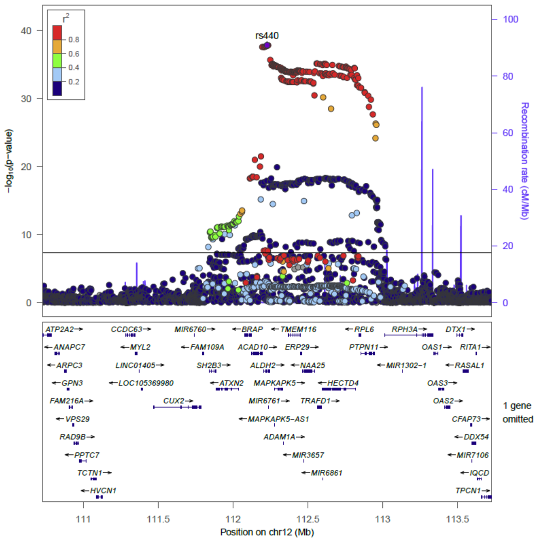

16q21 (rs73550818)

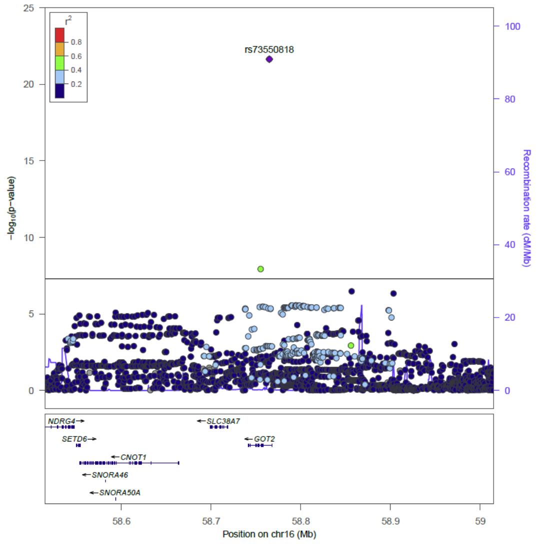

Fig. S6. Regional association plots of the identified regions for drinking status

Regional association plots for (A) unstratified, (B) rs671 wild-type homozygotes (GG), (C) rs671 heterozygotes (GA), and (D) interaction with rs671 are shown. The vertical axis indicates the $-\log_{10}(P \text{ value})$ for the assessment of the association of each SNP with drinking status. Black line represents genome-wide significance threshold of 5.0×10^{-8}. The colors indicate the LD (r^2) between each lead SNP and neighboring SNPs based on the JPT population in the 1000 Genomes Project Phase 3.

(D) Interaction

4q23 (rs1229984)

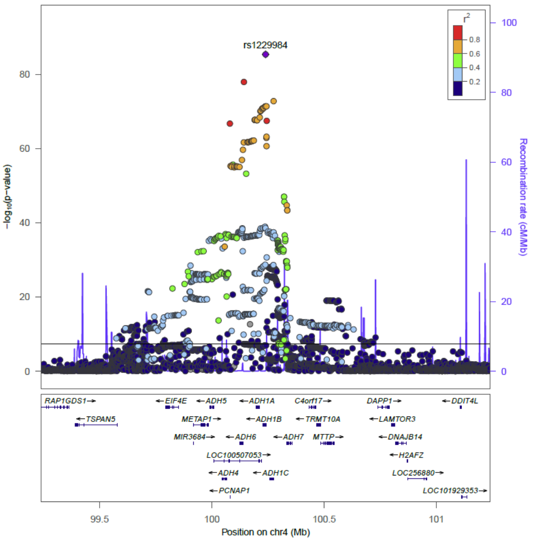

9p13.2 (rs2228093)

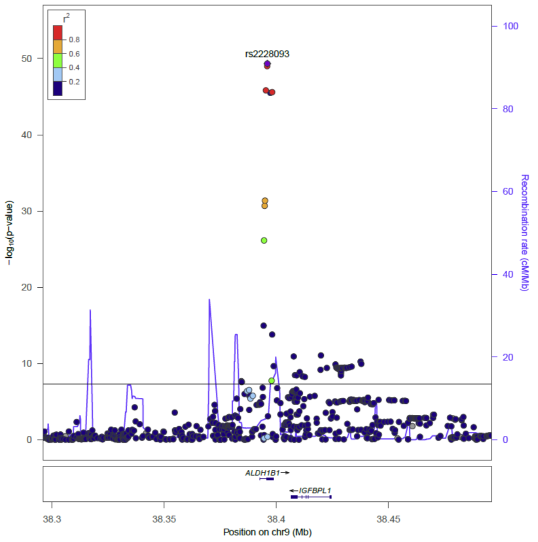

9q21.13 (rs8187929)

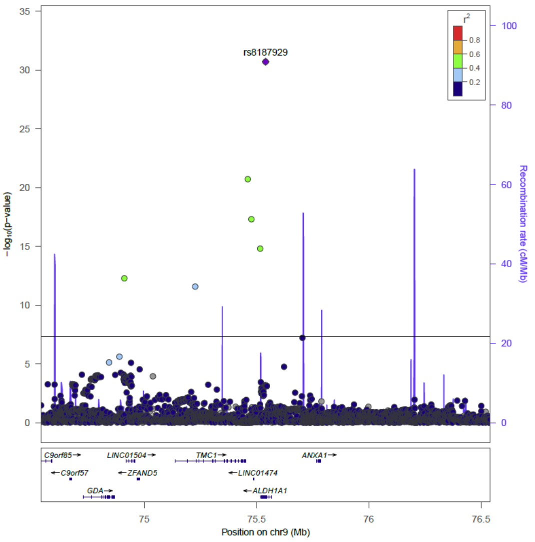

12q24.12 (rs4646778)

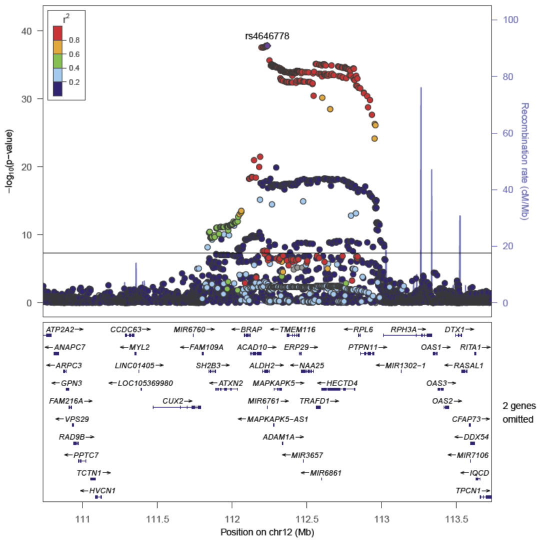

16q21 (rs73550818)

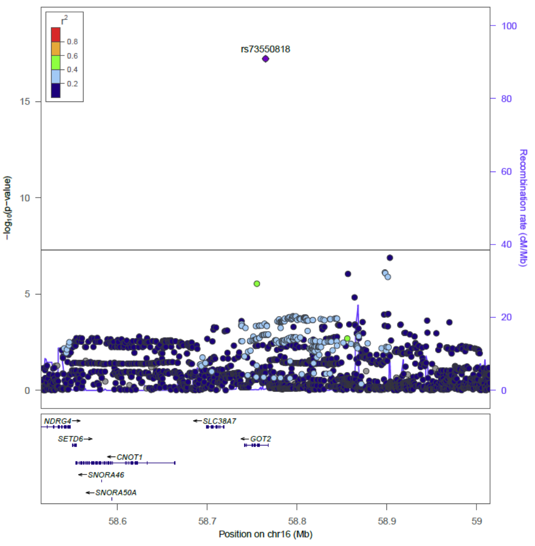

Fig. S7. Flowchart of the filtering process of JMA to control false positives
Manhattan plots of the JMA for daily alcohol intake according to different QC filtering are presented. The position on each chromosome (x-axis) and the observed $-\log_{10}(P\ \text{value})$ (y-axis) of all tested genetic variants are shown. The solid red line indicates genome-wide significance level. The genome-wide significant SNPs are highlighted in red. *P* values were corrected for genomic control.

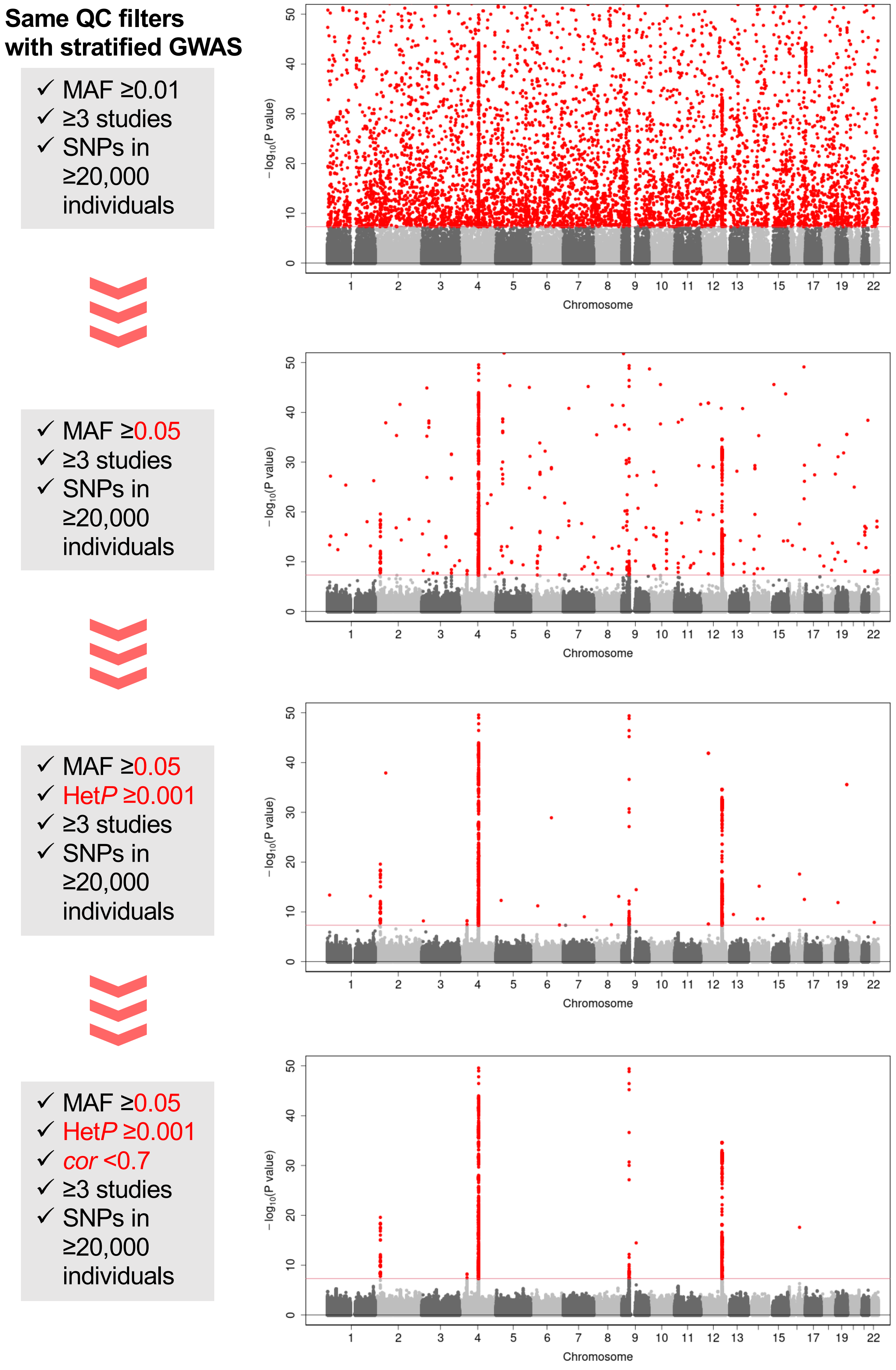

Fig. S8. Manhattan plots and Q-Q plots of JMA for daily alcohol intake

In the Manhattan plots, the position on each chromosome (x-axis) and the observed $-\log_{10}$(P value) (y-axis) of all tested genetic variants are shown. The solid red line indicates genome-wide significance level. Blue triangles represent loci containing SNPs with P values of $<1 \times 10^{-50}$. **ADH1B* JMA P value: 3.27×10^{-95}. In the QQ plots, the vertical and horizontal axes indicate the observed and expected $-\log_{10}$(P value) for tests of association between SNPs and daily alcohol intake, respectively. P values were adjusted after genomic control. rs8187929 at 9q21.13 was not included in the analysis due to MAF<5%.

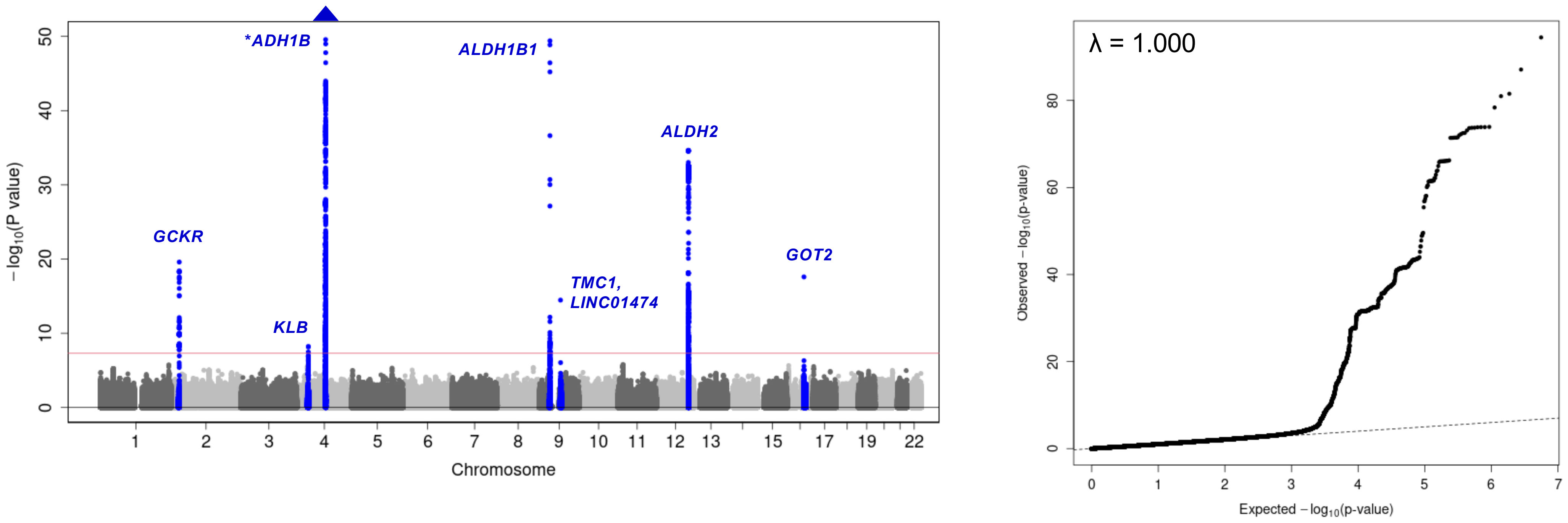

Fig. S9. Regional association plots of the identified regions in JMA for daily alcohol intake

The vertical axis indicates the –$\log_{10}$(P value) for the assessment of the association of each SNP with daily alcohol intake. Black line represents genome-wide significance threshold of 5.0×10^{-8}. The colors indicate the LD (r^2) between each lead SNP and neighboring SNPs based on the JPT population in the 1000 Genomes Project Phase 3. P values were adjusted after genomic control. rs8187929 at 9q21.13 was not included in the analysis due to MAF<5%.

2p23.3 (rs1260326)

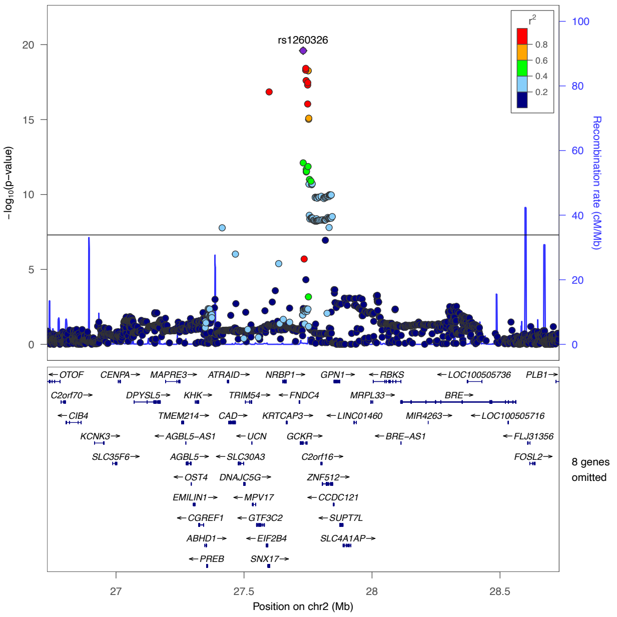

4p14 (rs28712821)

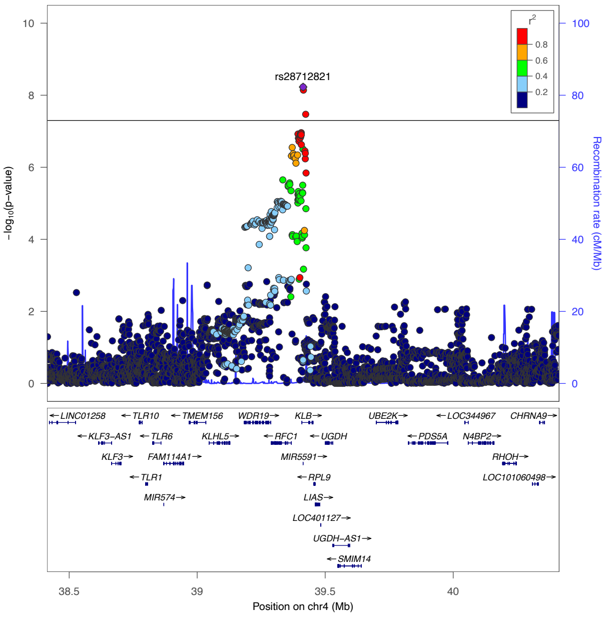

4q23 (rs1229984)

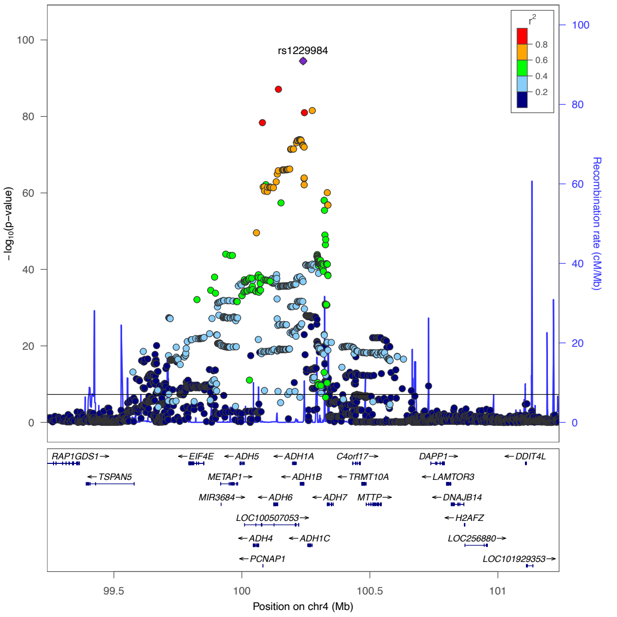

9p13.2 (rs2228093)

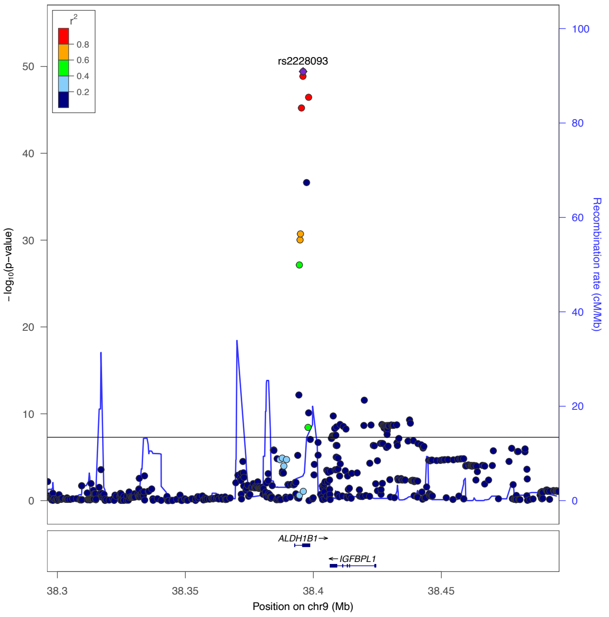

Fig. S9. Regional association plots of the identified regions in JMA for daily alcohol intake

The vertical axis indicates the $-\log_{10}$(*P* value) for the assessment of the association of each SNP with daily alcohol intake. Black line represents genome-wide significance threshold of 5.0×10^{-8}. The colors indicate the LD (r^2) between each lead SNP and neighboring SNPs based on the JPT population in the 1000 Genomes Project Phase 3. *P* values were adjusted after genomic control. rs8187929 at 9q21.13 was not included in the analysis due to MAF<5%.

9q21.13 (rs117986192)

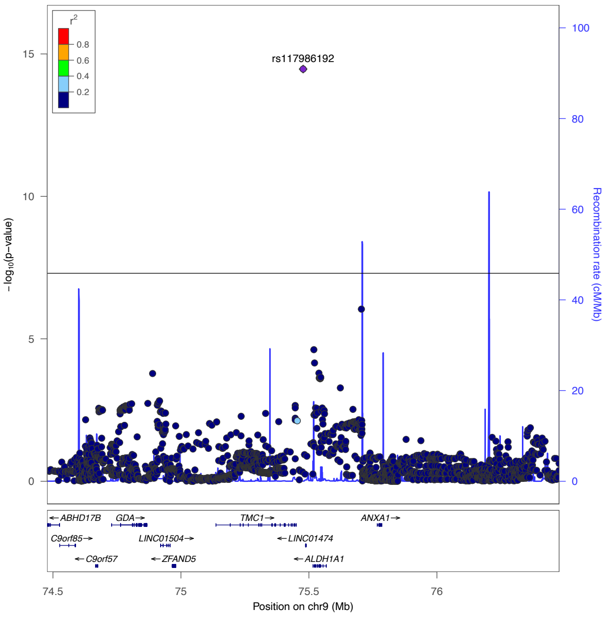

12q24.12 (rs4648328)

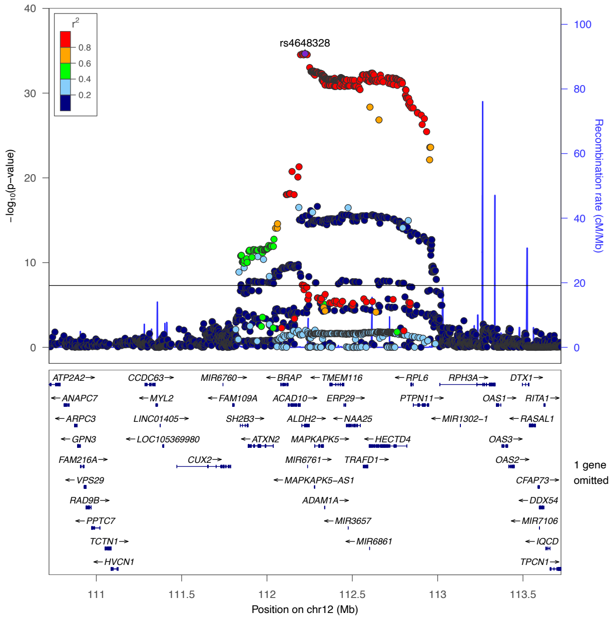

16q21 (rs73550818)

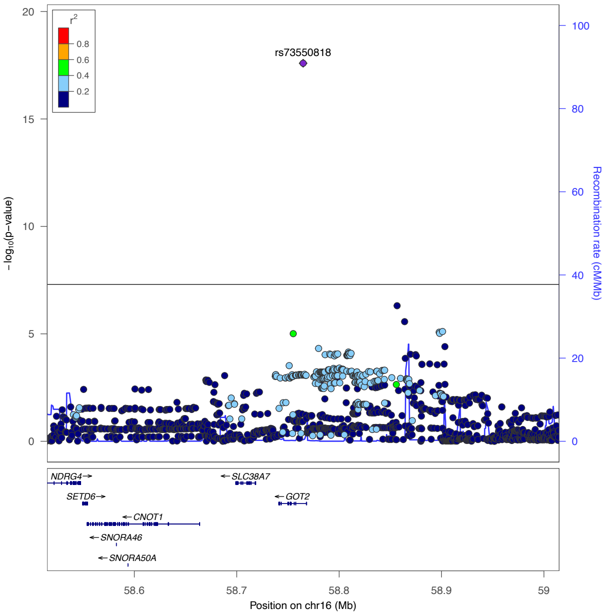

Fig. S10. Manhattan plots and Q-Q plots of JMA for drinking status

In the Manhattan plots, the position on each chromosome (x-axis) and the observed $-\log_{10}$(P value) (y-axis) of all tested genetic variants are shown. The solid red line indicates genome-wide significance level. Blue triangles represent loci containing SNPs with P values of $<1 \times 10^{-50}$. *$ADH1B$ JMA P value: 6.65×10^{-100}. In the QQ plots, the vertical and horizontal axes indicate the observed and expected $-\log_{10}$(P value) for tests of association between SNPs and drinking status, respectively. P values were adjusted after genomic control. rs8187929 at 9q21.13 was not included in the analysis due to MAF<5%.

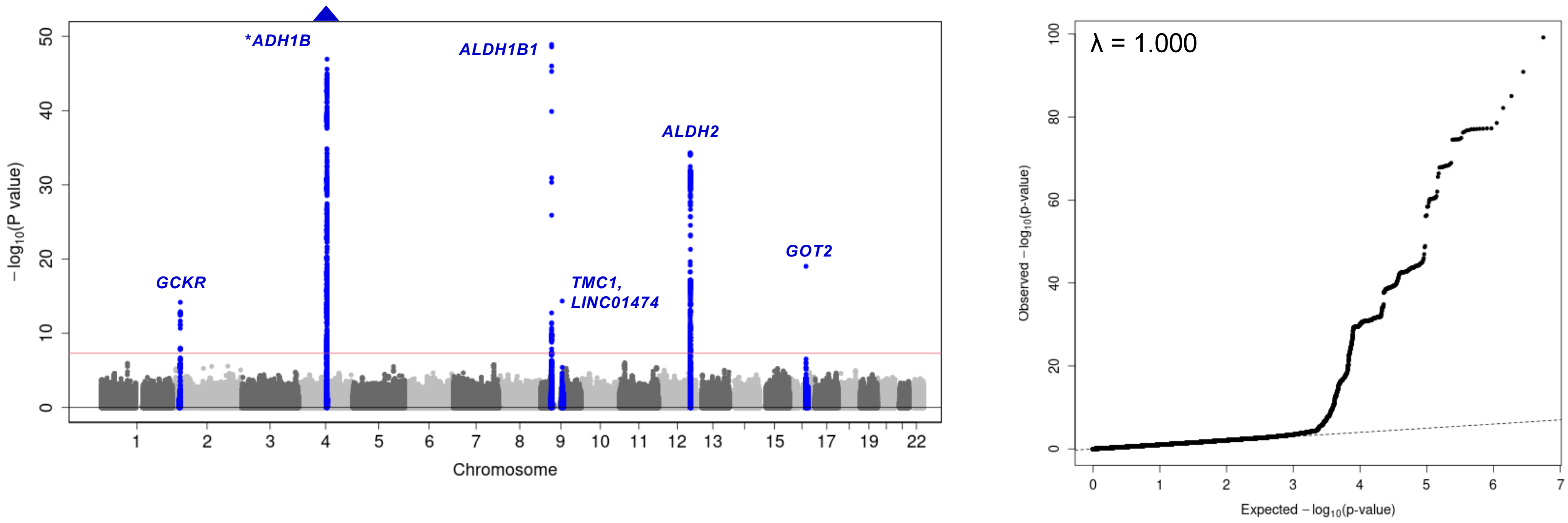

Fig. S11. Regional association plots of the identified regions in JMA for drinking status

The vertical axis indicates the $-\log_{10}$(P value) for the assessment of the association of each SNP with drinking status. Black line represents genome-wide significance threshold of 5.0×10^{-8}. The colors indicate the LD (r^2) between each lead SNP and neighboring SNPs based on the JPT population in the 1000 Genomes Project Phase 3. P values were adjusted after genomic control. rs8187929 at 9q21.13 was not included in the analysis due to MAF<5%.

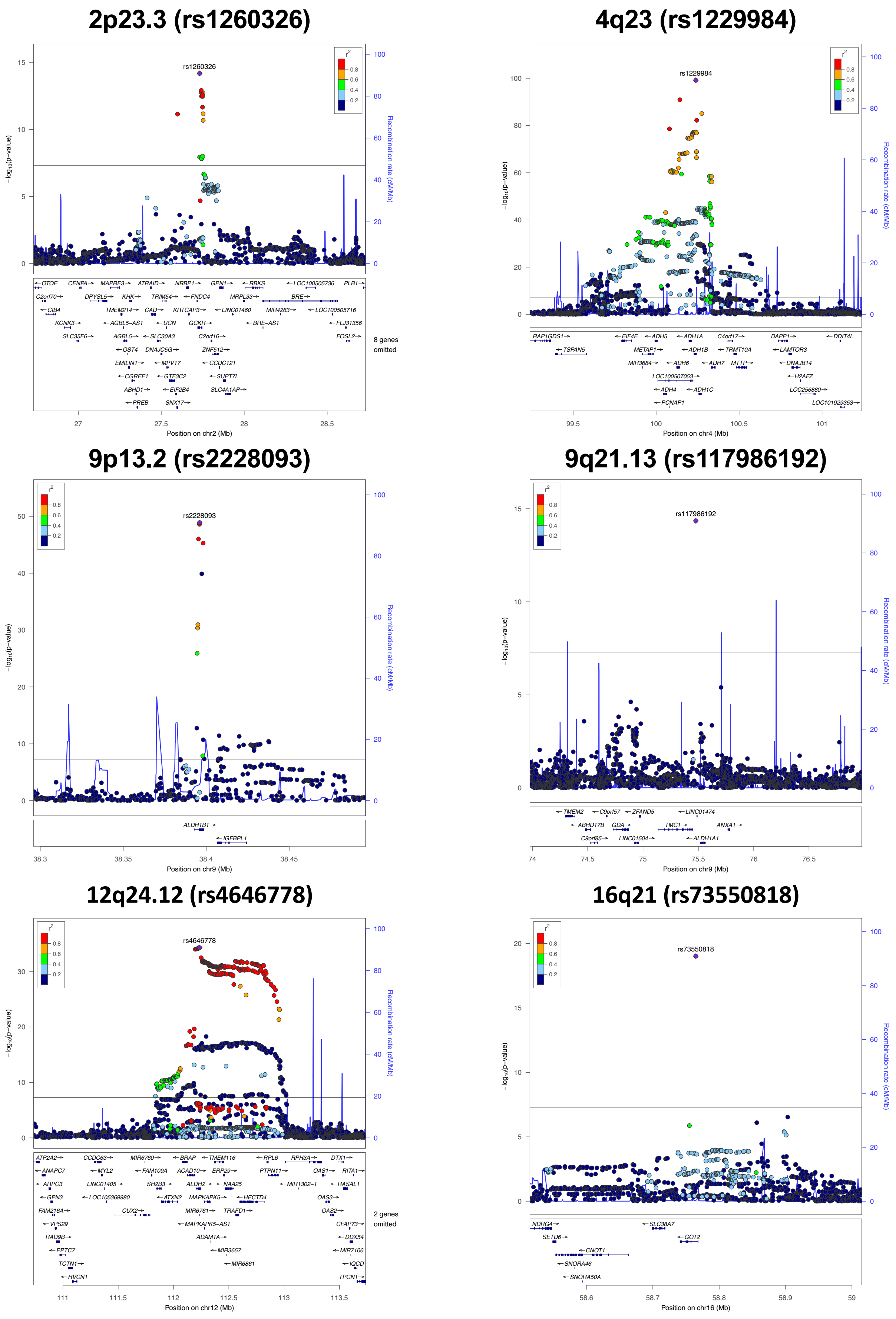

Fig. S12. LD maps of rs671 and 70 SNPs at 12q24.12 which showed *P*-value < 1×10^{-30} with daily alcohol intake for rs671 heterozygotes (GA) based on 1000 Genomes Phase 3 JPT

Pairwise linkage disequilibrium (A) r^2 values (white to black scales indicate low to high values) and (B) *D'* values (white to red scales indicate low to high values), as determined using Haploview software. The SNPs surrounded by red rectangles indicate rs79463616, rs4648328, rs4646777, rs440, rs4646778, and rs671.

(A) r^2

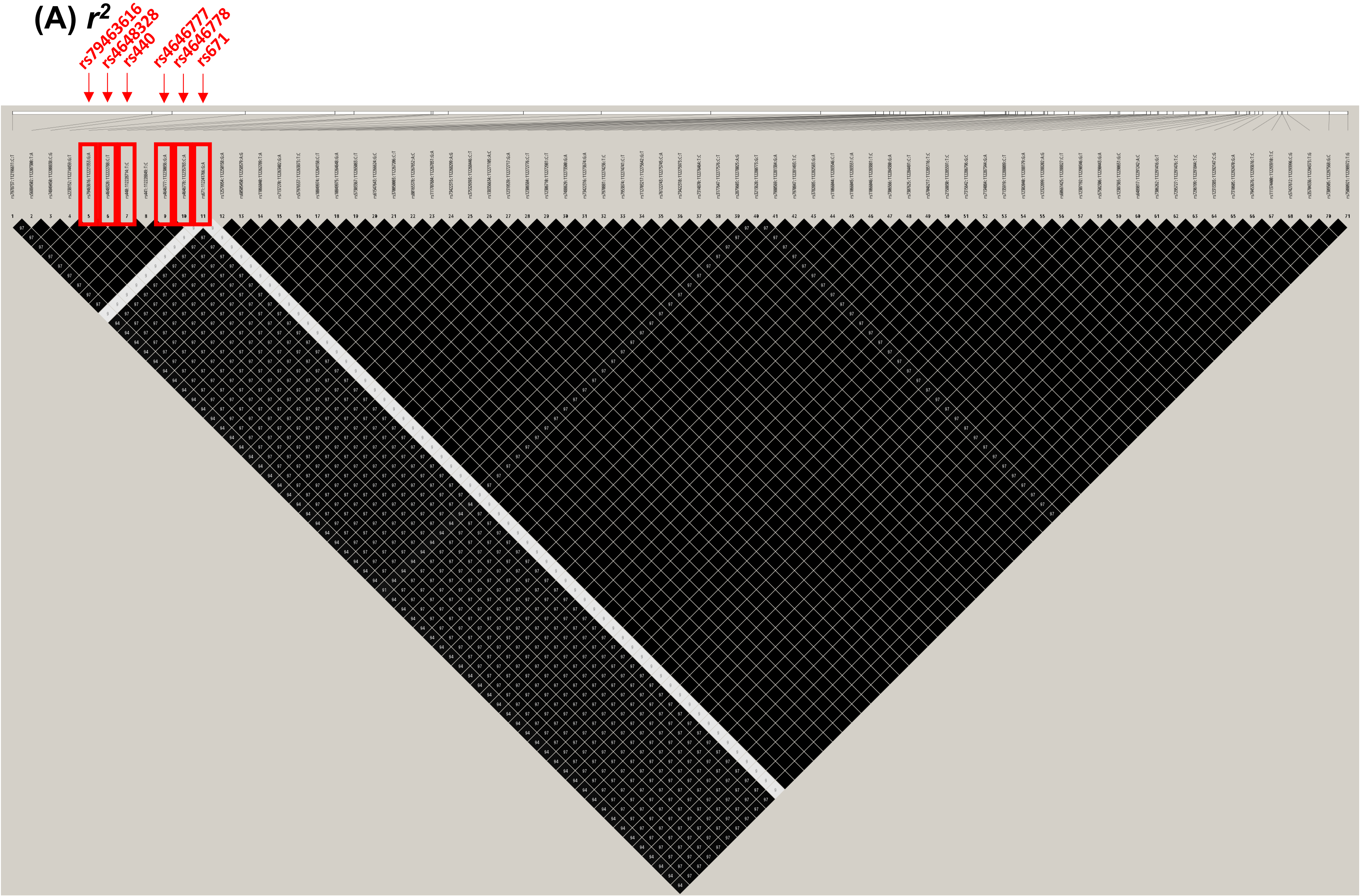

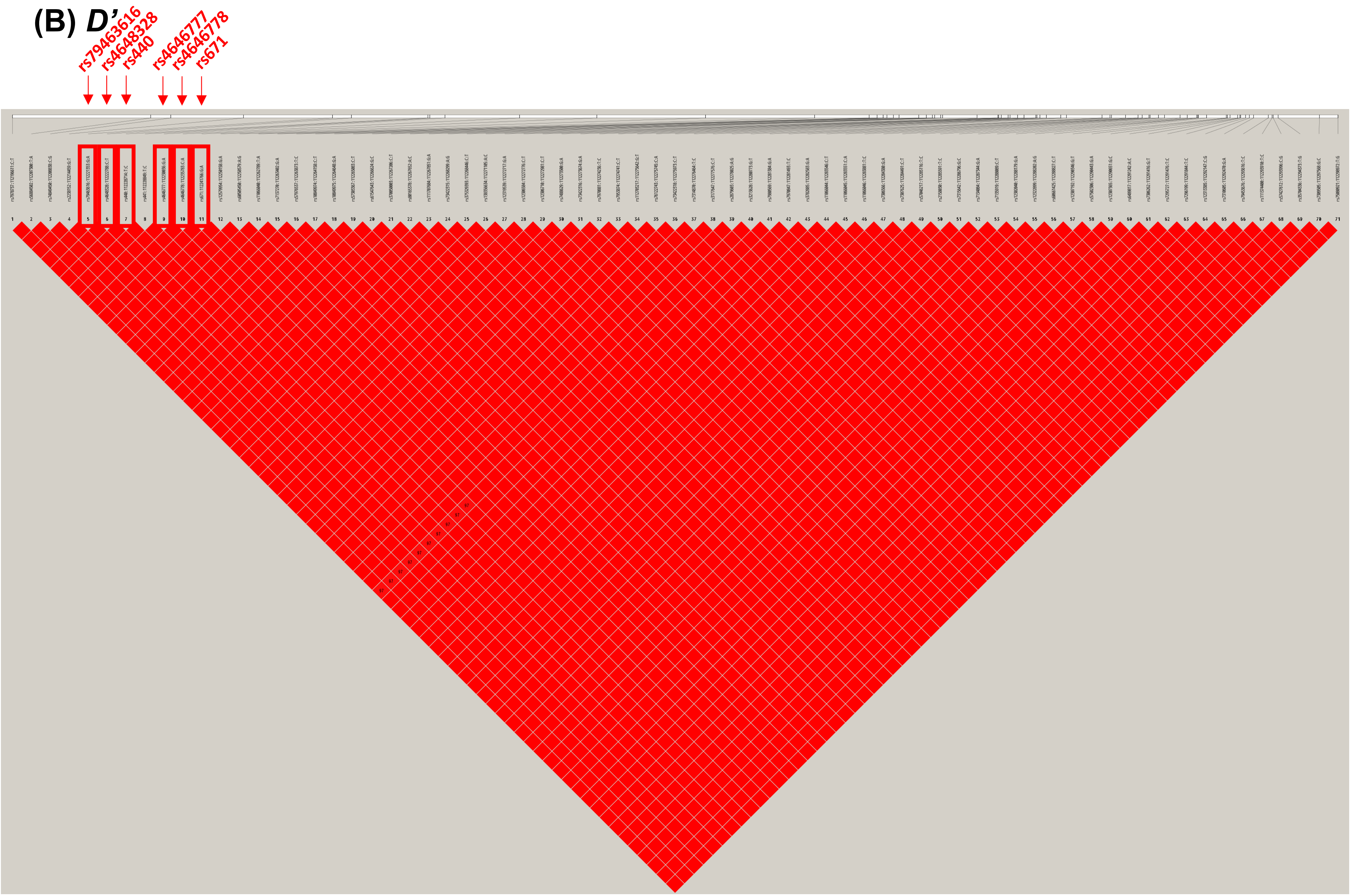
(B) D'
rs79463616
rs4648328
rs440
rs4646777
rs4646778
rs671

Fig. S13. Haplotypes of s671 and 70 SNPs at 12q24.12 which showed *P*-value < 1 × 10^{-30} with daily alcohol intake for rs671 heterozygotes (GA) based on 1000 Genomes Phase 3 JPT

The upper numbers correspond to the SNP numbers shown in Supplementary Figure 7. The haplotype surrounded by blue square haplotype with A allele of rs671. The haplotypes were estimated by using Haploview software. These three haplotypes explain >98%.

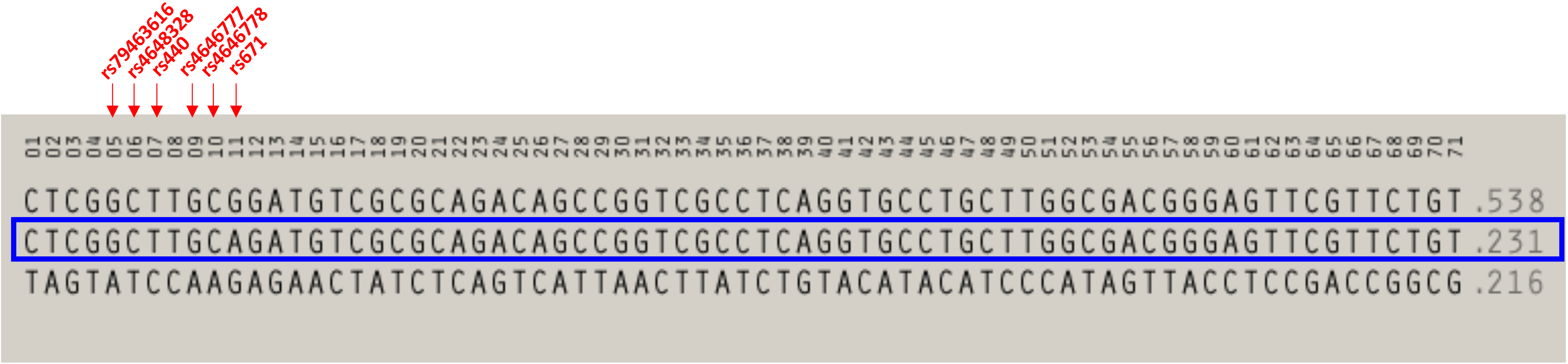

Fig. S14. LD maps and haplotypes of rs2228093 and seven SNPs at 9p13.2 which showed *P*-value < 1×10^{-30} with daily alcohol intake for rs671 heterozygotes (GA) based on 1000 Genomes Phase 3 JPT

Pairwise linkage disequilibrium (A) r^2 values (white to black scales indicate low to high values) and (B) *D'* values (white to red scales indicate low to high values), as determined using Haploview software. The SNPs surrounded by red rectangles indicate rs2228093 and rs3043. (C) The haplotypes were estimated by using Haploview software. The upper numbers correspond to the SNP numbers shown in (A) and (B).

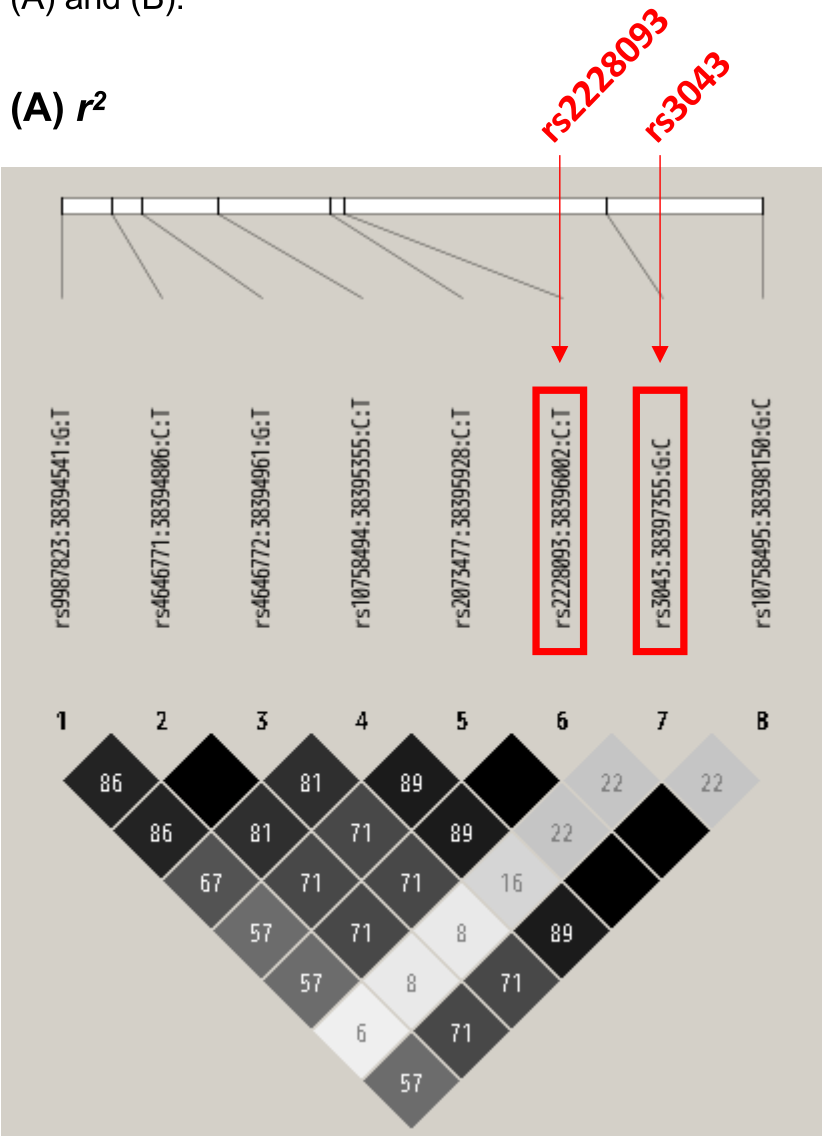

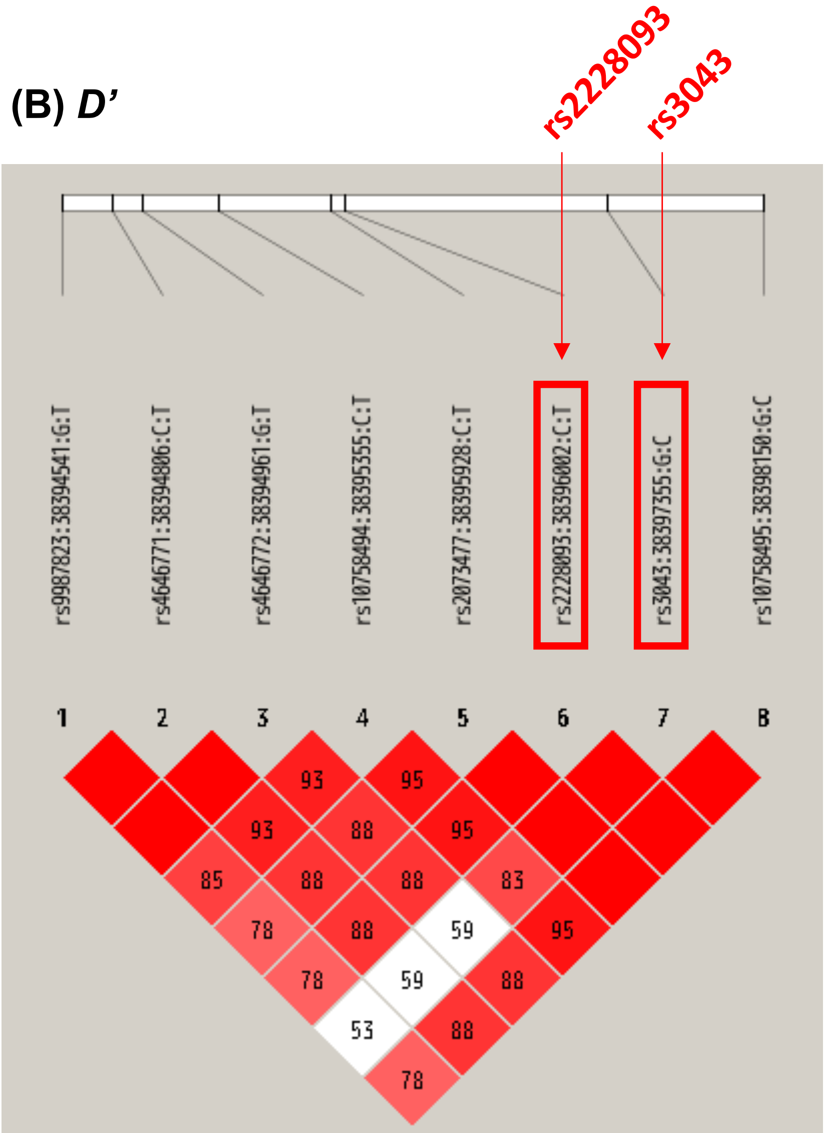

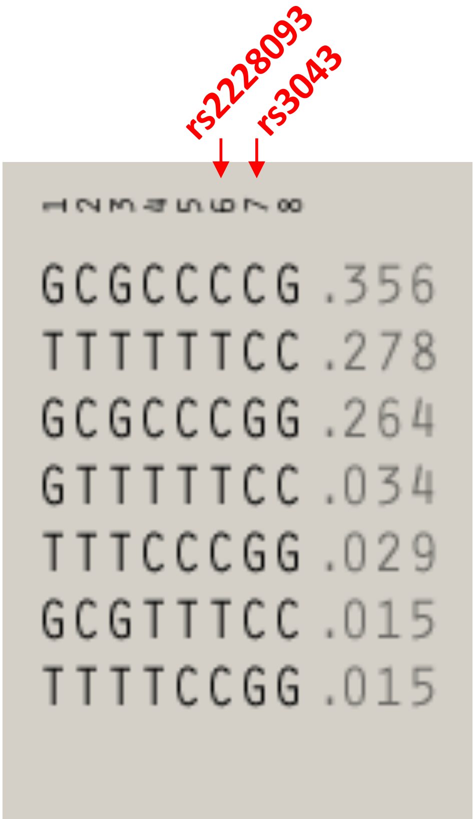

Fig. S15. Impact of identified variants on esophageal cancer risk and assessment of additive interaction of each SNP (1-allele change) with rs671 (GA vs. GG) : sensitivity analysis restricted to cases with squamous cell carcinoma

SNP *Gene* Ref/Alt	Analysis	OR (95% CI)	*P*	Het*P*	I^2	Interaction analysis	RERI (95% CI)	*P*
**rs671**	Unstratified	**1.92 (1.43, 2.58)**	**1.83×10^{-5}**	0.01	83.4			
ALDH2								
G/A								
**rs1260326**	Unstratified	**1.26 (1.13, 1.39)**	**2.06×10^{-5}**	0.43	0.0	rs1260326		
GCKR	GG	1.21 (0.99, 1.48)	0.0571	0.34	0.0	rs671		
T/C	GA	**1.31 (1.15, 1.50)**	**4.45×10^{-5}**	0.72	0.0	joint	**1.14 (0.45, 1.83)**	**0.0012**
**rs28712821**	Unstratified	**1.17 (1.05, 1.30)**	**0.0035**	0.93	0.0	rs28712821		
KLB	GG	1.29 (1.06, 1.58)	0.0113	0.74	0.0	rs671		
G/A	GA	1.14 (1.00, 1.30)	0.0542	0.99	0.0	joint	0.39 (-0.32, 1.09)	0.2804
**rs1229984**	Unstratified	**2.31 (2.07, 2.58)**	**7.73×10^{-45}**	0.46	0.0	rs1229984		
ADH1B	GG	**2.07 (1.64, 2.61)**	**1.48×10^{-9}**	0.28	14.1	rs671		
T/C	GA	**2.36 (2.05, 2.71)**	**1.29×10^{-30}**	0.66	0.0	joint	**4.37 (2.97, 5.78)**	**9.98×10^{-10}**
**rs2228093**	Unstratified	0.93 (0.81, 1.05)	0.2468	0.24	26.9	rs2228093		
ALDH1B1	GG	1.02 (0.74, 1.40)	0.9297	0.13	56.0	rs671		
C/T	GA	0.86 (0.75, 0.99)	0.0356	0.84	0.0	joint	-0.70 (-1.50, 0.11)	0.0894
**rs8187929**	Unstratified	**1.62 (1.25, 2.10)**	**0.0003**	0.32	0.0	rs8187929		
ALDH1A1	GG	1.11 (0.65, 1.91)	0.7131	0.41	0.0	rs671		
T/A	GA	**1.83 (1.24, 2.71)**	**0.0024**	0.24	27.0	joint	3.71 (-0.10, 7.53)	0.0564
**rs4648328**	Unstratified	**0.81 (0.69, 0.94)**	**0.0052**	0.36	0.0	rs4648328		
ALDH2	GG	1.23 (0.97, 1.56)	0.0809	0.91	0.0	rs671		
C/T	GA	0.79 (0.62, 0.99)	0.0430	0.31	4.9	joint	-1.33 (-2.60, -0.07)	0.0391
**rs73550818**	Unstratified	0.99 (0.89, 1.10)	0.8005	0.60	0.0	rs73550818		
GOT2	GG	1.04 (0.85, 1.27)	0.7120	0.64	0.0	rs671		
C/A	GA	0.94 (0.82, 1.07)	0.3361	0.85	0.0	joint	-0.31 (-1.03, 0.41)	0.3985

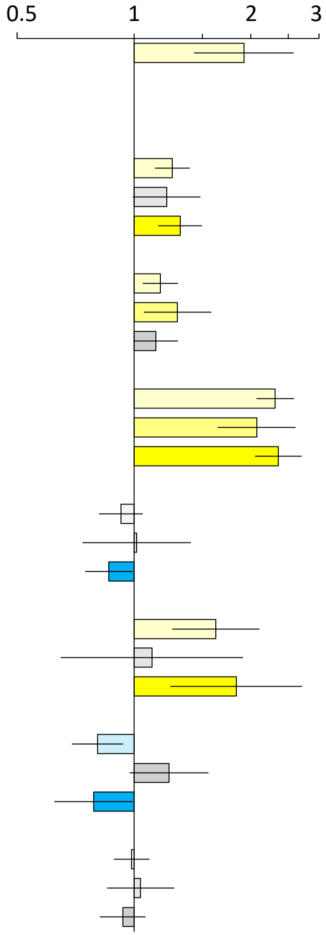

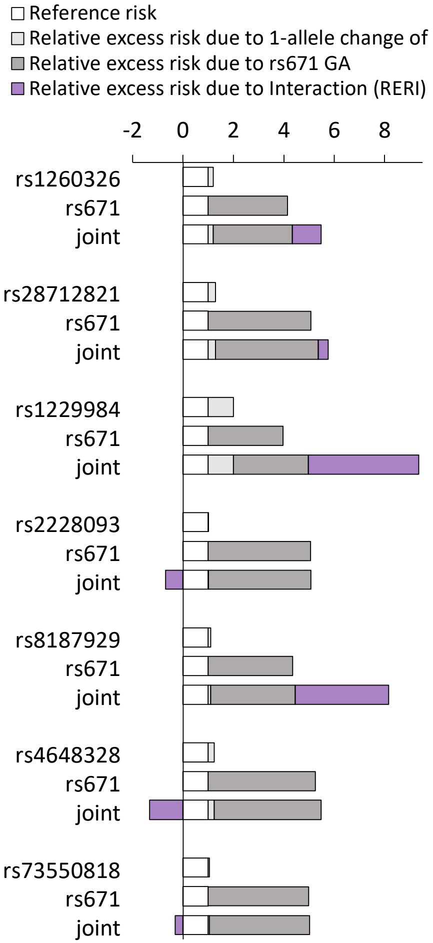

ORs for esophageal cancer per 1-allele change in eight SNPs on three different subject groups (the entire population [Unstratified], subjects with the rs671 GG genotype only [GG], and subjects with the rs671 GA genotype only [GA]) were calculated by a random effects model by pooling study-specific OR adjusted for sex, age, the first 10 principal components (for the BBJ Study), and study version (for the HERPACC Study). Stacked bar charts on assessment of additive interaction are presented as ORs partitioned into relative excess risks due to 1-allele change of each SNP other than rs671 (light gray), rs671 (GA vs. GG) (gray), and their interaction (RERI) (purple). Statistical significance was set at the Bonferroni corrected threshold of P < 0.05/8 (= 0.00625) and suggestive significance was set at P <0.05. RERI was considered to achieve suggestive significance (P < 0.05) when its confidence interval did not include 0. Estimates in bold show statistical significance with Bonferroni correction (P < 0.00625). The number of cases with squamous cell carcinoma were 636 (the HERPACC Study) and 348 (the BBJ Study). SNP, single nucleotide polymorphism; Ref, reference allele; Alt, alternative allele; OR, odds ratio; CI, confidence interval; Het*P*, P value from test of heterogeneity; BBJ, BioBank Japan; HERPACC, Hospital-based Epidemiologic Research Program at Aichi Cancer Center.

Fig. S16. Scheme of PRS analysis

We constructed three types of PRS for daily alcohol intake using the summary statistics of the unstratified ($PRS_{unstratified}$), rs671 GG-only (PRS_{GG}), and rs671 GA-only (PRS_{GA}) GWAS meta-analyses, based on the respective target dataset consisting of all 16,408 individuals with rs671 GG, GA, or AA genotypes, 9,124 wild-type homozygotes (GG), and 6,134 heterozygotes (GA). We then assessed how well $PRS_{unstratified}$, PRS_{GG}, and PRS_{GA} predict the residuals of $\log_2$ (daily alcohol intake + 1) on the validation dataset within 8,204 individuals, 4,562 wild-type homozygotes, and 3,067 heterozygotes, respectively. We further constructed a combined score of rs671, PRS_{GG}, and PRS_{GA} using 15,258 individuals with either rs671 GG or GA genotypes in the target dataset and assessed its predictive performance using 7,629 individuals with either rs671 GG or GA genotypes in the validation dataset.

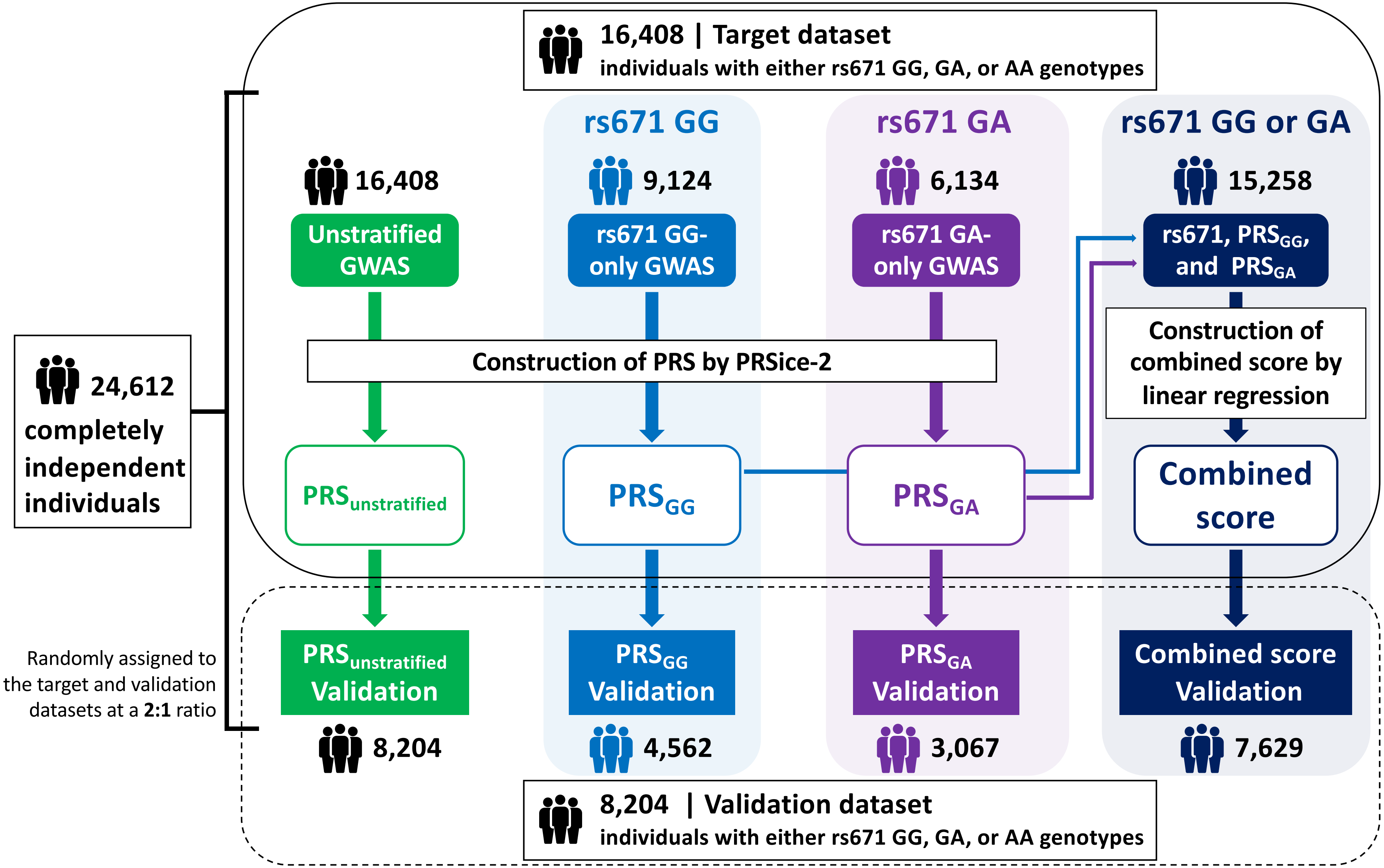

Fig. S17. Comparison of predictive performances (R^2 [%]) of rs671 alone, $PRS_{unstratified}$, and a combined score of rs671, PRS_{GG}, and PRS_{GA}

The gray, green, and dark blue bars reflect the predictive powers of rs671 alone, $PRS_{unstratified}$, and a combined score of rs671, PRS_{GG}, and PRS_{GA}, respectively. The predictive powers of rs671 alone and $PRS_{unstratified}$ obtained in all 8,204 individuals with either rs671 GG, GA, or AA genotypes in the validation dataset are shown on the left side, whereas those of rs671 alone, $PRS_{unstratified}$, and a combined score of rs671, PRS_{GG}, and PRS_{GA} in 7,629 individuals with either rs671 GG or GA genotypes in the validation data set are shown on the right side.

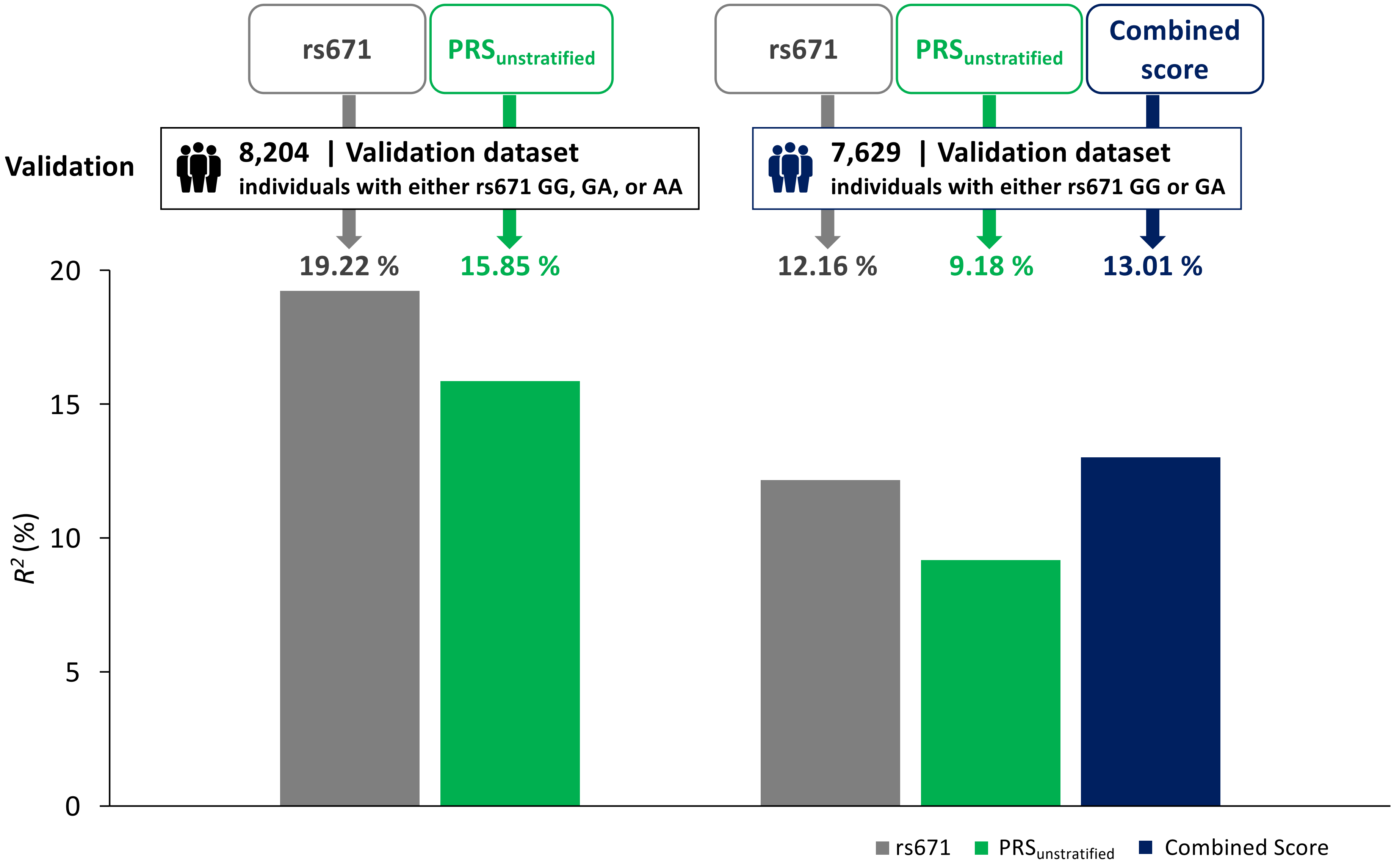

Fig. S18. Overview of this study

Summary of the analyses and findings of this study.

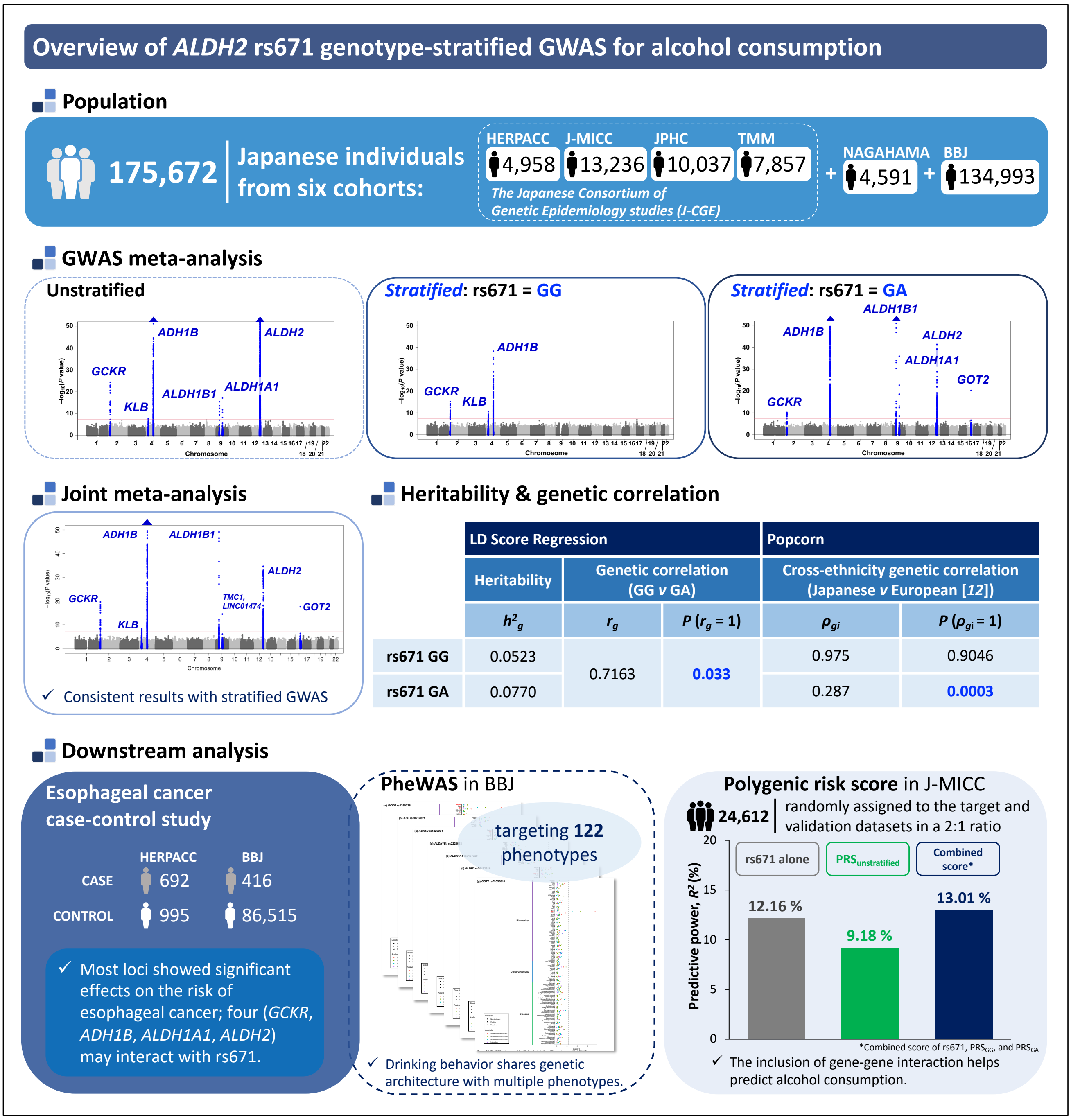

Fig. S19. PheWAS of the seven identified variants in the rs671 genotype-stratified GWAS
This figure shows PheWAS results of (A) *GCKR* rs1260326, (B) *KLB* rs28712821, (C) *ADH1B* rs1229984, (D) *ALDH1B1* rs2228093, (E) *ALDH1A1* rs8187929, (F) *ALDH2* rs79463616, and (G) *GOT2* rs73550818 for the entire population (Unstratified, red); for subjects with the rs671 GG genotype only (GG, green); for subjects with the rs671 GA genotype only (GA, blue); and interaction with rs671 (purple). The observed $-\log_{10}(P$ value) and 122 phenotypes are plotted on the x-axis and y-axis, respectively. The dashed gray line indicates the significance level ($\alpha = 0.05/122$). The phenotype satisfying the significance threshold ($P < 0.05/122$) in the interaction analyses is shown in red. The direction of significant associations is indicated using an arrowhead pointing either left (negative) or right (positive). Abbreviations are listed in table S24. Phenotypes are marked with a "+" on the right side of the graph if they are significant in each analysis. Among these, phenotypes that are significant in only one of these analyses, either the rs671 GG- or GA-only, are highlighted with a square. Phenotypes that demonstrated a significant interaction with rs671 among these are emphasized in red font.

(A) *GCKR* rs1260326

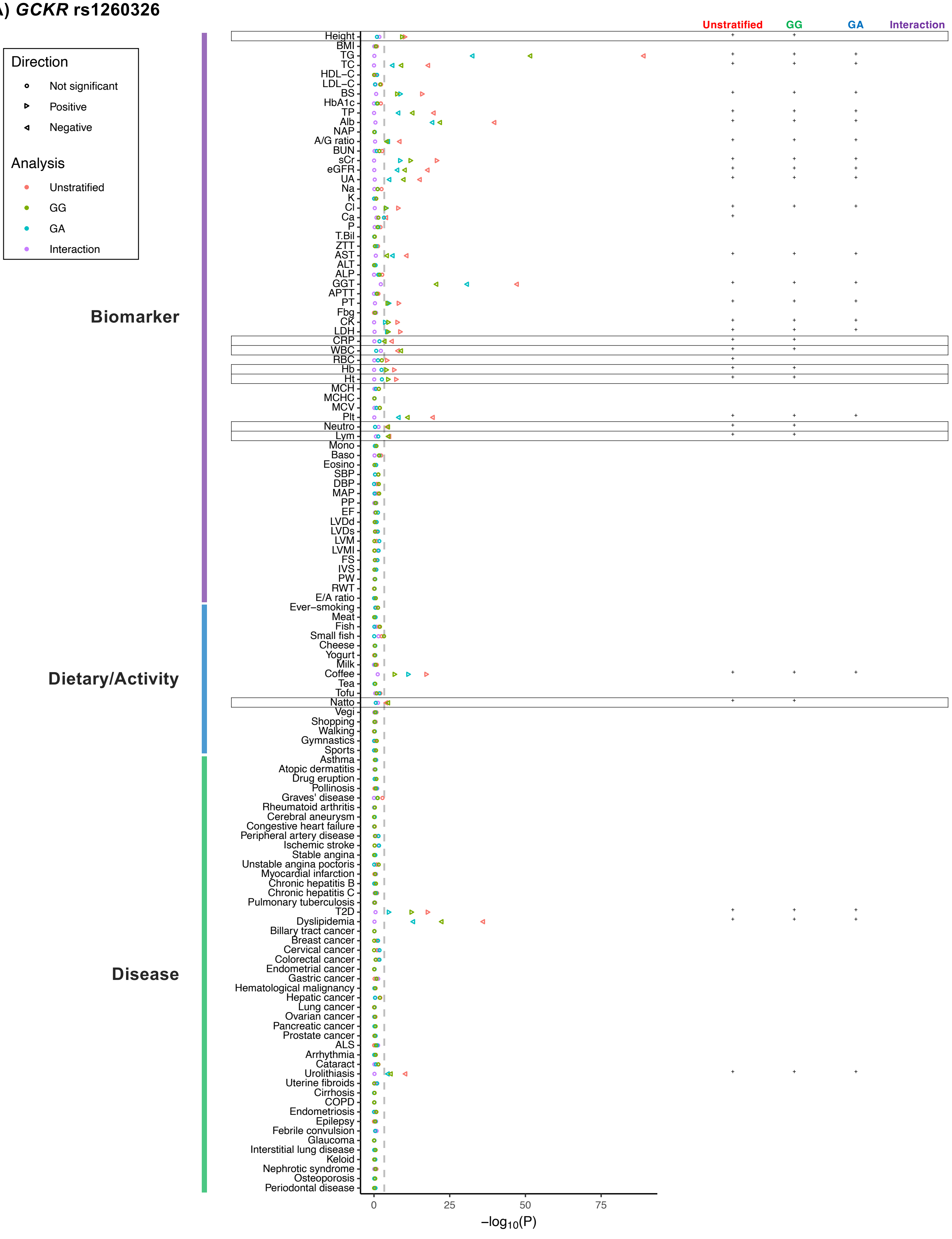

Fig. S19. PheWAS of the seven identified variants in the rs671 genotype-stratified GWAS

This figure shows PheWAS results of (A) *GCKR* rs1260326, (B) *KLB* rs28712821, (C) *ADH1B* rs1229984, (D) *ALDH1B1* rs2228093, (E) *ALDH1A1* rs8187929, (F) *ALDH2* rs79463616, and (G) *GOT2* rs73550818 for the entire population (Unstratified, red); for subjects with the rs671 GG genotype only (GG, green); for subjects with the rs671 GA genotype only (GA, blue); and interaction with rs671 (purple). The observed $-\log_{10}(P$ value) and 122 phenotypes are plotted on the x-axis and y-axis, respectively. The dashed gray line indicates the significance level ($\alpha = 0.05/122$). The phenotype satisfying the significance threshold ($P < 0.05/122$) in the interaction analyses is shown in red. The direction of significant associations is indicated using an arrowhead pointing either left (negative) or right (positive). Abbreviations are listed in table S24. Phenotypes are marked with a "+" on the right side of the graph if they are significant in each analysis. Among these, phenotypes that are significant in only one of these analyses, either the rs671 GG- or GA-only, are highlighted with a square. Phenotypes that demonstrated a significant interaction with rs671 among these are emphasized in red font.

(B) *KLB* rs28712821

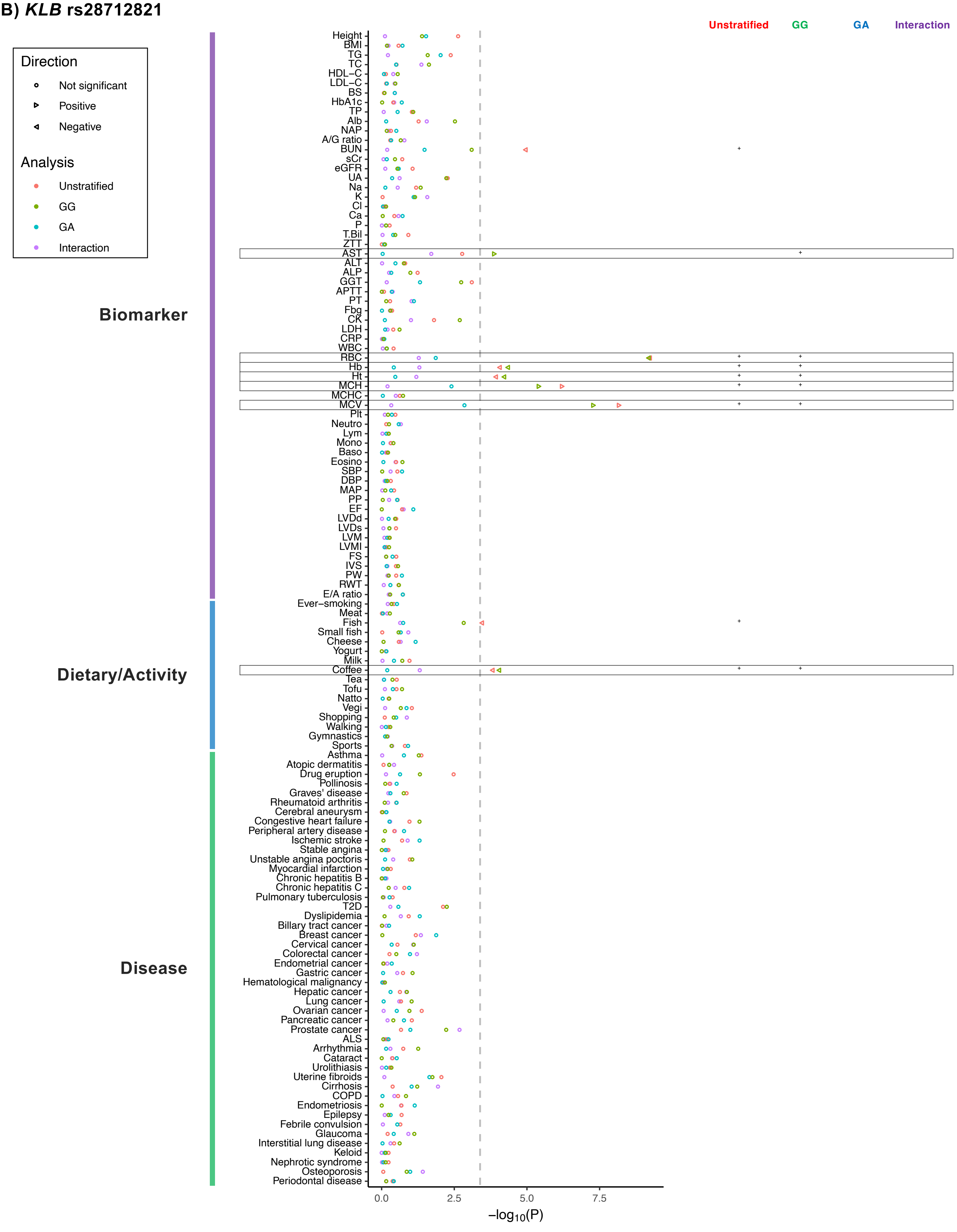

Fig. S19. PheWAS of the seven identified variants in the rs671 genotype-stratified GWAS

This figure shows PheWAS results of (A) *GCKR* rs1260326, (B) *KLB* rs28712821, (C) *ADH1B* rs1229984, (D) *ALDH1B1* rs2228093, (E) *ALDH1A1* rs8187929, (F) *ALDH2* rs79463616, and (G) *GOT2* rs73550818 for the entire population (Unstratified, red); for subjects with the rs671 GG genotype only (GG, green); for subjects with the rs671 GA genotype only (GA, blue); and interaction with rs671 (purple). The observed $-\log_{10}(P$ value) and 122 phenotypes are plotted on the x-axis and y-axis, respectively. The dashed gray line indicates the significance level ($\alpha = 0.05/122$). The phenotype satisfying the significance threshold ($P < 0.05/122$) in the interaction analyses is shown in red. The direction of significant associations is indicated using an arrowhead pointing either left (negative) or right (positive). Abbreviations are listed in table S24. Phenotypes are marked with a "+" on the right side of the graph if they are significant in each analysis. Among these, phenotypes that are significant in only one of these analyses, either the rs671 GG- or GA-only, are highlighted with a square. Phenotypes that demonstrated a significant interaction with rs671 among these are emphasized in red font.

(C) *ADH1B* rs1229984

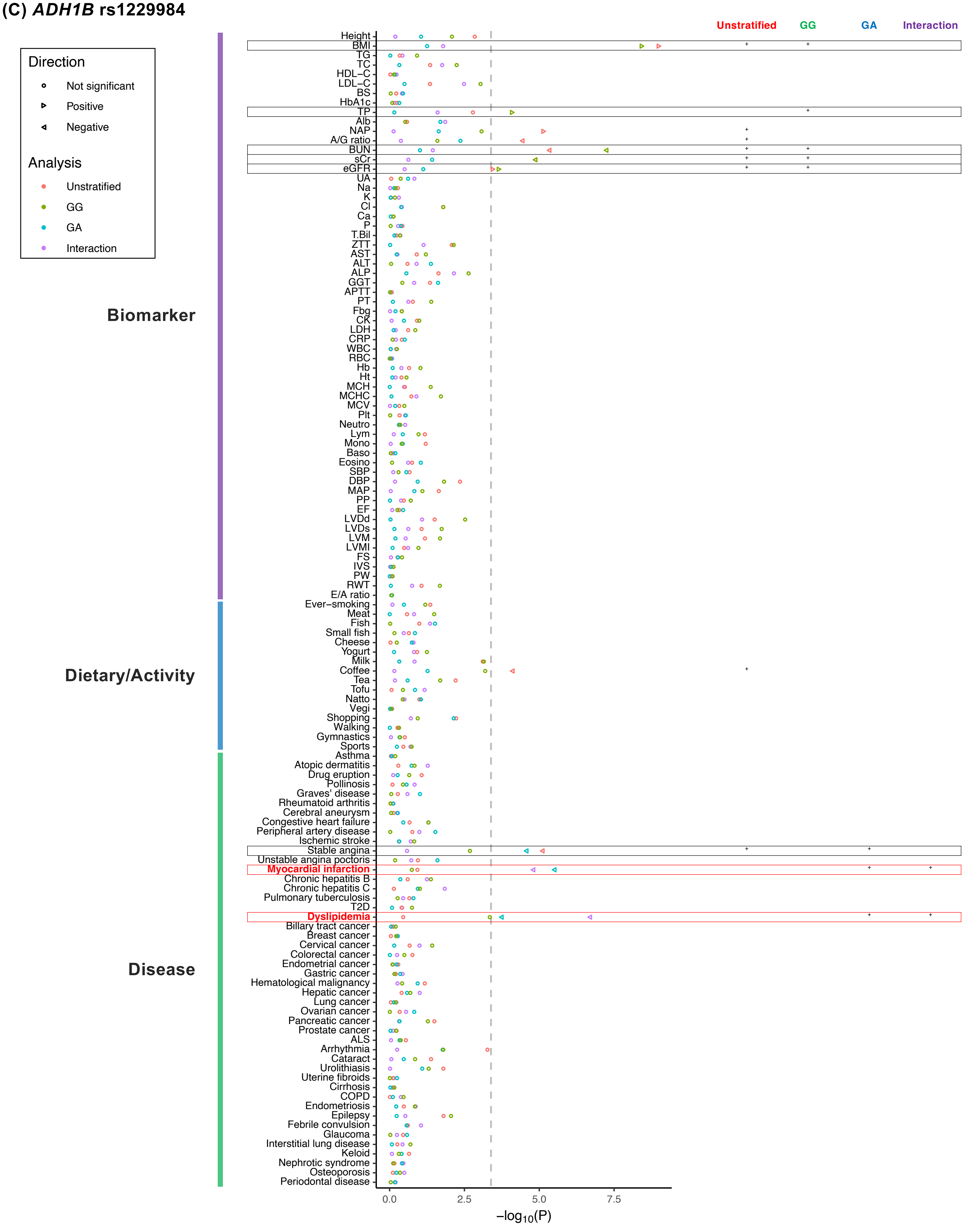

Fig. S19. PheWAS of the seven identified variants in the rs671 genotype-stratified GWAS

This figure shows PheWAS results of (A) *GCKR* rs1260326, (B) *KLB* rs28712821, (C) *ADH1B* rs1229984, (D) *ALDH1B1* rs2228093, (E) *ALDH1A1* rs8187929, (F) *ALDH2* rs79463616, and (G) *GOT2* rs73550818 for the entire population (Unstratified, red); for subjects with the rs671 GG genotype only (GG, green); for subjects with the rs671 GA genotype only (GA, blue); and interaction with rs671 (purple). The observed $-\log_{10}(P$ value) and 122 phenotypes are plotted on the x-axis and y-axis, respectively. The dashed gray line indicates the significance level (α = 0.05/122). The phenotype satisfying the significance threshold ($P < 0.05/122$) in the interaction analyses is shown in red. The direction of significant associations is indicated using an arrowhead pointing either left (negative) or right (positive). Abbreviations are listed in table S24. Phenotypes are marked with a "+" on the right side of the graph if they are significant in each analysis. Among these, phenotypes that are significant in only one of these analyses, either the rs671 GG- or GA-only, are highlighted with a square. Phenotypes that demonstrated a significant interaction with rs671 among these are emphasized in red font.

(D) *ALDH1B1* rs2228093

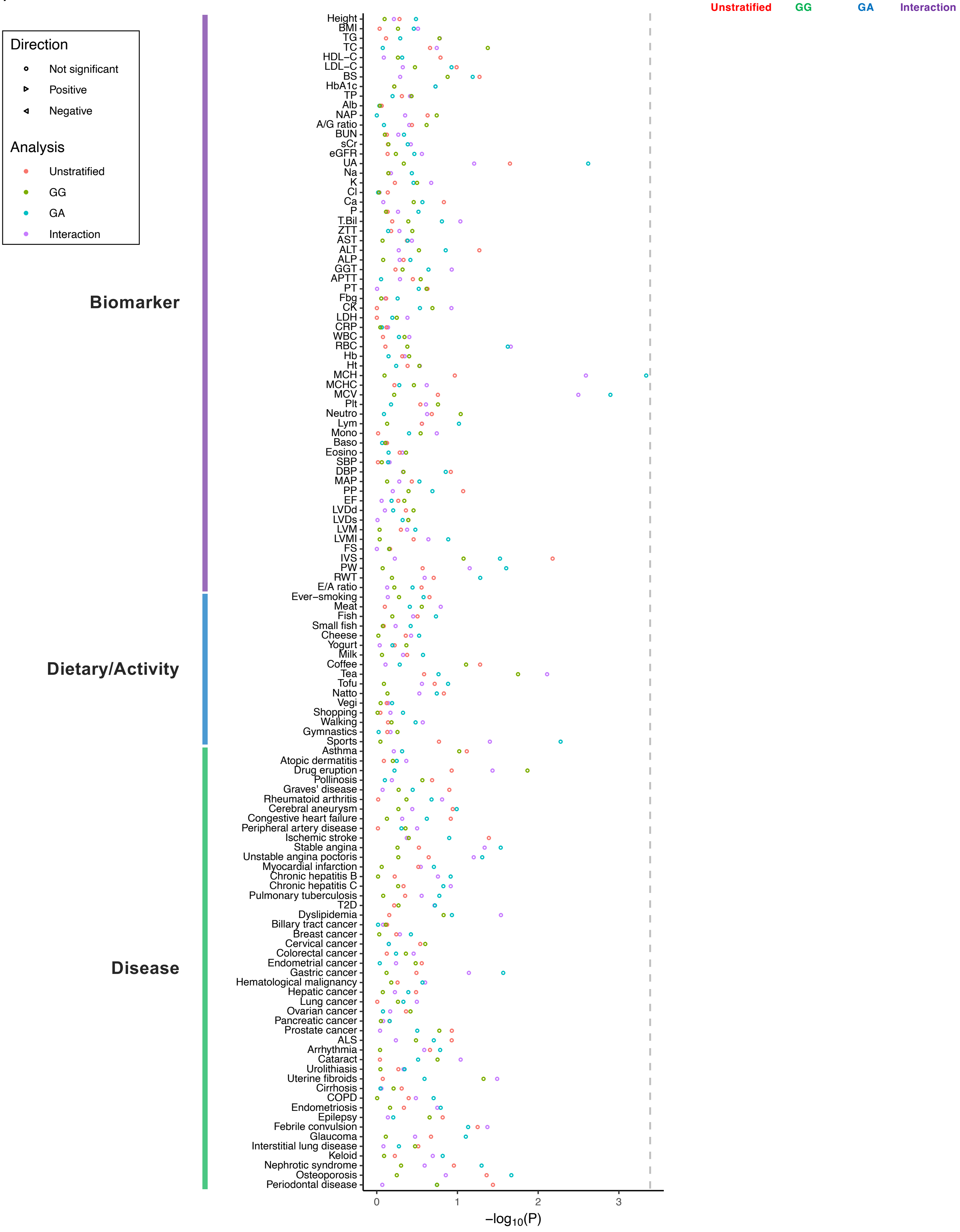

Fig. S19. PheWAS of the seven identified variants in the rs671 genotype-stratified GWAS

This figure shows PheWAS results of (A) *GCKR* rs1260326, (B) *KLB* rs28712821, (C) *ADH1B* rs1229984, (D) *ALDH1B1* rs2228093, (E) *ALDH1A1* rs8187929, (F) *ALDH2* rs79463616, and (G) *GOT2* rs73550818 for the entire population (Unstratified, red); for subjects with the rs671 GG genotype only (GG, green); for subjects with the rs671 GA genotype only (GA, blue); and interaction with rs671 (purple). The observed $-\log_{10}(P$ value) and 122 phenotypes are plotted on the x-axis and y-axis, respectively. The dashed gray line indicates the significance level ($\alpha = 0.05/122$). The phenotype satisfying the significance threshold ($P < 0.05/122$) in the interaction analyses is shown in red. The direction of significant associations is indicated using an arrowhead pointing either left (negative) or right (positive). Abbreviations are listed in table S24. Phenotypes are marked with a "+" on the right side of the graph if they are significant in each analysis. Among these, phenotypes that are significant in only one of these analyses, either the rs671 GG- or GA-only, are highlighted with a square. Phenotypes that demonstrated a significant interaction with rs671 among these are emphasized in red font.

(E) *ALDH1A1* rs8187929

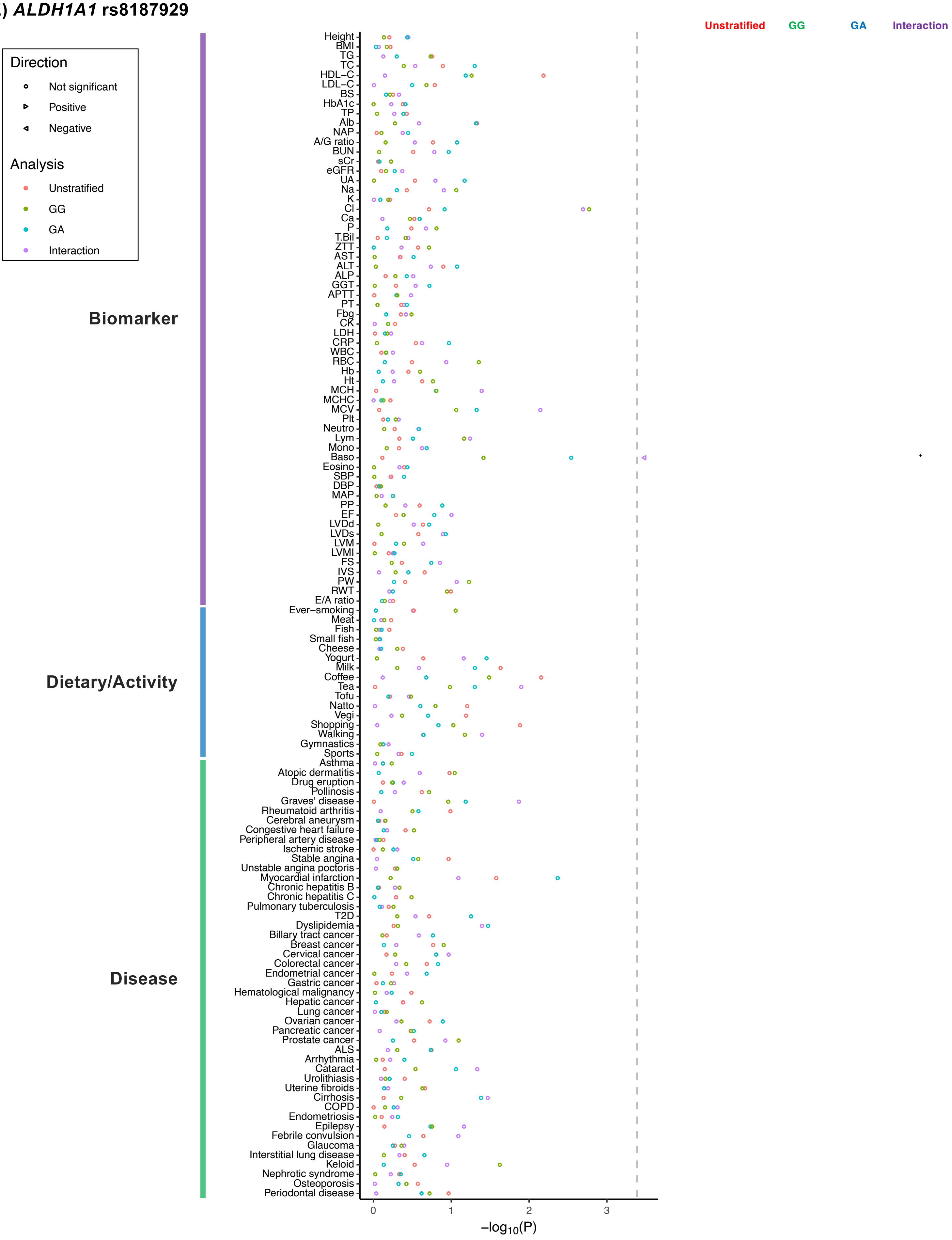

Fig. S19. PheWAS of the seven identified variants in the rs671 genotype-stratified GWAS

This figure shows PheWAS results of (A) *GCKR* rs1260326, (B) *KLB* rs28712821, (C) *ADH1B* rs1229984, (D) *ALDH1B1* rs2228093, (E) *ALDH1A1* rs8187929, (F) *ALDH2* rs79463616, and (G) *GOT2* rs73550818 for the entire population (Unstratified, red); for subjects with the rs671 GG genotype only (GG, green); for subjects with the rs671 GA genotype only (GA, blue); and interaction with rs671 (purple). The observed $-\log_{10}(P$ value) and 122 phenotypes are plotted on the x-axis and y-axis, respectively. The dashed gray line indicates the significance level ($\alpha = 0.05/122$). The phenotype satisfying the significance threshold ($P < 0.05/122$) in the interaction analyses is shown in red. The direction of significant associations is indicated using an arrowhead pointing either left (negative) or right (positive). Abbreviations are listed in table S24. Phenotypes are marked with a "+" on the right side of the graph if they are significant in each analysis. Among these, phenotypes that are significant in only one of these analyses, either the rs671 GG- or GA-only, are highlighted with a square. Phenotypes that demonstrated a significant interaction with rs671 among these are emphasized in red font.

(F) *ALDH2* rs79463616

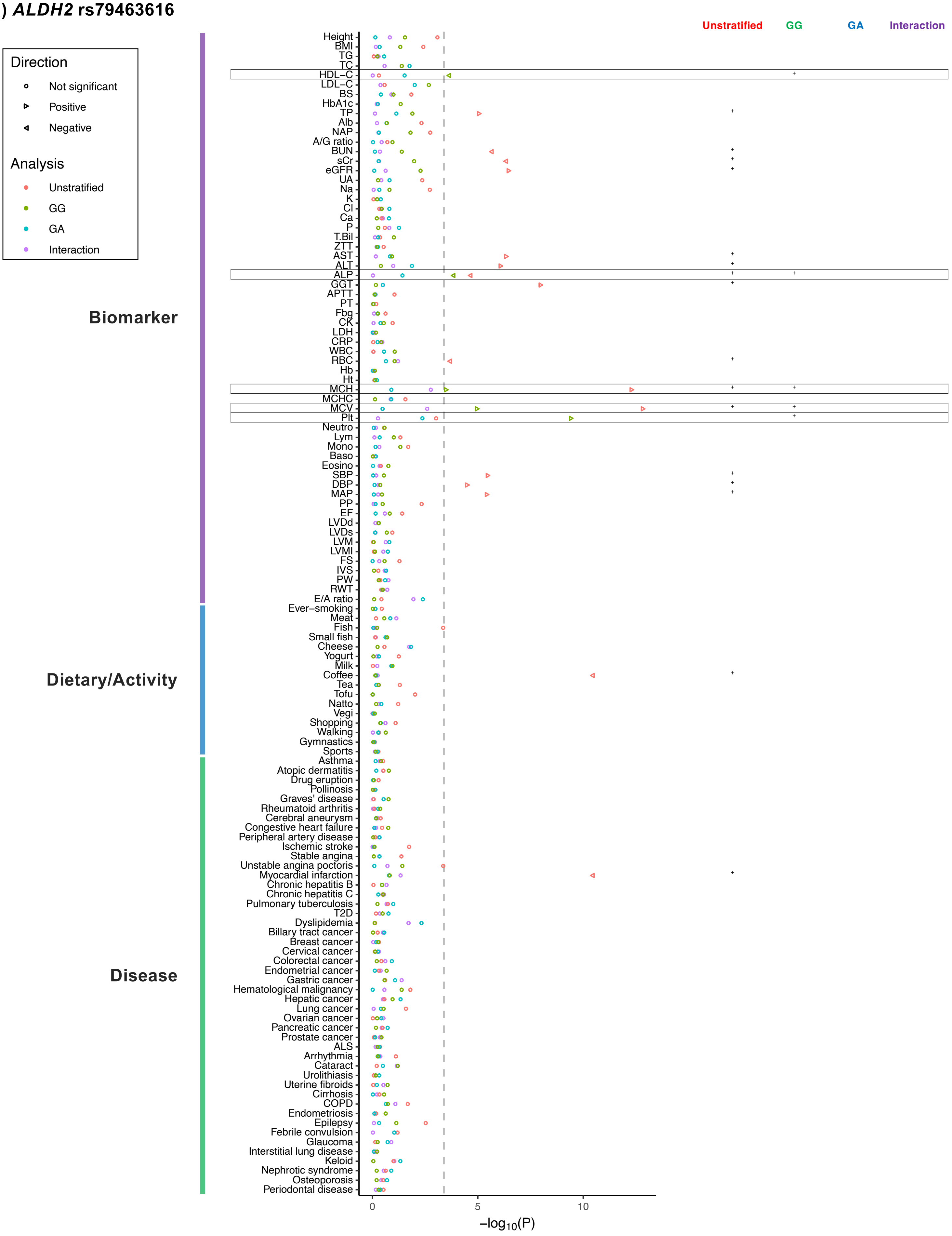

Fig. S19. PheWAS of the seven identified variants in the rs671 genotype-stratified GWAS

This figure shows PheWAS results of (A) *GCKR* rs1260326, (B) *KLB* rs28712821, (C) *ADH1B* rs1229984, (D) *ALDH1B1* rs2228093, (E) *ALDH1A1* rs8187929, (F) *ALDH2* rs79463616, and (G) *GOT2* rs73550818 for the entire population (Unstratified, red); for subjects with the rs671 GG genotype only (GG, green); for subjects with the rs671 GA genotype only (GA, blue); and interaction with rs671 (purple). The observed $-\log_{10}(P$ value) and 122 phenotypes are plotted on the x-axis and y-axis, respectively. The dashed gray line indicates the significance level ($\alpha = 0.05/122$). The phenotype satisfying the significance threshold ($P < 0.05/122$) in the interaction analyses is shown in red. The direction of significant associations is indicated using an arrowhead pointing either left (negative) or right (positive). Abbreviations are listed in table S24. Phenotypes are marked with a "+" on the right side of the graph if they are significant in each analysis. Among these, phenotypes that are significant in only one of these analyses, either the rs671 GG- or GA-only, are highlighted with a square. Phenotypes that demonstrated a significant interaction with rs671 among these are emphasized in red font.

(G) *GOT2* rs73550818

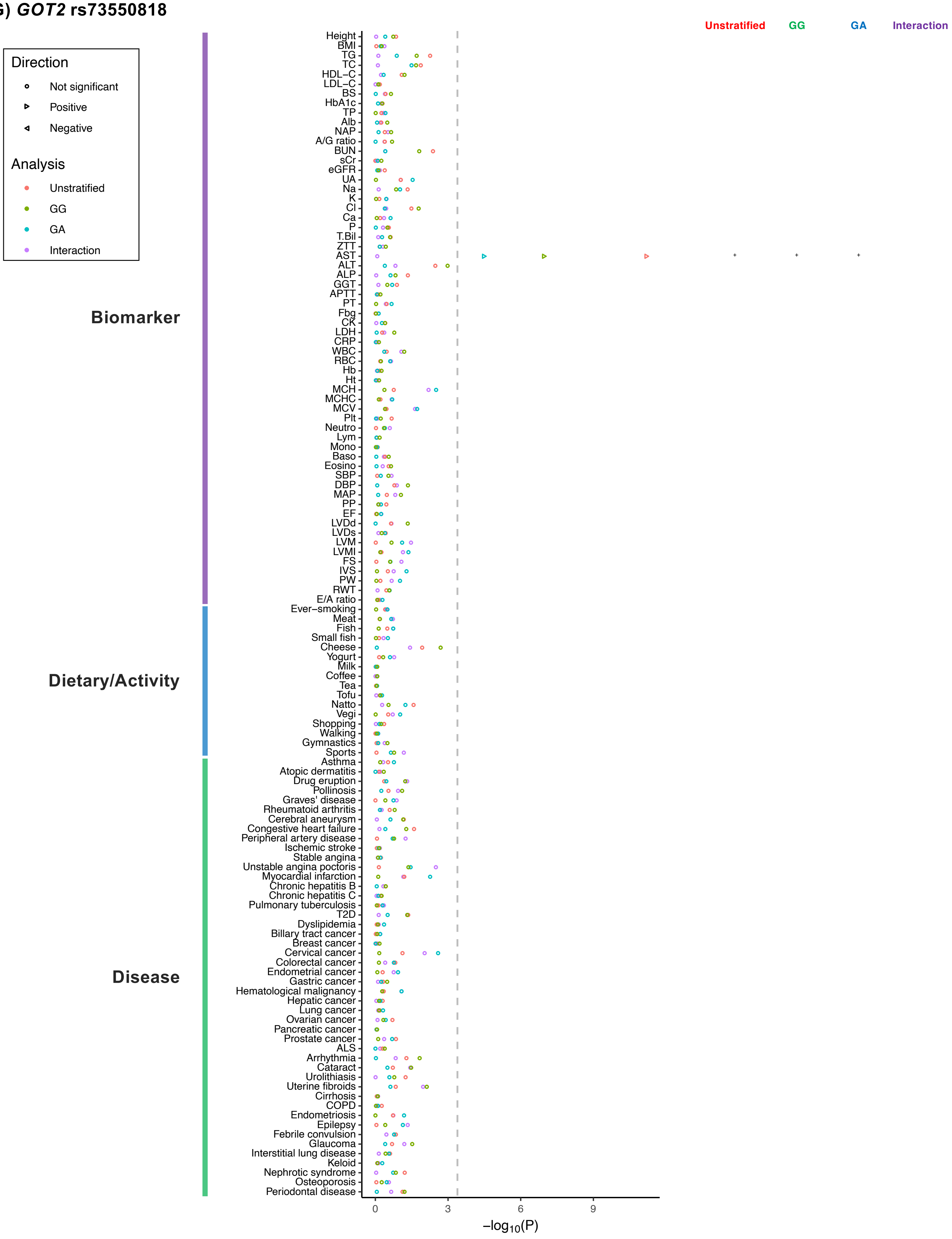

Supplementary Tables S1-S25 (External Excel file)

Table S1. Characteristics of study participants
Table S2. Cohort-specific information on genotyping, imputation and association testing
Table S3. Number of subjects with each genotype of rs671
Table S4. Functional annotation results and allele frequencies across different ancestries for the lead SNPs
Table S5. Allele frequencies across different East Asian ancestries for the lead SNPs
Table S6. Genomic loci reaching genome-wide significance in the JMA
Table S7. Haplotypes of rs79463616-rs671 in Japanese samples
Table S8. Haplotypes of SNPs at 12q24.12 in Japanese samples
Table S9. The 70 SNPs at 12q24.12 which showed P-value < 1×10^{30} for daily alcohol intake in rs671 heterozygotes (GA)
Table S10. Results from conditional analysis for the genome-wide significant loci for daily alcohol intake in rs671 heterozygotes (GA)
Table S11. Haplotypes of rs1229984-rs13125415 and rs2228093-rs3043 in 1000 genomes JPT (n = 104)
Table S12. LD score regression results
Table S13. Associations of previously reported loci in European populations
Table S14. Lookup of our GWAS-significant variants in the GSCAN data Table S15. Heritability and cross-ethnicity genetic correlation
Table S16. Lead SNPs in ALDH2 and GOT2 regions found to be eQTLs
Table S17. Characteristics of cases and controls in esophageal cancer case-control study
Table S18. Study-specific information on alterative allele frequency and imputation quality score of each SNP in an esophageal cancer case-control study

Table S19. Summary estimates of the ORs for each SNP (1-allele change), ORs for rs671 (GA vs. GG), and joint ORs for each SNP and rs671 and results of multiplicative interaction tests
Table S20. Results of two sensitivity analyses in an esophageal cancer case-control study: impact of identified variants on esophageal cancer risk and assessment of additive interaction of each SNP (1-allele change) with rs671 (GG vs. GA)
Table S21. Predictive performances of polygenic risk scores for daily alcohol intake
Table S22. Comparison of predictive performances of rs671 and PRSunstratified in individuals with either the rs671 GG, GA, or AA genotypes
Table S23. Comparison of predictive performances of rs671, PRSunstratified, and combined score in individuals with either the rs671 GG or GA genotypes
Table S24. Abbreviation and description of each phenotype in PheWAS
Table S25. PheWAS results within the BBJ Study targeting 122 phenotypes

REFERENCES AND NOTES

1. GBD 2016 Alcohol Collaborators, Alcohol use and burden for 195 countries and territories, 1990–2016: A systematic analysis for the Global Burden of Disease Study 2016. *Lancet* **392**, 1015–1035 (2018).

2. A. M. Wood, S. Kaptoge, A. S. Butterworth, P. Willeit, S. Warnakula, T. Bolton, E. Paige, D. S. Paul, M. Sweeting, S. Burgess, S. Bell, W. Astle, D. Stevens, A. Koulman, R. M. Selmer, W. M. M. Verschuren, S. Sato, I. Njølstad, M. Woodward, V. Salomaa, B. G. Nordestgaard, B. B. Yeap, A. Fletcher, O. Melander, L. H. Kuller, B. Balkau, M. Marmot, W. Koenig, E. Casiglia, C. Cooper, V. Arndt, O. H. Franco, P. Wennberg, J. Gallacher, A. G. de la Cámara, H. Völzke, C. C. Dahm, C. E. Dale, M. M. Bergmann, C. J. Crespo, Y. T. van der Schouw, R. Kaaks, L. A. Simons, P. Lagiou, J. D. Schoufour, J. M. A. Boer, T. J. Key, B. Rodriguez, C. Moreno-Iribas, K. W. Davidson, J. O. Taylor, C. Sacerdote, R. B. Wallace, J. R. Quiros, R. Tumino, D. G. Blazer II, A. Linneberg, M. Daimon, S. Panico, B. Howard, G. Skeie, T. Strandberg, E. Weiderpass, P. J. Nietert, B. M. Psaty, D. Kromhout, E. Salamanca-Fernandez, S. Kiechl, H. M. Krumholz, S. Grioni, D. Palli, J. M. Huerta, J. Price, J. Sundström, L. Arriola, H. Arima, R. C. Travis, D. B. Panagiotakos, A. Karakatsani, A. Trichopoulou, T. Kühn, D. E. Grobbee, E. Barrett-Connor, N. van Schoor, H. Boeing, K. Overvad, J. Kauhanen, N. Wareham, C. Langenberg, N. Forouhi, M. Wennberg, J.-P. Després, M. Cushman, J. A. Cooper, C. J. Rodriguez, M. Sakurai, J. E. Shaw, M. Knuiman, T. Voortman, C. Meisinger, A. Tjønneland, H. Brenner, L. Palmieri, J. Dallongeville, E. J. Brunner, G. Assmann, M. Trevisan, R. F. Gillum, I. Ford, N. Sattar, M. Lazo, S. G. Thompson, P. Ferrari, D. A. Leon, G. D. Smith, R. Peto, R. Jackson, E. Banks, E. D. Angelantonio, J. Danesh, Emerging Risk Factors Collaboration/EPIC-CVD/UK Biobank Alcohol Study Group, Risk thresholds for alcohol consumption: Combined analysis of individual-participant data for 599 912 current drinkers in 83 prospective studies. *Lancet* **391**, 1513–1523 (2018).

3. J. Kaprio, M. Koskenvuo, H. Langinvainio, K. Romanov, S. Sarna, R. J. Rose, Genetic influences on use and abuse of alcohol: A study of 5638 adult Finnish twin brothers. *Alcohol. Clin. Exp. Res.* **11**, 349–356 (1987).

4. A. C. Heath, J. Meyer, R. Jardine, N. G. Martin, The inheritance of alcohol consumption patterns in a general population twin sample: II. Determinants of consumption frequency and quantity consumed. *J. Stud. Alcohol* **52**, 425–433 (1991).

5. I. Baik, N. H. Cho, S. H. Kim, B. G. Han, C. Shin, Genome-wide association studies identify genetic loci related to alcohol consumption in Korean men. *Am. J. Clin. Nutr.* **93**, 809–816 (2011).

6. G. Schumann, L. J. Coin, A. Lourdusamy, P. Charoen, K. H. Berger, D. Stacey, S. Desrivieres, F. A. Aliev, A. A. Khan, N. Amin, Y. S. Aulchenko, G. Bakalkin, S. J. Bakker, B. Balkau, J. W. Beulens, A. Bilbao, R. A. de Boer, D. Beury, M. L. Bots, E. J. Breetvelt, S. Cauchi, C. Cavalcanti-Proenca, J. C. Chambers, T. K. Clarke, N. Dahmen, E. J. de Geus, D. Dick, F. Ducci, A. Easton, H. J. Edenberg, T. Esko, A. Fernandez-Medarde, T. Foroud, N. B. Freimer, J. A. Girault, D. E. Grobbee, S. Guarrera, D. F. Gudbjartsson, A. L. Hartikainen, A. C. Heath, V. Hesselbrock, A. Hofman, J. J. Hottenga, M. K. Isohanni, J. Kaprio, K. T. Khaw, B. Kuehnel, J. Laitinen, S. Lobbens, J. Luan, M. Mangino, M. Maroteaux, G. Matullo, M. I. McCarthy, C. Mueller, G. Navis, M. E. Numans, A. Nunez, D. R. Nyholt, C. N. Onland-Moret, B. A. Oostra, P. F. O'Reilly, M. Palkovits, B. W. Penninx, S. Polidoro, A. Pouta, I. Prokopenko, F. Ricceri, E. Santos, J. H. Smit, N. Soranzo, K. Song, U. Sovio, M. Stumvoll, I. Surakk, T. E. Thorgeirsson, U. Thorsteinsdottir, C. Troakes, T. Tyrfingsson, A. Tonjes, C. S. Uiterwaal, A. G. Uitterlinden, P. van der Harst, Y. T. van der Schouw, O. Staehlin, N. Vogelzangs, P. Vollenweider, G. Waeber, N. J. Wareham, D. M. Waterworth, J. B. Whitfield, E. H. Wichmann, G. Willemsen, J. C. Witteman, X. Yuan, G. Zhai, J. H. Zhao, W. Zhang, N. G. Martin, A. Metspalu, A. Doering, J. Scott, T. D. Spector, R. J. Loos, D. I. Boomsma, V. Mooser, L. Peltonen, K. Stefansson, C. M. van Duijn, P. Vineis, W. H. Sommer, J. S. Kooner, R. Spanagel, U. A. Heberlein, M. R. Jarvelin, P. Elliott, Genome-wide association and genetic functional studies identify autism susceptibility candidate 2 gene (AUTS2) in the regulation of alcohol consumption. *Proc. Natl. Acad. Sci. U.S.A.* **108**, 7119–7124 (2011).

7. F. Takeuchi, M. Isono, T. Nabika, T. Katsuya, T. Sugiyama, S. Yamaguchi, S. Kobayashi, T. Ogihara, Y. Yamori, A. Fujioka, N. Kato, Confirmation of ALDH2 as a major locus of drinking

behavior and of its variants regulating multiple metabolic phenotypes in a Japanese population. *Circ. J.* **75**, 911–918 (2011).

8. X. Yang, X. Lu, L. Wang, S. Chen, J. Li, J. Cao, J. Chen, Y. Hao, Y. Li, L. Zhao, H. Li, D. Liu, L. Wang, F. Lu, C. Shen, L. Yu, X. Wu, Q. Zhao, X. Ji, D. Guo, X. Peng, J. Huang, D. Gu, Common variants at 12q24 are associated with drinking behavior in Han Chinese. *Am. J. Clin. Nutr.* **97**, 545–551 (2013).

9. G. Schumann, C. Liu, P. O'Reilly, H. Gao, P. Song, B. Xu, B. Ruggeri, N. Amin, T. Jia, S. Preis, M. S. Lepe, S. Akira, C. Barbieri, S. Baumeister, S. Cauchi, T.-K. Clarke, S. Enroth, K. Fischer, J. Hällfors, S. E. Harris, S. Hieber, E. Hofer, J.-J. Hottenga, Å. Johansson, P. K. Joshi, N. Kaartinen, J. Laitinen, R. Lemaitre, A. Loukola, J. Luan, L.-P. Lyytikäinen, M. Mangino, A. Manichaikul, H. Mbarek, Y. Milaneschi, A. Moayyeri, K. Mukamal, C. Nelson, J. Nettleton, E. Partinen, R. Rawal, A. Robino, L. Rose, C. Sala, T. Satoh, R. Schmidt, K. Schraut, R. Scott, A. V. Smith, J. M. Starr, A. Teumer, S. Trompet, A. G. Uitterlinden, C. Venturini, A.-C. Vergnaud, N. Verweij, V. Vitart, D. Vuckovic, J. Wedenoja, L. Yengo, B. Yu, W. Zhang, J. H. Zhao, D. I. Boomsma, J. Chambers, D. I. Chasman, T. Daniela, E. de Geus, I. Deary, J. G. Eriksson, T. Esko, V. Eulenburg, O. H. Franco, P. Froguel, C. Gieger, H. J. Grabe, V. Gudnason, U. Gyllensten, T. B. Harris, A.-L. Hartikainen, A. C. Heath, L. Hocking, A. Hofman, C. Huth, M.-R. Jarvelin, J. W. Jukema, J. Kaprio, J. S. Kooner, Z. Kutalik, J. Lahti, C. Langenberg, T. Lehtimäki, Y. Liu, P. A. F. Madden, N. Martin, A. Morrison, B. Penninx, N. Pirastu, B. Psaty, O. Raitakari, P. Ridker, R. Rose, J. I. Rotter, N. J. Samani, H. Schmidt, T. D. Spector, D. Stott, D. Strachan, I. Tzoulaki, P. van der Harst, C. M. van Duijn, P. Marques-Vidal, P. Vollenweider, N. J. Wareham, J. B. Whitfield, J. Wilson, B. Wolffenbuttel, G. Bakalkin, E. Evangelou, Y. Liu, K. M. Rice, S. Desrivières, S. A. Kliewer, D. J. Mangelsdorf, C. P. Müller, D. Levy, P. Elliott, KLB is associated with alcohol drinking, and its gene product beta-Klotho is necessary for FGF21 regulation of alcohol preference. *Proc. Natl. Acad. Sci. U.S.A.* **113**, 14372–14377 (2016).

10. T. K. Clarke, M. J. Adams, G. Davies, D. M. Howard, L. S. Hall, S. Padmanabhan, A. D. Murray, B. H. Smith, A. Campbell, C. Hayward, D. J. Porteous, I. J. Deary, A. M. McIntosh,

Genome-wide association study of alcohol consumption and genetic overlap with other health-related traits in UK Biobank (*N*=112 117). *Mol. Psychiatry* **22**, 1376–1384 (2017).

11. E. Jorgenson, K. K. Thai, T. J. Hoffmann, L. C. Sakoda, M. N. Kvale, Y. Banda, C. Schaefer, N. Risch, J. Mertens, C. Weisner, H. Choquet, Genetic contributors to variation in alcohol consumption vary by race/ethnicity in a large multi-ethnic genome-wide association study. *Mol. Psychiatry* **22**, 1359–1367 (2017).

12. M. Liu, Y. Jiang, R. Wedow, Y. Li, D. M. Brazel, F. Chen, G. Datta, J. Davila-Velderrain, D. M. Guire, C. Tian, X. Zhan, 23andMe Research Team, HUNT All-In Psychiatry, H. Choquet, A. R. Docherty, J. D. Faul, J. R. Foerster, L. G. Fritsche, M. E. Gabrielsen, S. D. Gordon, J. Haessler, J.-J. Hottenga, H. Huang, S.-K. Jang, P. R. Jansen, Y. Ling, R. Mägi, N. Matoba, G. M. Mahon, A. Mulas, V. Orrù, T. Palviainen, A. Pandit, G. W. Reginsson, A. H. Skogholt, J. A. Smith, A. E. Taylor, C. Turman, G. Willemsen, H. Young, K. A. Young, G. J. M. Zajac, W. Zhao, W. Zhou, G. Bjornsdottir, J. D. Boardman, M. Boehnke, D. I. Boomsma, C. Chen, F. Cucca, G. E. Davies, C. B. Eaton, M. A. Ehringer, T. Esko, E. Fiorillo, N. A. Gillespie, D. F. Gudbjartsson, T. Haller, K. M. Harris, A. C. Heath, J. K. Hewitt, I. B. Hickie, J. E. Hokanson, C. J. Hopfer, D. J. Hunter, W. G. Iacono, E. O. Johnson, Y. Kamatani, S. L. R. Kardia, M. C. Keller, M. Kellis, C. Kooperberg, P. Kraft, K. S. Krauter, M. Laakso, P. A. Lind, A. Loukola, S. M. Lutz, P. A. F. Madden, N. G. Martin, M. M. Gue, M. B. Mc Queen, S. E. Medland, A. Metspalu, K. L. Mohlke, J. B. Nielsen, Y. Okada, U. Peters, T. J. C. Polderman, D. Posthuma, A. P. Reiner, J. P. Rice, E. Rimm, R. J. Rose, V. Runarsdottir, M. C. Stallings, A. Stančáková, H. Stefansson, K. K. Thai, H. A. Tindle, T. Tyrfingsson, T. L. Wall, D. R. Weir, C. Weisner, J. B. Whitfield, B. S. Winsvold, J. Yin, L. Zuccolo, L. J. Bierut, K. Hveem, J. J. Lee, M. R. Munafò, N. L. Saccone, C. J. Willer, M. C. Cornelis, S. P. David, D. A. Hinds, E. Jorgenson, J. Kaprio, J. A. Stitzel, K. Stefansson, T. E. Thorgeirsson, G. Abecasis, D. J. Liu, S. Vrieze, Association studies of up to 1.2 million individuals yield new insights into the genetic etiology of tobacco and alcohol use. *Nat. Genet.* **51**, 237–244 (2019).

13. V. W. Zhong, A. Kuang, R. D. Danning, P. Kraft, R. M. van Dam, D. I. Chasman, M. C. Cornelis, A genome-wide association study of bitter and sweet beverage consumption. *Hum. Mol. Genet.* **28**, 2449–2457 (2019).

14. N. Matoba, M. Akiyama, K. Ishigaki, M. Kanai, A. Takahashi, Y. Momozawa, S. Ikegawa, M. Ikeda, N. Iwata, M. Hirata, K. Matsuda, Y. Murakami, M. Kubo, Y. Kamatani, Y. Okada, GWAS of 165,084 Japanese individuals identified nine loci associated with dietary habits. *Nat. Hum. Behav.* **4**, 308–316 (2020).

15. N. Druesne-Pecollo, B. Tehard, Y. Mallet, M. Gerber, T. Norat, S. Hercberg, P. Latino-Martel, Alcohol and genetic polymorphisms: Effect on risk of alcohol-related cancer. *Lancet Oncol.* **10**, 173–180 (2009).

16. C. H. Chen, J. C. Ferreira, E. R. Gross, D. Mochly-Rosen, Targeting aldehyde dehydrogenase 2: New therapeutic opportunities. *Physiol. Rev.* **94**, 1–34 (2014).

17. S. A. Marchitti, C. Brocker, D. Stagos, V. Vasiliou, Non-P450 aldehyde oxidizing enzymes: The aldehyde dehydrogenase superfamily. *Expert Opin. Drug Metab. Toxicol.* **4**, 697–720 (2008).

18. Y. N. Koyanagi, E. Suzuki, I. Imoto, Y. Kasugai, I. Oze, T. Ugai, M. Iwase, Y. Usui, Y. Kawakatsu, M. Sawabe, Y. Hirayama, T. Tanaka, T. Abe, S. Ito, K. Komori, N. Hanai, M. Tajika, Y. Shimizu, Y. Niwa, H. Ito, K. Matsuo, Across-site differences in the mechanism of alcohol-induced digestive tract carcinogenesis: An evaluation by mediation analysis. *Cancer Res.* **80**, 1601–1610 (2020).

19. C. C. Chen, R. B. Lu, Y. C. Chen, M. F. Wang, Y. C. Chang, T. K. Li, S. J. Yin, Interaction between the functional polymorphisms of the alcohol-metabolism genes in protection against alcoholism. *Am. J. Hum. Genet.* **65**, 795–807 (1999).

20. K. Matsuo, K. Wakai, K. Hirose, H. Ito, T. Saito, K. Tajima, Alcohol dehydrogenase 2 His^{47}Arg polymorphism influences drinking habit independently of aldehyde dehydrogenase ^{2}Glu^{487}Lys polymorphism: Analysis of 2,299 Japanese subjects. *Cancer Epidemiol. Biomarkers Prev.* **15**, 1009–1013 (2006).

21. C. L. Lai, C. T. Yao, G. Y. Chau, L. F. Yang, T. Y. Kuo, C. P. Chiang, S. J. Yin, Dominance of the inactive Asian variant over activity and protein contents of mitochondrial aldehyde dehydrogenase 2 in human liver. *Alcohol. Clin. Exp. Res.* **38**, 44–50 (2014).

22. X. Wang, S. Sheikh, D. Saigal, L. Robinson, H. Weiner, Heterotetramers of human liver mitochondrial (class 2) aldehyde dehydrogenase expressed in *Escherichia coli*. A model to study the heterotetramers expected to be found in Oriental people. *J. Biol. Chem.* **271**, 31172–31178 (1996).

23. J. S. Chang, J. R. Hsiao, C. H. Chen, ALDH2 polymorphism and alcohol-related cancers in Asians: A public health perspective. *J. Biomed. Sci.* **24**, 19 (2017).

24. Y. N. Koyanagi, H. Ito, I. Oze, S. Hosono, H. Tanaka, T. Abe, Y. Shimizu, Y. Hasegawa, K. Matsuo, Development of a prediction model and estimation of cumulative risk for upper aerodigestive tract cancer on the basis of the aldehyde dehydrogenase 2 genotype and alcohol consumption in a Japanese population. *Eur. J. Cancer Prev.* **26**, 38–47 (2017).

25. N. Hamajima, K. Matsuo, T. Saito, K. Hirose, M. Inoue, T. Takezaki, T. Kuroishi, K. Tajima, Gene-environment interactions and polymorphism studies of cancer risk in the Hospital-based Epidemiologic Research Program at Aichi Cancer Center II (HERPACC-II). *Asian Pac. J. Cancer Prev.* **2**, 99–107 (2001).

26. N. Hamajima, J-MICC Study Group, The Japan Multi-Institutional Collaborative Cohort Study (J-MICC Study) to detect gene-environment interactions for cancer. *Asian Pac. J. Cancer Prev.* **8**, 317–323 (2007).

27. K. Wakai, N. Hamajima, R. Okada, M. Naito, E. Morita, A. Hishida, S. Kawai, K. Nishio, G. Yin, Y. Asai, K. Matsuo, S. Hosono, H. Ito, M. Watanabe, T. Kawase, T. Suzuki, K. Tajima, K. Tanaka, Y. Higaki, M. Hara, T. Imaizumi, N. Taguchi, K. Nakamura, H. Nanri, T. Sakamoto, M. Horita, K. Shinchi, Y. Kita, T. C. Turin, N. Rumana, K. Matsui, K. Miura, H. Ueshima, N. Takashima, Y. Nakamura, S. Suzuki, R. Ando, A. Hosono, N. Imaeda, K. Shibata, C. Goto, N. Hattori, M. Fukatsu, T. Yamada, S. Tokudome, T. Takezaki, H. Niimura, K. Hirasada, A. Nakamura, M. Tatebo, S. Ogawa, N. Tsunematsu, S. Chiba, H. Mikami, S. Kono, K. Ohnaka,

R. Takayanagi, Y. Watanabe, E. Ozaki, M. Shigeta, N. Kuriyama, A. Yoshikawa, D. Matsui, I. Watanabe, K. Inoue, K. Ozasa, S. Mitani, K. Arisawa, H. Uemura, M. Hiyoshi, H. Takami, M. Yamaguchi, M. Nakamoto, H. Takeda, M. Kubo, H. Tanaka, J-MICC Study Group, Profile of participants and genotype distributions of 108 polymorphisms in a cross-sectional study of associations of genotypes with lifestyle and clinical factors: A project in the Japan Multi-Institutional Collaborative Cohort (J-MICC) Study. *J. Epidemiol.* **21**, 223–235 (2011).

28. S. Tsugane, N. Sawada, The JPHC study: Design and some findings on the typical Japanese diet. *Jpn. J. Clin. Oncol.* **44**, 777–782 (2014).

29. A. Hozawa, K. Tanno, N. Nakaya, T. Nakamura, N. Tsuchiya, T. Hirata, A. Narita, M. Kogure, K. Nochioka, R. Sasaki, N. Takanashi, K. Otsuka, K. Sakata, S. Kuriyama, M. Kikuya, O. Tanabe, J. Sugawara, K. Suzuki, Y. Suzuki, E. N. Kodama, N. Fuse, H. Kiyomoto, H. Tomita, A. Uruno, Y. Hamanaka, H. Metoki, M. Ishikuro, T. Obara, T. Kobayashi, K. Kitatani, T. Takai-Igarashi, S. Ogishima, M. Satoh, H. Ohmomo, A. Tsuboi, S. Egawa, T. Ishii, K. Ito, S. Ito, Y. Taki, N. Minegishi, N. Ishii, M. Nagasaki, K. Igarashi, S. Koshiba, R. Shimizu, G. Tamiya, K. Nakayama, H. Motohashi, J. Yasuda, A. Shimizu, T. Hachiya, Y. Shiwa, T. Tominaga, H. Tanaka, K. Oyama, R. Tanaka, H. Kawame, A. Fukushima, Y. Ishigaki, T. Tokutomi, N. Osumi, T. Kobayashi, F. Nagami, H. Hashizume, T. Arai, Y. Kawaguchi, S. Higuchi, M. Sakaida, R. Endo, S. Nishizuka, I. Tsuji, J. Hitomi, M. Nakamura, K. Ogasawara, N. Yaegashi, K. Kinoshita, S. Kure, A. Sakai, S. Kobayashi, K. Sobue, M. Sasaki, M. Yamamoto, Study profile of the tohoku medical megabank community-based cohort study. *J. Epidemiol.* **31**, 65–76 (2021).

30. S. Funada, T. Kawaguchi, N. Terada, H. Negoro, Y. Tabara, S. Kosugi, R. Yamada, T. Nakayama, S. Akamatsu, K. Yoshimura, F. Matsuda, O. Ogawa, Nagahama Study Group, Cross-sectional epidemiological analysis of the Nagahama study for correlates of overactive bladder: Genetic and environmental considerations. *J. Urol.* **199**, 774–778 (2018).

31. M. Hirata, Y. Kamatani, A. Nagai, Y. Kiyohara, T. Ninomiya, A. Tamakoshi, Z. Yamagata, M. Kubo, K. Muto, T. Mushiroda, Y. Murakami, K. Yuji, Y. Furukawa, H. Zembutsu, T. Tanaka, Y. Ohnishi, Y. Nakamura, BioBank Japan Cooperative Hospital Group, K. Matsuda, Cross-

sectional analysis of BioBank Japan clinical data: A large cohort of 200,000 patients with 47 common diseases. *J. Epidemiol.* **27**, S9–S21 (2017).

32. A. Nagai, M. Hirata, Y. Kamatani, K. Muto, K. Matsuda, Y. Kiyohara, T. Ninomiya, A. Tamakoshi, Z. Yamagata, T. Mushiroda, Y. Murakami, K. Yuji, Y. Furukawa, H. Zembutsu, T. Tanaka, Y. Ohnishi, Y. Nakamura, BioBank Japan Cooperative Hospital Group, M. Kubo, Overview of the BioBank Japan Project: Study design and profile. *J. Epidemiol.* **27**, S2–S8 (2017).

33. B. K. Bulik-Sullivan, P.-R. Loh, H. K. Finucane, S. Ripke, J. Yang, Schizophrenia Working Group of the Psychiatric Genomics Consortium, N. Patterson, M. J. Daly, A. L. Price, B. M. Neale, LD score regression distinguishes confounding from polygenicity in genome-wide association studies. *Nat. Genet.* **47**, 291–295 (2015).

34. S. E. Luczak, S. J. Glatt, T. L. Wall, Meta-analyses of ALDH2 and ADH1B with alcohol dependence in Asians. *Psychol. Bull.* **132**, 607–621 (2006).

35. T. W. Winkler, F. R. Day, D. C. Croteau-Chonka, A. R. Wood, A. E. Locke, R. Mägi, T. Ferreira, T. Fall, M. Graff, A. E. Justice, J. an Luan, S. Gustafsson, J. C. Randall, S. Vedantam, T. Workalemahu, T. O. Kilpeläinen, A. Scherag, T. Esko, Z. Kutalik, I. M. Heid, R. J. F. Loos, Genetic Investigation of Anthropometric Traits (GIANT) Consortium, Quality control and conduct of genome-wide association meta-analyses. *Nat. Protoc.* **9**, 1192–1212 (2014).

36. B. Han, E. Eskin, Random-effects model aimed at discovering associations in meta-analysis of genome-wide association studies. *Am. J. Hum. Genet.* **88**, 586–598 (2011).

37. A. K. Manning, M. LaValley, C. T. Liu, K. Rice, P. An, Y. Liu, I. Miljkovic, L. Rasmussen-Torvik, T. B. Harris, M. A. Province, I. B. Borecki, J. C. Florez, J. B. Meigs, L. A. Cupples, J. Dupuis, Meta-analysis of gene-environment interaction: Joint estimation of SNP and SNP × environment regression coefficients. *Genet. Epidemiol.* **35**, 11–18 (2011).

38. J. Gelernter, H. R. Kranzler, R. Sherva, L. Almasy, R. Koesterer, A. H. Smith, R. Anton, U. W. Preuss, M. Ridinger, D. Rujescu, N. Wodarz, P. Zill, H. Zhao, L. A. Farrer, Genome-wide

association study of alcohol dependence:significant findings in African- and European-Americans including novel risk loci. *Mol. Psychiatry* **19**, 41–49 (2014).

39. L. Zuo, K. Wang, X. Y. Zhang, J. H. Krystal, C. S. Li, F. Zhang, H. Zhang, X. Luo, NKAIN1-SERINC2 is a functional, replicable and genome-wide significant risk gene region specific for alcohol dependence in subjects of European descent. *Drug Alcohol Depend.* **129**, 254–264 (2013).

40. B. C. Brown, Asian Genetic Epidemiology Network Type 2 Diabetes Consortium, C. J. Ye, A. L. Price, N. Zaitlen, Transethnic genetic-correlation estimates from summary statistics. *Am. J. Hum. Genet.* **99**, 76–88 (2016).

41. H. K. Seitz, F. Stickel, Molecular mechanisms of alcohol-mediated carcinogenesis. *Nat. Rev. Cancer* **7**, 599–612 (2007).

42. M. J. Knol, T. J. VanderWeele, Recommendations for presenting analyses of effect modification and interaction. *Int. J. Epidemiol.* **41**, 514–520 (2012).

43. W. Zhou, J. B. Nielsen, L. G. Fritsche, R. Dey, M. E. Gabrielsen, B. N. Wolford, J. LeFaive, P. VandeHaar, S. A. Gagliano, A. Gifford, L. A. Bastarache, W. Q. Wei, J. C. Denny, M. Lin, K. Hveem, H. M. Kang, G. R. Abecasis, C. J. Willer, S. Lee, Efficiently controlling for case-control imbalance and sample relatedness in large-scale genetic association studies. *Nat. Genet.* **50**, 1335–1341 (2018).

44. J. Mbatchou, L. Barnard, J. Backman, A. Marcketta, J. A. Kosmicki, A. Ziyatdinov, C. Benner, C. O'Dushlaine, M. Barber, B. Boutkov, L. Habegger, M. Ferreira, A. Baras, J. Reid, G. Abecasis, E. Maxwell, J. Marchini, Computationally efficient whole-genome regression for quantitative and binary traits. *Nat. Genet.* **53**, 1097–1103 (2021).

45. L. L. Husemoen, M. Fenger, N. Friedrich, J. S. Tolstrup, S. Beenfeldt Fredriksen, A. Linneberg, The association of ADH and ALDH gene variants with alcohol drinking habits and cardiovascular disease risk factors. *Alcohol. Clin. Exp. Res.* **32**, 1984–1991 (2008).

46. A. Linneberg, A. Gonzalez-Quintela, C. Vidal, T. Jorgensen, M. Fenger, T. Hansen, O. Pedersen, L. L. Husemoen, Genetic determinants of both ethanol and acetaldehyde metabolism influence alcohol hypersensitivity and drinking behaviour among Scandinavians. *Clin. Exp. Allergy* **40**, 123–130 (2010).

47. M. J. Way, M. A. Ali, A. McQuillin, M. Y. Morgan, Genetic variants in ALDH1B1 and alcohol dependence risk in a British and Irish population: A bioinformatic and genetic study. *PLOS ONE* **12**, e0177009 (2017).

48. B. C. Jackson, P. Reigan, B. Miller, D. C. Thompson, V. Vasiliou, Human ALDH1B1 polymorphisms may affect the metabolism of acetaldehyde and all-trans retinaldehyde—In vitro studies and computational modeling. *Pharm. Res.* **32**, 1648–1662 (2015).

49. H. N. Larson, H. Weiner, T. D. Hurley, Disruption of the coenzyme binding site and dimer interface revealed in the crystal structure of mitochondrial aldehyde dehydrogenase "Asian" variant. *J. Biol. Chem.* **280**, 30550–30556 (2005).

50. H. Oota, A. J. Pakstis, B. Bonne-Tamir, D. Goldman, E. Grigorenko, S. L. Kajuna, N. J. Karoma, S. Kungulilo, R. B. Lu, K. Odunsi, F. Okonofua, O. V. Zhukova, J. R. Kidd, K. K. Kidd, The evolution and population genetics of the ALDH2 locus: Random genetic drift, selection, and low levels of recombination. *Ann. Hum. Genet.* **68**, 93–109 (2004).

51. Y. Okada, Y. Momozawa, S. Sakaue, M. Kanai, K. Ishigaki, M. Akiyama, T. Kishikawa, Y. Arai, T. Sasaki, K. Kosaki, M. Suematsu, K. Matsuda, K. Yamamoto, M. Kubo, N. Hirose, Y. Kamatani, Deep whole-genome sequencing reveals recent selection signatures linked to evolution and disease risk of Japanese. *Nat. Commun.* **9**, 1631 (2018).

52. M. Kanai, M. Akiyama, A. Takahashi, N. Matoba, Y. Momozawa, M. Ikeda, N. Iwata, S. Ikegawa, M. Hirata, K. Matsuda, M. Kubo, Y. Okada, Y. Kamatani, Genetic analysis of quantitative traits in the Japanese population links cell types to complex human diseases. *Nat. Genet.* **50**, 390–400 (2018).

53. S. L. Zhou, R. E. Gordon, M. Bradbury, D. Stump, C. L. Kiang, P. D. Berk, Ethanol up-regulates fatty acid uptake and plasma membrane expression and export of mitochondrial aspartate aminotransferase in HepG2 cells. *Hepatology* **27**, 1064–1074 (1998).

54. T. Takeshita, X. Yang, K. Morimoto, The ALDH2 genotype, alcohol intake, and liver-function biomarkers among Japanese male workers. *Hum. Genet.* **106**, 589–593 (2000).

55. N. Nagata, N. Watanabe, M. Tsuda, H. Tsukamoto, S. Matsuzaki, Relationship between serum levels of anti-low-density lipoprotein-acetaldehyde-adduct antibody and aldehyde dehydrogenase 2 heterozygotes in patients with alcoholic liver injury. *Alcohol. Clin. Exp. Res.* **23**, 24S–28S (1999).

56. H. J. Kwon, Y. S. Won, O. Park, B. Chang, M. J. Duryee, G. E. Thiele, A. Matsumoto, S. Singh, M. A. Abdelmegeed, B. J. Song, T. Kawamoto, V. Vasiliou, G. M. Thiele, B. Gao, Aldehyde dehydrogenase 2 deficiency ameliorates alcoholic fatty liver but worsens liver inflammation and fibrosis in mice. *Hepatology* **60**, 146–157 (2014).

57. G. R. B. Saunders, X. Wang, F. Chen, S.-K. Jang, M. Liu, C. Wang, S. Gao, Y. Jiang, C. Khunsriraksakul, J. M. Otto, C. Addison, M. Akiyama, C. M. Albert, F. Aliev, A. Alonso, D. K. Arnett, A. E. Ashley-Koch, A. A. Ashrani, K. C. Barnes, R. Graham Barr, T. M. Bartz, D. M. Becker, L. F. Bielak, E. J. Benjamin, J. C. Bis, G. Bjornsdottir, J. Blangero, E. R. Bleecker, J. D. Boardman, E. Boerwinkle, D. I. Boomsma, M. P. Boorgula, D. W. Bowden, J. A. Brody, B. E. Cade, D. I. Chasman, S. Chavan, Yii-Der Ida Chen, Z. Chen, I. Cheng, M. H. Cho, H. Choquet, J. W. Cole, M. C. Cornelis, F. Cucca, J. E. Curran, M. de Andrade, D. M. Dick, A. R. Docherty, R. Duggirala, C. B. Eaton, M. A. Ehringer, T. Esko, J. D. Faul, L. F. Silva, E. Fiorillo, M. Fornage, B. I. Freedman, M. E. Gabrielsen, M. E. Garrett, S. A. Gharib, C. Gieger, N. Gillespie, D. C. Glahn, S. D. Gordon, C. C. Gu, D. Gu, D. F. Gudbjartsson, X. Guo, J. Haessler, M. E. Hall, T. Haller, K. M. Harris, J. He, P. Herd, J. K. Hewitt, I. Hickie, B. Hidalgo, J. E. Hokanson, C. Hopfer, J. J. Hottenga, L. Hou, H. Huang, Y.-J. Hung, D. J. Hunter, K. Hveem, S.-J. Hwang, C.-M. Hwu, W. Iacono, M. R. Irvin, Y. H. Jee, E. O. Johnson, Y. Y. Joo, E. Jorgenson, A. E. Justice, Y. Kamatani, R. C. Kaplan, J. Kaprio, S. L. R. Kardia, M. C. Keller, T. N. Kelly, C. Kooperberg, T. Korhonen, P. Kraft, K. Krauter, J. Kuusisto, M. Laakso,

J. Lasky-Su, W.-J. Lee, J. J. Lee, D. Levy, L. Li, K. Li, Y. Li, K. Lin, P. A. Lind, C. Liu, D. M. Lloyd-Jones, S. M. Lutz, J. Ma, R. Mägi, A. Manichaikul, N. G. Martin, R. Mathur, N. Matoba, Patrick F. Mc Ardle, M. M. Gue, Matthew B. Mc Queen, S. E. Medland, A. Metspalu, D. A. Meyers, I. Y. Millwood, B. D. Mitchell, K. L. Mohlke, M. Moll, M. E. Montasser, A. C. Morrison, A. Mulas, J. B. Nielsen, K. E. North, E. C. Oelsner, Y. Okada, V. Orrù, N. D. Palmer, T. Palviainen, A. Pandit, S. Lani Park, U. Peters, A. Peters, P. A. Peyser, T. J. C. Polderman, N. Rafaels, S. Redline, R. M. Reed, A. P. Reiner, J. P. Rice, S. S. Rich, N. E. Richmond, C. Roan, J. I. Rotter, M. N. Rueschman, V. Runarsdottir, N. L. Saccone, D. A. Schwartz, A. H. Shadyab, J. Shi, S. S. Shringarpure, K. Sicinski, A. H. Skogholt, J. A. Smith, N. L. Smith, N. Sotoodehnia, M. C. Stallings, H. Stefansson, K. Stefansson, J. A. Stitzel, X. Sun, M. Syed, R. Tal-Singer, A. E. Taylor, K. D. Taylor, M. J. Telen, K. K. Thai, H. Tiwari, C. Turman, T. Tyrfingsson, T. L. Wall, R. G. Walters, D. R. Weir, S. T. Weiss, W. B. White, J. B. Whitfield, K. L. Wiggins, G. Willemsen, C. J. Willer, B. S. Winsvold, H. Xu, L. R. Yanek, J. Yin, K. L. Young, K. A. Young, B. Yu, W. Zhao, W. Zhou, S. Zöllner, L. Zuccolo, 23andMe Research Team, The Biobank Japan Project, C. Batini, A. W. Bergen, L. J. Bierut, S. P. David, Sarah A. Gagliano Taliun, D. B. Hancock, B. Jiang, M. R. Munafò, T. E. Thorgeirsson, D. J. Liu, S. Vrieze, Genetic diversity fuels gene discovery for tobacco and alcohol use. *Nature* **612**, 720–724 (2022).

58. R. Cui, Y. Kamatani, A. Takahashi, M. Usami, N. Hosono, T. Kawaguchi, T. Tsunoda, N. Kamatani, M. Kubo, Y. Nakamura, K. Matsuda, Functional variants in ADH1B and ALDH2 coupled with alcohol and smoking synergistically enhance esophageal cancer risk. *Gastroenterology* **137**, 1768–1775 (2009).

59. C. Wu, P. Kraft, K. Zhai, J. Chang, Z. Wang, Y. Li, Z. Hu, Z. He, W. Jia, C. C. Abnet, L. Liang, N. Hu, X. Miao, Y. Zhou, Z. Liu, Q. Zhan, Y. Liu, Y. Qiao, Y. Zhou, G. Jin, C. Guo, C. Lu, H. Yang, J. Fu, D. Yu, N. D. Freedman, T. Ding, W. Tan, A. M. Goldstein, T. Wu, H. Shen, Y. Ke, Y. Zeng, S. J. Chanock, P. R. Taylor, D. Lin, Genome-wide association analyses of esophageal squamous cell carcinoma in Chinese identify multiple susceptibility loci and gene-environment interactions. *Nat. Genet.* **44**, 1090–1097 (2012).

60. R. C. Engs, D. J. Hanson, L. Gliksman, C. Smythe, Influence of religion and culture on drinking behaviours: A test of hypotheses between Canada and the USA. *Br. J. Addict.* **85**, 1475–1482 (1990).

61. I. Y. Millwood, L. Li, M. Smith, Y. Guo, L. Yang, Z. Bian, S. Lewington, G. Whitlock, P. Sherliker, R. Collins, J. Chen, R. Peto, H. Wang, J. Xu, J. He, M. Yu, H. Liu, Z. Chen, China Kadoorie Biobank collaborative group, Alcohol consumption in 0.5 million people from 10 diverse regions of China: Prevalence, patterns and socio-demographic and health-related correlates. *Int. J. Epidemiol.* **42**, 816–827 (2013).

62. T. L. Wall, S. E. Luczak, S. Hiller-Sturmhöfel, Biology, genetics, and environment: Underlying factors influencing alcohol metabolism. *Alcohol Res.* **38**, 59–68 (2016).

63. S. Higuchi, K. M. Parrish, M. C. Dufour, L. H. Towle, T. C. Harford, Relationship between age and drinking patterns and drinking problems among Japanese, Japanese-Americans, and Caucasians. *Alcohol. Clin. Exp. Res.* **18**, 305–310 (1994).

64. Q. Sun, M. Graff, B. Rowland, J. Wen, L. Huang, T. W. Miller-Fleming, J. Haessler, M. H. Preuss, J. F. Chai, M. P. Lee, C. L. Avery, C. Y. Cheng, N. Franceschini, X. Sim, N. J. Cox, C. Kooperberg, K. E. North, Y. Li, L. M. Raffield, Analyses of biomarker traits in diverse UK biobank participants identify associations missed by European-centric analysis strategies. *J. Hum. Genet.* **67**, 87–93 (2022).

65. C. C. Spencer, Z. Su, P. Donnelly, J. Marchini, Designing genome-wide association studies: Sample size, power, imputation, and the choice of genotyping chip. *PLOS Genet.* **5**, e1000477 (2009).

66. F. A. Dingler, M. Wang, A. Mu, C. L. Millington, N. Oberbeck, S. Watcham, L. B. Pontel, A. N. Kamimae-Lanning, F. Langevin, C. Nadler, R. L. Cordell, P. S. Monks, R. Yu, N. K. Wilson, A. Hira, K. Yoshida, M. Mori, Y. Okamoto, Y. Okuno, H. Muramatsu, Y. Shiraishi, M. Kobayashi, T. Moriguchi, T. Osumi, M. Kato, S. Miyano, E. Ito, S. Kojima, H. Yabe, M. Yabe, K. Matsuo, S. Ogawa, B. Gottgens, M. R. G. Hodskinson, M. Takata, K. J. Patel, Two aldehyde

clearance systems are essential to prevent lethal formaldehyde accumulation in mice and humans. *Mol. Cell* **80**, 996–1012.e9 (2020).

67. S. Suzuki, A. Goto, M. Nakatochi, A. Narita, T. Yamaji, N. Sawada, R. Katagiri, M. Iwagami, A. Hanyuda, T. Hachiya, Y. Sutoh, I. Oze, Y. N. Koyanagi, Y. Kasugai, Y. Taniyama, H. Ito, H. Ikezaki, Y. Nishida, T. Tamura, H. Mikami, T. Takezaki, S. Suzuki, E. Ozaki, K. Kuriki, N. Takashima, K. Arisawa, K. Takeuchi, K. Tanno, A. Shimizu, G. Tamiya, A. Hozawa, K. Kinoshita, K. Wakai, M. Sasaki, M. Yamamoto, K. Matsuo, S. Tsugane, M. Iwasaki, Body mass index and colorectal cancer risk: A Mendelian randomization study. *Cancer Sci.* **112**, 1579–1588 (2021).

68. 1000 Genomes Project Consortium, A. Auton, L. D. Brooks, R. M. Durbin, E. P. Garrison, H. M. Kang, J. O. Korbel, J. L. Marchini, S. M. Carthy, G. A. Mc Vean, G. R. Abecasis, A global reference for human genetic variation. *Nature* **526**, 68–74 (2015).

69. O. Delaneau, J. F. Zagury, J. Marchini, Improved whole-chromosome phasing for disease and population genetic studies. *Nat. Methods* **10**, 5–6 (2013).

70. P. R. Loh, P. F. Palamara, A. L. Price, Fast and accurate long-range phasing in a UK Biobank cohort. *Nat. Genet.* **48**, 811–816 (2016).

71. S. Das, L. Forer, S. Schonherr, C. Sidore, A. E. Locke, A. Kwong, S. I. Vrieze, E. Y. Chew, S. Levy, M. McGue, D. Schlessinger, D. Stambolian, P. R. Loh, W. G. Iacono, A. Swaroop, L. J. Scott, F. Cucca, F. Kronenberg, M. Boehnke, G. R. Abecasis, C. Fuchsberger, Next-generation genotype imputation service and methods. *Nat. Genet.* **48**, 1284–1287 (2016).

72. B. N. Howie, P. Donnelly, J. Marchini, A flexible and accurate genotype imputation method for the next generation of genome-wide association studies. *PLOS Genet.* **5**, e1000529 (2009).

73. J. Marchini, B. Howie, S. Myers, G. McVean, P. Donnelly, A new multipoint method for genome-wide association studies by imputation of genotypes. *Nat. Genet.* **39**, 906–913 (2007).

74. C. C. Chang, C. C. Chow, L. C. Tellier, S. Vattikuti, S. M. Purcell, J. J. Lee, Second-generation PLINK: Rising to the challenge of larger and richer datasets. *Gigascience* **4**, 7 (2015).

75. C. J. Willer, Y. Li, G. R. Abecasis, METAL: Fast and efficient meta-analysis of genomewide association scans. *Bioinformatics* **26**, 2190–2191 (2010).

76. M. L. Freedman, D. Reich, K. L. Penney, G. J. McDonald, A. A. Mignault, N. Patterson, S. B. Gabriel, E. J. Topol, J. W. Smoller, C. N. Pato, M. T. Pato, T. L. Petryshen, L. N. Kolonel, E. S. Lander, P. Sklar, B. Henderson, J. N. Hirschhorn, D. Altshuler, Assessing the impact of population stratification on genetic association studies. *Nat. Genet.* **36**, 388–393 (2004).

77. H. Aschard, D. B. Hancock, S. J. London, P. Kraft, Genome-wide meta-analysis of joint tests for genetic and gene-environment interaction effects. *Hum. Hered.* **70**, 292–300 (2010).

78. K. E. Westerman, D. T. Pham, L. Hong, Y. Chen, M. Sevilla-Gonzalez, Y. J. Sung, Y. V. Sun, A. C. Morrison, H. Chen, A. K. Manning, GEM: Scalable and flexible gene-environment interaction analysis in millions of samples. *Bioinformatics* **37**, 3514–3520 (2021).

79. J. Yang, T. Ferreira, A. P. Morris, S. E. Medland, Genetic Investigation of ANthropometric Traits (GIANT) Consortium, DIAbetes Genetics Replication And Meta-analysis (DIAGRAM) Consortium, P. A. F. Madden, A. C. Heath, N. G. Martin, G. W. Montgomery, M. N. Weedon, R. J. Loos, T. M. Frayling, M. I. Mc Carthy, J. N. Hirschhorn, M. E. Goddard, P. M. Visscher, Conditional and joint multiple-SNP analysis of GWAS summary statistics identifies additional variants influencing complex traits. *Nat. Genet.* **44**, 369–375 (2012).

80. K. Wang, M. Li, H. Hakonarson, ANNOVAR: Functional annotation of genetic variants from high-throughput sequencing data. *Nucleic Acids Res.* **38**, e164 (2010).

81. The GTEx Consortium, The GTEx Consortium atlas of genetic regulatory effects across human tissues. *Science* **369**, 1318–1330 (2020).

82. J. C. Barrett, B. Fry, J. Maller, M. J. Daly, Haploview: Analysis and visualization of LD and haplotype maps. *Bioinformatics* **21**, 263–265 (2005).

83. H. Ito, J. D. McKay, S. Hosono, T. Hida, Y. Yatabe, T. Mitsudomi, P. Brennan, H. Tanaka, K. Matsuo, Association between a genome-wide association study-identified locus and the risk of lung cancer in Japanese population. *J. Thorac. Oncol.* **7**, 790–798 (2012).

84. A. Manichaikul, J. C. Mychaleckyj, S. S. Rich, K. Daly, M. Sale, W. M. Chen, Robust relationship inference in genome-wide association studies. *Bioinformatics* **26**, 2867–2873 (2010).

85. World Health Organization, *International Classification of Diseases for Oncology* (World Health Organization, ed. 3, 2000).

86. R. DerSimonian, N. Laird, Meta-analysis in clinical trials revisited. *Contemp. Clin. Trials* **45**, 139–145 (2015).

87. J. P. Higgins, S. G. Thompson, Quantifying heterogeneity in a meta-analysis. *Stat. Med.* **21**, 1539–1558 (2002).

88. I. R. White, Multivariate random-effects meta-analysis *Stata J.* **9**, 40–56 (2009).

89. T. J. VanderWeele, M. J. Knol, A tutorial on interaction *Epidemiol. Methods* **3**, 33–72 (2014).

90. D. W. Hosmer, S. Lemeshow, Confidence interval estimation of interaction. *Epidemiology* **3**, 452–456 (1992).

91. M. Sakurai-Yageta, K. Kumada, C. Gocho, S. Makino, A. Uruno, S. Tadaka, I. N. Motoike, M. Kimura, S. Ito, A. Otsuki, A. Narita, H. Kudo, Y. Aoki, I. Danjoh, J. Yasuda, H. Kawame, N. Minegishi, S. Koshiba, N. Fuse, G. Tamiya, M. Yamamoto, K. Kinoshita, Japonica Array NEO with increased genome-wide coverage and abundant disease risk SNPs. *J. Biochem.* **170**, 399–410 (2021).

92. M. Yamada, I. N. Motoike, K. Kojima, N. Fuse, A. Hozawa, S. Kuriyama, F. Katsuoka, S. Tadaka, M. Shirota, M. Sakurai, T. Nakamura, Y. Hamanaka, K. Suzuki, J. Sugawara, S. Ogishima, A. Uruno, E. N. Kodama, N. Fujino, T. Numakura, T. Ichikawa, A. Mitsune, T. Ohe, K. Kinoshita, M. Ichinose, H. Sugiura, M. Yamamoto, Genetic loci for lung function in

Japanese adults with adjustment for exhaled nitric oxide levels as airway inflammation indicator. *Commun. Biol.* **4**, 1288 (2021).

93. S. W. Choi, P. F. O'Reilly, PRSice-2: Polygenic Risk Score software for biobank-scale data. *Gigascience* **8**, giz082 (2019).

94. S. Kuriyama, N. Yaegashi, F. Nagami, T. Arai, Y. Kawaguchi, N. Osumi, M. Sakaida, Y. Suzuki, K. Nakayama, H. Hashizume, G. Tamiya, H. Kawame, K. Suzuki, A. Hozawa, N. Nakaya, M. Kikuya, H. Metoki, I. Tsuji, N. Fuse, H. Kiyomoto, J. Sugawara, A. Tsuboi, S. Egawa, K. Ito, K. Chida, T. Ishii, H. Tomita, Y. Taki, N. Minegishi, N. Ishii, J. Yasuda, K. Igarashi, R. Shimizu, M. Nagasaki, S. Koshiba, K. Kinoshita, S. Ogishima, T. Takai-Igarashi, T. Tominaga, O. Tanabe, N. Ohuchi, T. Shimosegawa, S. Kure, H. Tanaka, S. Ito, J. Hitomi, K. Tanno, M. Nakamura, K. Ogasawara, S. Kobayashi, K. Sakata, M. Satoh, A. Shimizu, M. Sasaki, R. Endo, K. Sobue, The Tohoku Medical Megabank Project Study Group, M. Yamamoto, The Tohoku Medical Megabank Project: Design and mission. *J. Epidemiol.* **26**, 493–511 (2016).

95. M. Akiyama, K. Ishigaki, S. Sakaue, Y. Momozawa, M. Horikoshi, M. Hirata, K. Matsuda, S. Ikegawa, A. Takahashi, M. Kanai, S. Suzuki, D. Matsui, M. Naito, T. Yamaji, M. Iwasaki, N. Sawada, K. Tanno, M. Sasaki, A. Hozawa, N. Minegishi, K. Wakai, S. Tsugane, A. Shimizu, M. Yamamoto, Y. Okada, Y. Murakami, M. Kubo, Y. Kamatani, Characterizing rare and low-frequency height-associated variants in the Japanese population. *Nat. Commun.* **10**, 4393 (2019).

96. Y. Tsubono, M. Kobayashi, S. Sasaki, S. Tsugane, JPHC, Validity and reproducibility of a self-administered food frequency questionnaire used in the baseline survey of the JPHC Study Cohort I. *J. Epidemiol.* **13**, S125–S133 (2003).

97. K. Yamamoto, K. Sonehara, S. Namba, T. Konuma, H. Masuko, S. Miyawaki, The BioBank Japan Project, Y. Kamatani, N. Hizawa, K. Ozono, L. Yengo, Y. Okada, Genetic footprints of assortative mating in the Japanese population. *Nat. Hum. Behav.* **7**, 65–73 (2023).

98. H. Han, H. Wang, Z. Yin, H. Jiang, M. Fang, J. Han, Association of genetic polymorphisms in ADH and ALDH2 with risk of coronary artery disease and myocardial infarction: A meta-analysis. *Gene* **526**, 134–141 (2013).

99. Y. Yamada, K. Kato, M. Oguri, H. Horibe, T. Fujimaki, Y. Yasukochi, I. Takeuchi, J. Sakuma, Identification of 13 novel susceptibility loci for early-onset myocardial infarction, hypertension, or chronic kidney disease. *Int. J. Mol. Med.* **42**, 2415–2436 (2018).

100. D. P. Leong, A. Smyth, K. K. Teo, M. M. Kee, S. Rangarajan, P. Pais, L. Liu, S. S. Anand, S. Yusuf, INTERHEART Investigators, Patterns of alcohol consumption and myocardial infarction risk. *Circulation* **130**, 390–398 (2014).
